# Rising temperatures, shifting population: Changes in children’s ambient heat exposure in China

**DOI:** 10.1073/pnas.2615409123

**Published:** 2026-11-02

**Authors:** Kai Feng, Marco M. Laghi, Jere R. Behrman, Emily Hannum, Fan Wang

**Affiliations:** ^a^ Max Planck Institute for Demographic Research, Rostock 18057, Germany; ^b^Max Planck-University of Helsinki Center for Social Inequalities in Population Health, Rostock 18057, Germany; ^c^ Department of Sociology, New York University, New York, NY 10003; ^d^ Center for Applied Social and Economic Research, New York University Shanghai, Shanghai 200124, China; ^e^ Department of Economics, University of Pennsylvania, Philadelphia, PA 19104; ^f^ Department of Sociology, University of Pennsylvania, Philadelphia, PA 19104; ^g^ Population Studies Center, University of Pennsylvania, Philadelphia, PA 19104; ^h^ Department of Economics, University of Houston, Houston, TX 77204

**Keywords:** extreme heat, children, China, geographic distribution of children

## Abstract

The frequency and intensity of heat waves are increasing, but how these trends translate to changes in the ambient heat to which children are potentially exposed (hereafter, exposures) is not well established. One complicating factor is that it is not only extreme temperatures that change over time, but also the geospatial distribution of children. Another limitation in our understanding is that existing studies often summarize exposure as person-time accumulated above a temperature cutoff, a quantity that confounds the choice of cutoff, the duration of individual exposure, and the share of the population affected. Our study addresses these gaps by developing a double-dual-distributional framework that jointly considers two types of geospatial and temporal distributions (ambient temperatures and child populations) and two types of heat exposure thresholds, namely intensity (heat levels) and duration (hourly exceedance). To illustrate, we assessed potential exposures—the ambient heat conditions experienced—among 250 million children in China, comparing the 1989–1991 and 2019–2021 periods. We found that children’s average annual exposure to moderate or stronger heat increased by 385 h, and the share exposed to strong heat for at least 12% of hours rose from 8.1% (24.9 million) to 23.7% (59.1 million). Both rising temperatures and shifts in child population distributions contribute materially to increased heat exposures in China, a finding that suggests the need for forecasts of children’s potential heat exposures to consider shifting child population distributions.

## Significance

Climate change is intensifying extreme heat worldwide. Concurrently, geospatial distributions of children are shifting due to demographic changes including urbanization. However, how these dual trends interact to shape children’s potential ambient heat exposure remains poorly understood. We introduce a framework that captures where and when children experience heat and accounts for both heat intensity and exposure duration. Comparing 1989–1991 and 2019–2021, we find sharp increases in intensity and duration of Chinese children’s heat exposure. These results show that rising temperatures and shifting populations amplify exposure risks. Our framework offers a simple, low-data-demand way to measure populations’ potential ambient heat exposure, illustrated here for children but applicable to any population subgroup, and supports better-informed strategies to protect children’s health.

Extreme heat exposure is a significant and growing threat to human health and welfare (1–5), with research increasingly highlighting its disproportionate impacts on vulnerable populations (6–10). Children are particularly susceptible to the detrimental effects of heat exposure because of their lower ability to self-thermoregulate, impaired thirst sensation, and impaired glomerular filtration rates (11–14). Studies have shown that extreme heat directly impacts children by undermining their nutrition (15), impairing cognition and skill development (11), and escalating rates of heat-related illnesses and mortality (13, 16). The negative effects of heat exposure on children may originate as early as the preconception period, with risks extending through the prenatal stage (17, 18). Research has linked high temperatures to increased instances of preterm births and low birth weights (19–21). Additionally, extreme heat indirectly affects children by exacerbating droughts and associated food insecurity (22–24), intensifying tropical disasters (25, 26), facilitating the spread of infectious diseases (27–30), and heightening the risk of violent conflicts (31, 32). Climatic change has both prolonged and intensified extreme heat exposures (33–37). Projections suggest a significant rise in the burden of heat exposures on populations, a trend attributed to both climatic changes and the increased populations exposed to these changes (38, 39). Yet, only a few studies focus on measuring exposure to heat among the child population despite the great vulnerability of children to heat exposures (40, 41). Notably, UNICEF has mapped child exposures to various hazards at the national level (40) and at the subnational level for the year 2024 (42), and is developing a new global Child Hazard Database that will permit subnational analysis (43, 44).

In addition, the prevalent approach in studies that measure general population heat burdens is to measure the total burden in person-time units, calculated by multiplying the total population by the time that the average person experiences heat exposures above designated thresholds (33, 35, 37, 39). While useful for capturing aggregate exposures, this method overlooks heterogeneities in intensity, duration, and risk levels, and obscures distributional inequalities by assuming uniform exposures across space, time, and demographic groups. This limitation is especially pronounced in contexts of high population mobility, where the spatial distribution of people relative to heat hazards evolves rapidly, making static exposure assumptions especially poor approximations of actual individual-level risk. Further, an overreliance on person-time units rather than individual-level exposure duration can be misleading in contexts of population decline, where aggregate exposure may decrease even as individual-level risk remains high. This issue is especially relevant for assessing heat exposure of the child population, which is shrinking in many countries, while growing in others (45).

Using the case of China—home to approximately 253.4 million children ages 0 to 14 in 2020 (46)—this paper provides empirical evidence on how heat exposures, across varying intensity and duration thresholds, have changed among children in recent decades. We propose a convenient, low-data-demand framework for measuring the share of children at risk of extreme-heat exposures. Using what we call the *double-dual-distributional* (DDD) framework, we measured population-level child-heat exposure changes by jointly considering two types of geospatial and temporal distributions—that of ambient temperatures and that of child populations—and two types of heat exposure thresholds—for intensity (heat exposure levels) and duration (hourly exceedance). In this framework, we compute a *Share of Time for the Average Child* (STAC) statistic, measuring the share of time exposed to extreme heat for the average child at or above any given heat threshold, and a *Share Exposed by Intensity and Duration Threshold* (SEIDT) statistic, measuring the share of children exposed to ambient extreme heat by differing intensity and duration thresholds.

Specifically, we merged child populations from 2,369 (year 1990) and 2,853 (year 2020) county-level administrative units with 15,432 grid points (0.25° × 0.25° spatial resolution) of hourly Universal Thermal Climate Index (UTCI) (47)—a composite thermal-stress metric integrating ambient air temperature, radiation, wind, and humidity—that overlap with the administrative boundary of mainland China (48). The wide dispersion of child populations across counties and the limited geographic expanse of most Chinese counties allow for the construction of granular child-population-weighted national and regional UTCI-based heat exposure statistics. Additionally, previous research highlights that heat effects depend on local population susceptibility, local climatology, and local adaptation (49, 50). To capture these variations, we provide both national and regional-level results. We run our analyses on both the single-year (1990 and 2020) and three-year (1989–1991 and 2019–2021) pooled UTCI data to better control for any single-year outliers from temperature trends.

We found substantial increases in the average ambient heat exposure for children and in the share of children exposed to ambient heat. Specifically, compared with 1989–1991, the average child’s potential exposure to at least moderate heat (i.e., UTCI ≽ 26 °C)—a level of heat that may cause thermal discomfort in healthy individuals and elevate health risks among vulnerable populations—increased by 385 h during 2019–2021. Furthermore, the share of children subjected to over 18 wk per year of such heat exposure more than doubled, increasing from 6.8% (21.0 million) to 14.2% (37.4 million). Finally, we highlighted significant regional disparities: In Eastern China, China’s most-developed region, there was a marked increase in the duration of children’s ambient heat exposure from 1989–1991 to 2019–2021, even though the exposure levels were already high in 1989–1991. Conversely, the Central region experienced only about half the increase in exposure duration over the same period compared to the Eastern region, despite the two regions having comparable exposure levels in 1989–1991.

## Results

### Increase in the Share of Time Exposed to Heat for the Average Child (STAC).

**Fig. 1.**
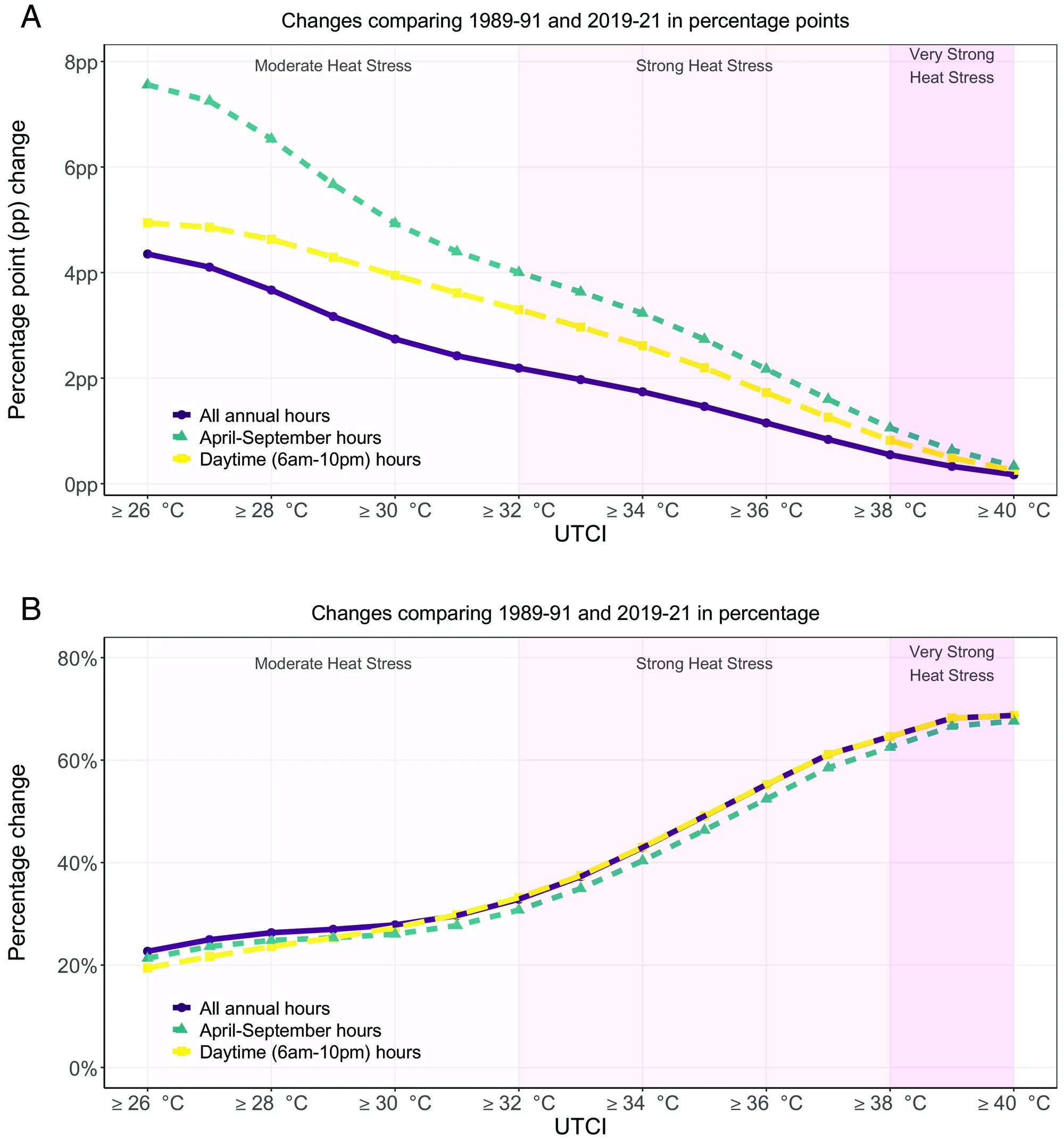
Change in Share of Time for the Average Child at or above UTCI Thresholds for Children 1989–1991 to 2019–2021. Notes: Panel (*A*) displays the percentage points (pp) change in the Share of Time for the Average Child at or above UTCI thresholds comparing 1989–1991 and 2019–2021, while Panel (*B*) displays the percentage changes in this share. The purple solid line represents results for all annual hours, the green short-dash line for the summer season (April to September), and the yellow long-dash line for daytime hours (6 am to 10 pm). Background colors indicate UTCI heat exposure categories. UTCI values above 26 to 32° C indicate moderate heat stress (light pink), from 32 to 38° C indicate strong heat stress (pink), and from 38 to 46 °C indicate very strong heat stress (dark pink). *SI Appendix*, Tables. B.1 and B.2 tabulate results.

Fig. 1 depicts the *percentage-point changes* (Panel *A*) and *percentage changes* (Panel *B*) in the STAC statistics, which measure the average share of time in ambient heat exposure for the average child compare the two reference periods (1989–1991 and 2019–2021). The three-colored backgrounds in this figure reflect the ambient heat exposure levels associated with different UTCI thresholds. UTCI above 26 °C marks the beginning of moderate heat, in which healthy individuals may begin to feel heat discomfort, and vulnerable populations like children may start experiencing elevated health risks, especially with prolonged exposure (51–53). UTCI above 32 °C and above 38 °C mark the onset of strong and very-strong heat, respectively, when health risks become more serious and widespread (51–53). However, our results characterize children’s potential exposure to these ambient heat conditions but do not account for adaptation or directly measure physiological responses or heat-related risks.

We present changes in STAC statistics using three different within-year time frames: all annual hours, daytime hours (6 a.m.–10 p.m.), and hours during April to September, as the hot months of the year. Across the three time-frame specifications, the average child’s share of time under ambient heat exposure increased in China comparing 1989–1991 and 2019–2021 across all UTCI heat thresholds. The largest percentage-point change (Fig. 1*A*) occurred when considering only hours in hot months, followed by daytime hours, and then all annual hours. The percentage changes (Fig. 1*B*) broadly overlapped across the three time-frame specifications.

When all hours were considered, the average child in China experienced 19.2% of total hours in 1989–1991 with ambient heat at-or-above 26 °C UTCI (at-least-moderate heat). By 2019–2021, this percentage increased to 23.5%, representing an approximately 4.4 percentage-point (Fig. 1*A*) and 22.7% increase in annual average exposure duration (Fig. 1*B*). This difference corresponded to an additional 385 h of exposure to at-least-moderate heat in 2019–2021 compared with 1898–1991.

Across all heat intensity thresholds, the average child’s share of time at risk for heat exposure increased. While the percentage-point increases were smaller at higher-heat thresholds than at-least-moderate heat, the percentage increases in the average child shares of time ambiently exposed to UTCI thresholds between 32 °C to 40 °C were at their highest at higher UTCI thresholds. For example, the average duration of children’s ambient exposure to UTCI at-or-above 38 °C (very-strong heat exposure) increased by 0.5 percentage points, reflecting a 64.6% rise as compared to the levels observed in 1989–1991. These results also indicated that approximately 50% [calculated as 1−4.4−2.24.4≈0.50] of the increase in average-heat exposure at the 26 °C threshold can be attributed to the escalation in ambient exposure to strong-or-above heat exposure exceeding the 32 °C threshold. *SI Appendix*, Tables B.1 and B.2 enumerate levels and changes for average child-heat exposure at additional UTCI thresholds.

### Increases in Shares of Children Exposed by Intensity and Duration Threshold (SEIDT).

While the previous results focus on heat exposure for the average child in China, they do not provide information on how many children were increasingly at risk of ambient heat exposure. Therefore, given the changing spatial distributions of heat and of children across counties in China, we computed the SEIDT statistics and examined whether the *percentages* of children most affected by ambient heat exposure also changed over time.

**Fig. 2.**
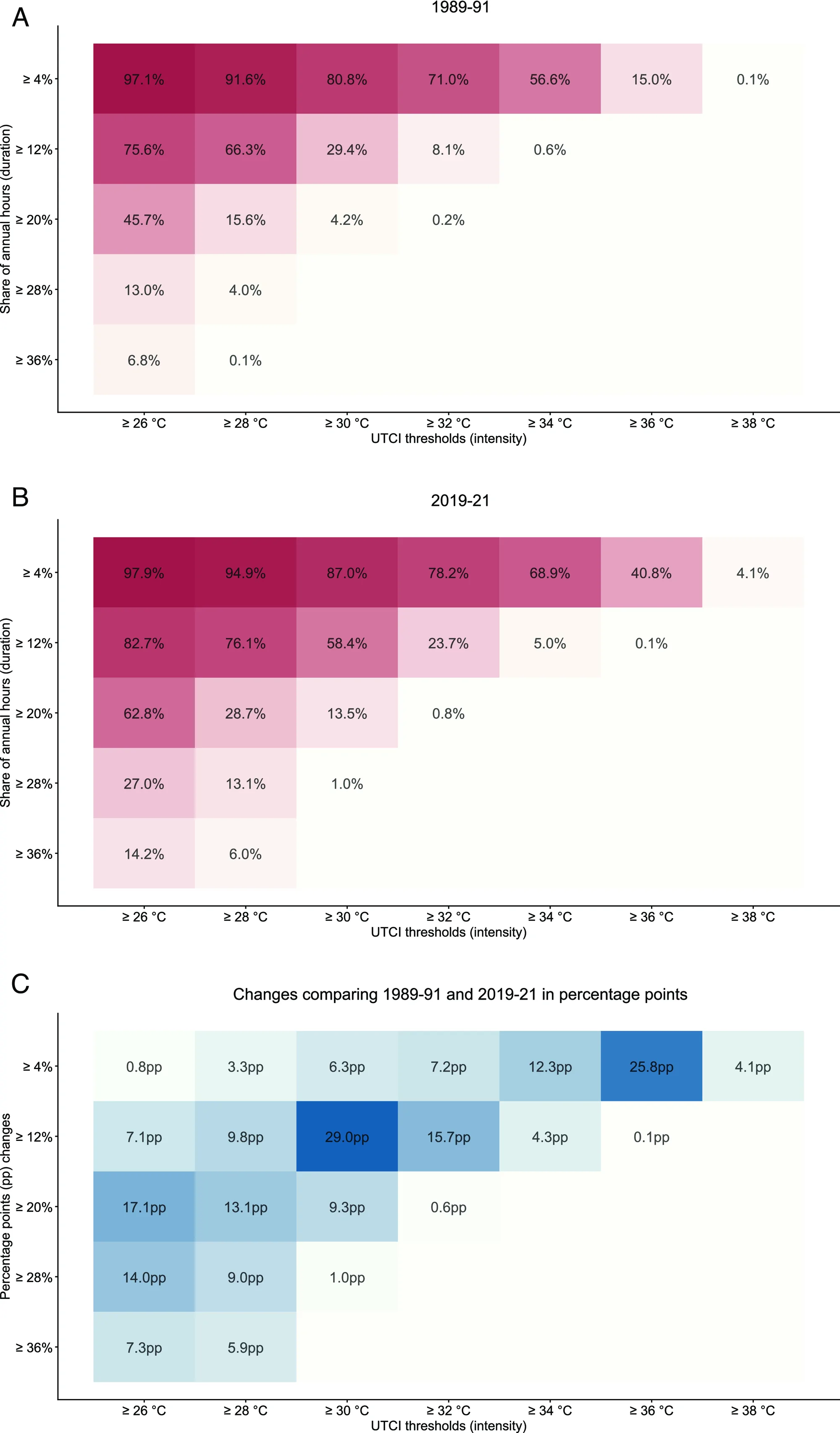
Share of Children Exposed to Heat by Intensity and Duration. Notes: Panel (*A*) presents the share of children at risk of heat exposure by duration and intensity in 1989–1991. The duration is measured as the share of annual hours, whereas the intensity is measured as being at or above the UTCI threshold. Panel (*B*) presents the share of children at risk of heat exposure by duration and intensity in 2019–2021. Panel (*C*) presents the percentage points (pp) changes of children at risk of heat exposure by duration and intensity comparing 1989–1991 and 2019–2021. *SI Appendix*, Tables B.3 and B.4 tabulate results.

We computed the share of children at risk by jointly considering two thresholds: a threshold for the level of ambient heat exposure (intensity) and a threshold for the share of annual hours (duration) exposed to ambient heat corresponding to heat level above a particular threshold. Fig. 2 presents results for combinations of selected heat thresholds for the duration of time exposed (from 4 to 36%) and the intensity of exposure (from above 26 °C to above 38 °C). *SI Appendix* Tables B.3 and B.4 provide tabulations at additional thresholds.

Ambient exposure corresponding to at-least-moderate heat was nearly universal among children in China. In 1989–1991 (Fig. 2*A*) and 2019–2021 (Fig. 2*B*), respectively, 97.1% and 97.9% of children experienced at least 4% of their hours, or over 2 wk, exposed to moderate-or-above ambient heat (i.e., UTCI ≽ 26 °C). At longer durations of exposure, the shares of affected children were lower.

The shares of children enduring prolonged heat exposure were substantially greater in 2019–2021 than in 1989–1991. In 1989–1991, 6.8% of children (21.0 million) experienced moderate or stronger heat for more than 36% of their time, or equivalently, for over 16 wk per year. By 2019–2021, this share rose to 14.2% (34.7 million), marking an increase of about 7.3 percentage points (Fig. 2*C*) or 107%. In other words, the shares of children experiencing *at-least* moderate heat for *at least* 36% of their total hours in 2019–2021 more than doubled compared to 1989–1991.

Similar increases in the shares of children under ambient heat exposures were observed along a frontier of higher heat intensity and lower duration, or lower heat intensity and higher duration, combinations. For example, in 1989–1991, 8.1% of children (24.9 million) had at least 12% of their time, or 6 wk per year, in strong-or-above heat (UTCI≽ 32 °C). By 2019–2021, this number rose to 23.7% (59.1 million), representing a 15.7 percentage-point or 195.0% increase.

Especially alarming were rapid increases in the shares of children in very strong ambient heat environments, emerging first for low duration of exposure. In particular, the shares of children experiencing at least 4% of their total hours at very-strong heat (UTCI ≽ 38 °C) increased from 0.1% (0.3 million) to 4.1% (10.2 million). While the shares of children exposed to these extreme levels remained relatively small, these increases represented approximately a 41-fold jump in the shares of children at these high-intensity heat exposure levels.

### Decomposing the Contributions of Changes in Climate and Population.

Our decomposition analysis illustrates the extent to which changes in children’s heat exposures over time can be attributed to shifts in the spatial distribution of child population, or are due to changes in spatial distribution of heat driven by meteorological factors. In this calculation, we computed counterfactual STAC statistics after altering one distribution (either children’s population or heat) to 2020 levels while keeping the other constant at 1990 levels, without modeling mechanisms of change.

**Fig. 3.**
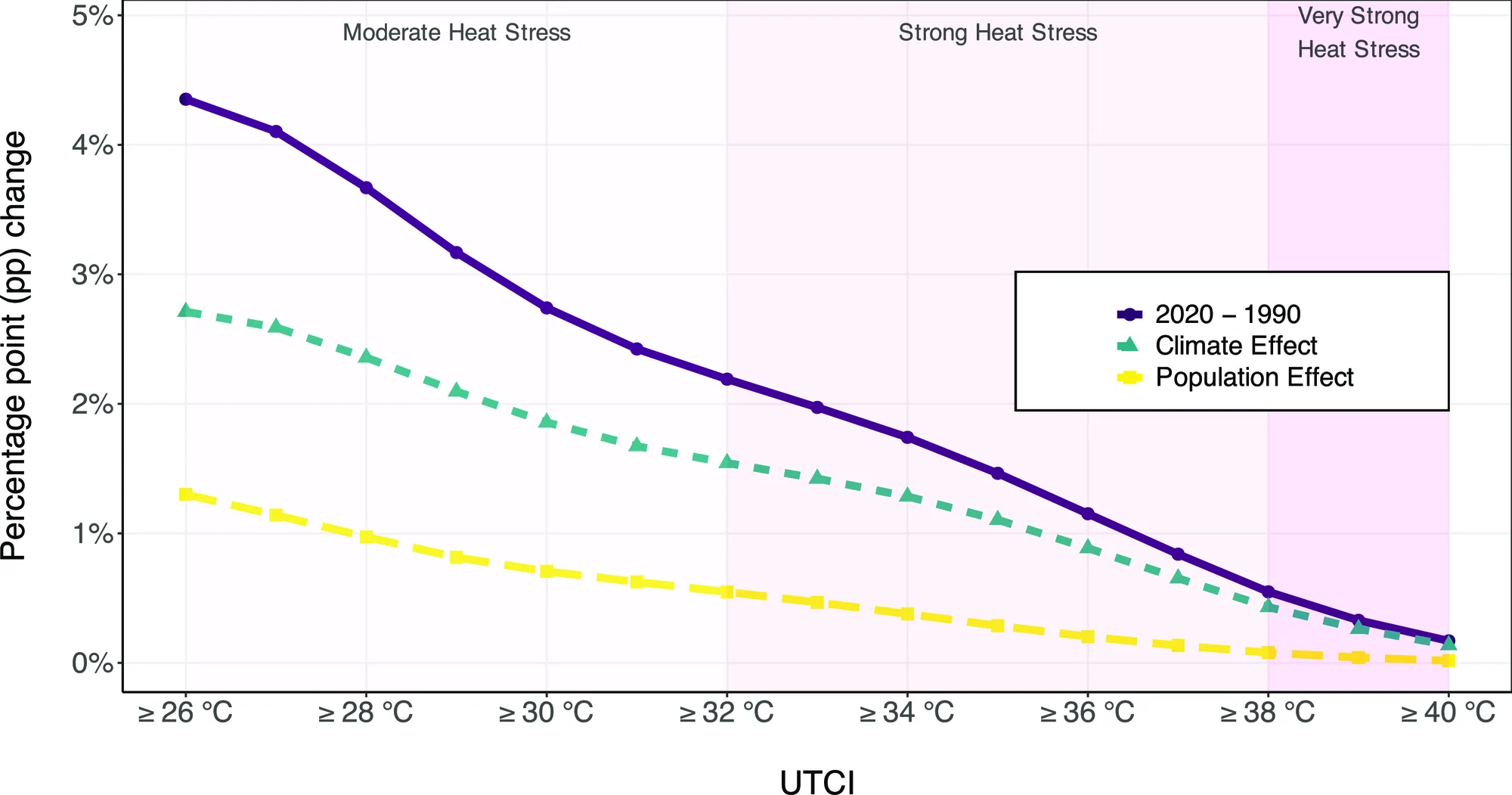
Decomposed Change in Share of Time for the Average Child at Risk of Heat Exposure. Notes: The purple solid line represents the percentage point difference in the share of time for the average child at risk of exposure to heat for children aged 0 to 14 comparing 1990 and 2020 (with 1990 as the baseline year). In the first counterfactual decomposition, we fix the children’s population distribution in 1990 with the observed Universal Thermal Climate Index (UTCI) in 2019–2021. The green short-dash line, or the climate effect, represents the percentage difference between the first counterfactual decom-position results and the baseline (using 1990 children’s population distribution with 1989–1991 UTCI). In the second counterfactual decomposition, we fix the UTCI at the 1989–1991 level and use the children’s population distribution in 2020. The yellow long-dash line, or the population effect, represents the percentage difference between the second counterfactual decomposition results and the baseline. *SI Appendix*, Tables B.5 and B.6 tabulate the results.

Fig. 3 demonstrates that the increase in the average child’s heat exposure between 1990 and 2020 resulted from both climatic change and shifts in children’s population distribution. For heat exposure corresponding to at-least-strong (UTCI ≽ 32 °C) and at-least-moderate (UTCI ≽ 26 °C) levels, child-population-distributional shifts accounted for 30% and 25% of the actual change, respectively. UTCI distributional shifts accounted for 70% and 62% of the actual changes, respectively. The remaining residual changes were due to interactions between shifts in climatic and population distributions.

While both population-distributional changes and climatic changes contributed to the rise in the average-child’s heat exposures, regional decomposition analysis showed varying contributions within regions. Specifically, changes in the population distribution accounted for about 1/3 of the exposure shifts in the Eastern region and less than 1/5 in the Northeastern region. This suggests that cross-region shifts in children’s spatial distribution, for example, due to migration to the Eastern region or fertility decline in the Northeastern region, contributed significantly to the national-population-decomposition results. *SI Appendix*, Table B.5 provides additional details on regional-decomposition results.

Fig. 3 also indicates that at higher heat exposure levels, the influence of changes in population distributions diminished. Nationally, the contribution of population distributions to heat exposure declined with increasing ambient heat corresponding to UTCI thresholds, from 30% at the 26 °C UTCI threshold to 18% at the 36 °C UTCI threshold. Similarly, the contribution of changes in population distributions declined from 30 to 20% in the Eastern region and from 16 to 9% in the Northeastern region over the same sets of UTCI-threshold increments. This suggests that the rise in more-extreme heat exposures was primarily a result of climatic changes, rather than shifts in the geographic distribution of children toward already hotter areas. One possible explanation for this trend is the demographic transition in many highly developed urban areas—often located in hotter coastal regions—where declining fertility rates have led to slower growth or even population declines among children. Further research on regional fertility trends and migration patterns would help clarify whether these demographic shifts contribute to the observed decline in population influence on extreme heat exposures.

### Changes in Children’s Heat Exposure Across Regions.

**Fig. 4.**
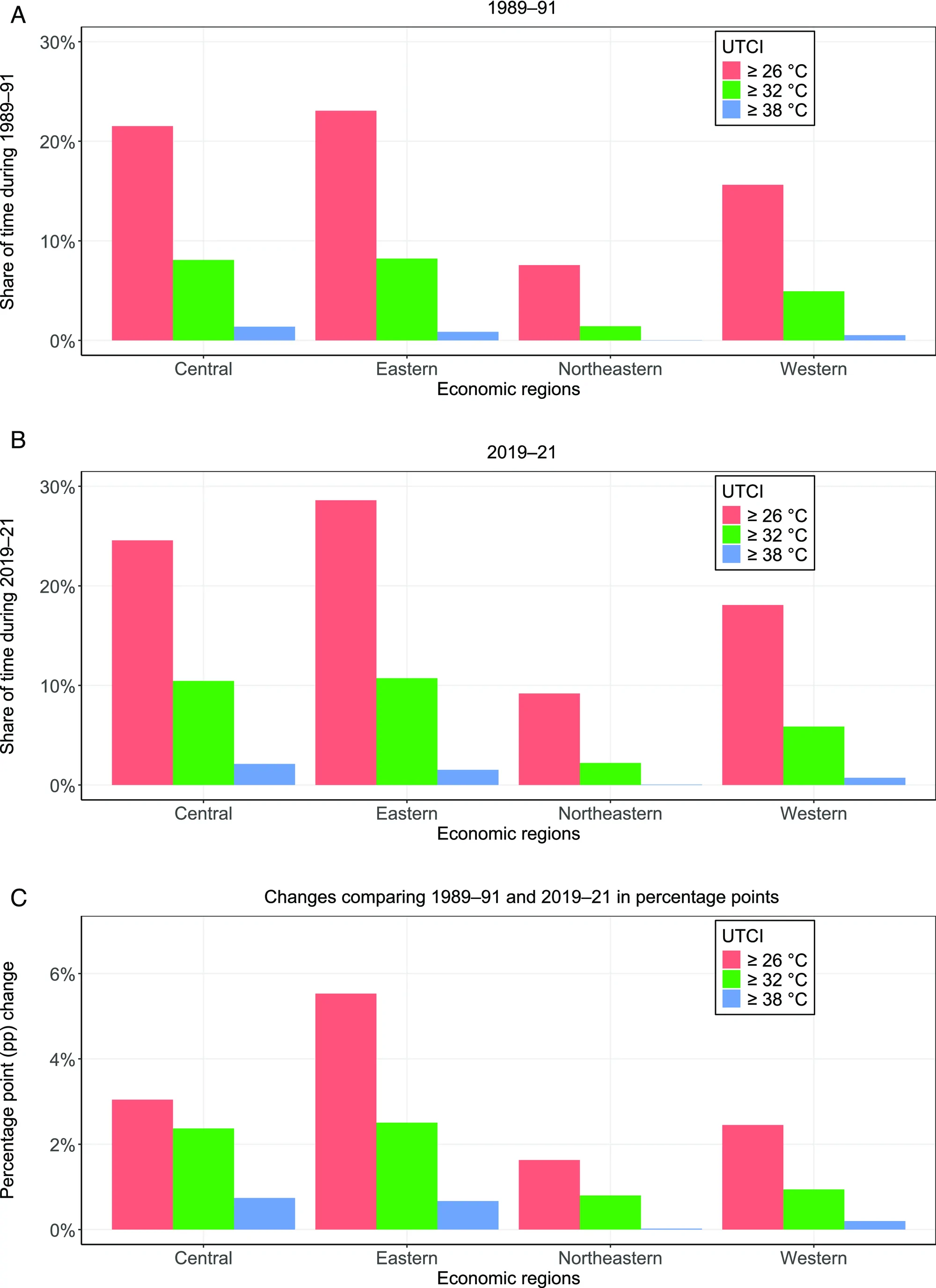
Regional Share of Time for the Average Child at Risk of Heat Exposure. Notes: Panels (*A*) and (*B*) present the regional average shares of annual time at risk of heat exposure for children in 1989–1991 and 2019–2021, respectively. Panel (*C*) presents the percentage-point change comparing 1989–1991 and 2019–2021 across these regions. All panels report results across the four economic regions of China and three heat-exposure thresholds (moderate, strong, and very strong). *SI Appendix*, Tables B.7 and B.8 tabulate results.

We present in Fig. 4 *A* and *B* regional-STAC statistics, which show the average shares of annual time at risk of heat exposure for children in 1989–1991 and 2019–2021 across the four major economic regions of China. Fig. 4*C* shows the percentage-point change comparing 1989–1991 and 2019–2021 across these regions. *SI Appendix*, Tables B.7 and B.8 detail within-region provincial results.

Children in both the Central and Eastern regions experienced high levels of heat exposure. For instance, in 1989–1991, the average child in the Eastern and Central regions faced at-least-moderate heat exposure (UTCI ≽ 26 °C) 23.1% and 21.5% of the time, and at-least-strong heat exposure (UTCI ≽ 32 °C) 8.2% and 8.1% of the time, respectively. The shares of time exposed to at-least-moderate or at-least-strong heat exposure in these two regions remained high in 2019–2021, as shown in Fig. 4*B*.

The percentage-point increases in the shares of time at levels of ambient heat exposure were notable across various heat levels in the Eastern region, with a 5.5 percentage-point increase at the 26 °C UTCI threshold. In contrast, for the Central region, the increases in the shares of time under ambient heat remained at approximately three percentage points for the 26 °C UTCI threshold (Fig. 4*C*). The comparison indicates that although Eastern- and Central-region children both had significant exposures to ambient heat, those in the Eastern region faced a heightened challenge in adapting to ambient heat owing to the rapid increase in average exposure duration.

Compared to the Eastern and Central regions, the Northeastern region had relatively low average-child-heat exposure in 1989–1991. However, the average child’s share of annual time in the Northeastern region increased 22% for at-least-moderate heat exposure (UTCI ≽ 26 °C) and 56% for at-least-strong heat exposure (UTCI ≽ 32 °C). By 2019–2021, the average Northeastern-region child experienced 9.2% and 2.2% of her time under at-least-moderate (UTCI ≽ 26 °C) and at-least-strong (UTCI ≽ 32 °C) heat, respectively. While child ambient heat exposure in the Northeastern region remained much lower than that in the Central and Eastern regions, the rapid increases indicated potential challenges in protecting children for a population that is not accustomed to heat.

Zooming in to the provincial level, in 2019–2021, Hainan (Eastern), Guangdong (Eastern), Guangxi (Western), Jiangxi (Central), and Fujian (Eastern) were generally ranked as the top one to five provinces, respectively, in terms of the average shares of child time exposed to high ambient heat. Specifically, in 2019–2021, children in these provinces had on average 19.6%, 16.2%, 14.6%, 13.6%, and 12.0% shares of time exposed to at-least-strong heat (UTCI ≽ 32 °C), which represented respective increases of 22%, 24%, 21%, 27%, and 36% in shares of time exposed compared to 1989–1991. While Northeastern and Eastern provinces generally experienced substantial increases in heat exposure, provinces in the Central and Western regions experienced limited exposure increases or reductions. *SI Appendix*, Tables B.7 and B.8 detail within-region provincial results.

### Robustness.

We conducted two robustness checks for our results. First, we compared our ambient heat exposure statistics, defined as the share of hours exceeding different ambient UTCI thresholds across locations, to alternative heat exposure statistics based on the share of days children are exposed to heat spells, defined as consecutive days during which daily maximum UTCI exceeds a particular heat exposure level. Using three UTCI thresholds (26, 32, and 38 °C UTCI), and heat-spell durations of at least 3 or 5 consecutive days, we find uniformly high correlations between these two measures, ranging from 0.940 to 0.984 across all combinations. These results demonstrate that the share-of-hours-based heat exposure measure effectively captures heat-spell dynamics.

Second, we compared our main results to results based on shorter UTCI time windows. In the main analysis, we matched 2020 population data with UTCI data from 2019–2021 and 1990 population data with UTCI data from 1989–1991. For robustness, we used a stricter temporal alignment by matching 2020 population data with 2020 UTCI data and 1990 population data with 1990 UTCI data, thereby reducing the temporal mismatch between population and climate observations.

We found that while single-year UTCI data broadly follows the increasing trends of heat exposure as that of our current three-year UTCI data main analysis, the single-years of data undercount the ambient heat intensity and duration. Relative to the three-year averages, the 1990 single-year UTCI is slightly higher, while the 2020 UTCI is slightly lower. The change in share of time for the average child at or above different ambient UTCI thresholds starts at a percentage change of about 14% compared to our main results at about 23% (Fig. 1 and *SI Appendix*, Fig. C.2). The share of children exposed to ambient heat by intensity and duration is up to more than 20 percentage points lower using single-year data versus our main results (Fig. 2 and *SI Appendix*, Fig. C.3). In our decom-position, using the single-year UTCI window results in a climate effect that is less than half of the overall effect, while in our main results the climate effect is approximately two-thirds (Fig. 3 and *SI Appendix*, Fig. C.4). In our regional decomposition, our single-year results undercount the percentage point change in ≽ 26 °C by approximately three percentage points in Central China and by lesser amounts in Western and Eastern China. These results support our usage of three-year UTCI data in our main analyses to better capture heat trends across three decades. More details on these robustness checks are shown in *SI Appendix*, section C.

## Discussion

As climatic change intensifies, the distributions of children’s exposure to extreme heat are of critical concern for human development and public health (11–14), but these distributions have received limited attention in past research. This study analyzed children’s exposure to extreme heat in China comparing the 1989–1991 and 2019–2021 periods. Leveraging both geographical and temporal distributions of heat and of children, our study uncovered significant increases in children’s exposures to moderate-or-stronger heat by an average of 385 h comparing 1989–1991 and 2019–2021. The share of children experiencing more than 18 cumulative weeks of such exposure per year more than doubled, from 6.8 to 14.2%, indicating an increasing proportion of children were exposed to prolonged periods of extreme heat.

Our results highlighted the compound effect of rising temperatures and shifts in child-population-geographical distributions, particularly in a large nation like China with population and temperature distribution heterogeneities. Higher ambient heat exposure, especially in the Eastern region, underscored the urgency for targeted interventions. This includes enhancing climate resilience and heat mitigation strategies, especially in urban areas where “heat island” effects may exacerbate high temperatures (54), as found in cities across China (55). Additionally, our findings underscored the importance of considering demographic changes in addition to climatic trends, as population shifts contributed significantly to the observed increase in heat exposure (33, 39).

Conceptually, our approach to analyzing population-climatic exposure changes moved from the conventional metric of total person-time for measuring overall heat exposure burdens to a *double-dual-distributional* framework that considered both exposure intensity and exposure duration given changing population and climatic risk distributions. This approach enabled a comprehensive analysis of the shares of children experiencing 1) varying degrees of heat (exposure intensity) over 2) varying spans of time (exposure duration). It allowed for cross-time and cross-location comparisons. Moreover, this approach emphasized shifts in population-spatial distribution rather than changes in total population numbers, making it particularly valuable in identifying populations at risk of exposure in scenarios with substantial migration.

Our framework combined county-level census data with gridded-climatic data across time and space. We provided a framework for integrating population and climatic data for climatic and social scientists and policymakers interested in examining changing exposures of different populations to climatic (or other) hazards. While the availability of subnational and consistent global population data across long time spans is limited, subnational census data are publicly available for many countries across time and could be merged with publicly available global-temperature and other climatic data to explore changes in population-based climatic exposures over time.

Our study has limitations. First, we examined ambient heat exposure rather than individually experienced exposure. Individual exposure and its potential consequences are influenced by access to air conditioning, housing characteristics, behavioral adaptations, and other adaptive resources (13, 56). At an aggregate level, regions with similar ambient exposures but at different stages of economic development may also differ in their capacity to adapt to these conditions (57). Moreover, our exposure-based measures quantify children’s potential exposure to high temperatures and should not be interpreted as direct measures of physiological heat stress or heat-related risks. Future research could integrate individual-level survey data containing health outcome measures to better understand how ambient heat exposure translates into physiological heat stress and health outcomes across populations, as well as heterogeneity in adaptation to ambient heat exposure. Second, our analysis overlays UTCI data at a 0.25° × 0.25° spatial resolution onto counties as the basic unit for integrating child population and heat exposure statistics. Although this approach provides a consistent spatial framework for analysis at a finer administrative scale than provinces or prefectures (see *SI Appendix*, section A.2.2 for a detailed discussion), it may obscure finer-scale variations in heat exposure within counties, particularly in densely populated urban areas where microclimatic differences are significant (58, 59). Future research can build on our framework by incorporating finer heat exposure metrics and geographic units to better characterize local patterns of heat exposure. Third, our analyses utilized temporal variations in cross-sectional data. While this approach enabled us to identify changes over time, it did not permit incorporating some important dynamics, such as the relations between climatic conditions and migration (60). Future work using longitudinal data could enrich the analyses by incorporating these dynamics.

Despite these limitations, our study contributed insights about the extent of ambient heat exposure change for children comparing the 1989–1991 and 2019–2021 periods, in the country that had the world’s largest population of children in 1990. Importantly, we showed that changes in population distributions on the national level, though with variations among regions, were about half as important as climatic changes. That the changing spatial distribution of children is a crucial component of changing child heat exposure is potentially relevant globally, given substantial shifts in recent decades in the distribution of the global child population across world regions (45). The simple approach that we developed, moreover, permits insights with minimal data demands and therefore could be easily applied to other countries and regions.

## Materials and Methods

In this section, we summarize our *double-dual-distributional* (DDD) framework for measuring child population exposure to ambient heat conditions. Within a given time span in a region, our DDD framework produces two statistics for heat exposure, based on two distribution types and two threshold types. The two distributions are the distribution of location-specific UTCI-based ambient heat conditions and the distribution of location-specific population groups (e.g., children). The two thresholds are UTCI-based ambient heat thresholds capturing exposure intensity and time thresholds capturing exposure duration. The first statistic captures the average level of extreme-heat exposure among children, measured in units of *share of time for the average child* (STAC) exposed to extreme heat in a particular period. The second statistic captures the distribution of extreme-heat exposure among children, measured in units of *share of the child population exposed by intensity and duration thresholds* (SEIDT). While the overall framework allows for the incorporation of additional heat exposure dimensions—such as the length of specific heat spells—that would require dividing children into more granular cells of exposure experiences. Therefore, we focused on heat intensity and overall time duration as two first-order dimensions of heat exposure experiences.

Existing studies that consider population heat exposures have computed changes in heat exposure in total person-time units for a particular region or country. The person-days of heat exposure in a place at time t can be computed, for example, by multiplying the days during which the maximum temperature exceeds a threshold level with the total population residing in a place at time t. Aggregate person-time statistics have two limitations. First, when comparing exposures over time, aggregate person-time statistics capture changes in aggregate population size over time in addition to changes in average heat exposure burdens. As countries have experienced diverse patterns of changing (both declining and increasing) child populations in recent decades (61), the resultant person-time statistics may not reflect average child heat exposure experiences. Second, the person-time aggregate provides a single statistic of exposure for a region or country, overlooking the within-region or within-country heterogeneities in ambient exposure changes across populations residing in locations with differing climatic-change experiences. In addition to considering both climatic and population distributions, as done in person-time statistics, our DDD framework captures heterogeneities across time and space by measuring changes in the percentages of children experiencing different intensities of associated heat for different durations over time.

Our framework computes population-level distributional changes in heat exposure for children over time. By measuring, in a large economy, changes in the shares of children’s heat exposure based on different intensity and duration thresholds, our framework and empirical results complement and extend existing research that has shown negative effects of heat exposure on children.

We implemented our framework in the setting of China using data from two periods centered around 1990 and 2020. In the empirical application, we treated each span of time as one year and approximated continuous ambient heat exposure using hourly UTCI. To reduce the influence of potential outliers, our main analyses used three-year UTCI averages (e.g., 1989–1991 for 1990 and 2019–2021 for 2020), while results based on single-year UTCI measures are reported as a robustness check. We approximated continuous ambient heat exposures based on hourly UTCI, and we approximated fine-grained measures of locations with counties (3rd-level administrative units) in China. Depending on the analysis, we consider all county-level administrative units in China, in a region of China, or in a province, and generate year-specific child-population-weighted heat distributions by sorting counties along increasing levels of heat exposures and summing up the share of child population along this ascending heat gradient. We show in the *SI Appendix*, section A.2.2 that population data from more than 2,300 Chinese county-level administrative units and heat data from more than 15,000 gridded UTCI data points that overlap with mainland China provide granular joint measurements of child population distribution and heat exposure. For the exposure-intensity thresholds, we considered a range of UTCI thresholds but focused our analysis on key thresholds for extreme heat commonly used in the literature. For exposure-duration thresholds, we considered different shares of time during the course of a year that a child is exposed to UTCI temperatures above the intensity thresholds considered. Our method is also straightforward to implement in other settings where tabular population data at a relatively fine-grained level and location-specific climate data are available.

## Supplementary Material

Appendix 01 (PDF)

## Data Availability

The UTCI data (47) used in this study are publicly available from fifth generation of the European Centre for Medium-Range Weather Forecasts (ECMWF) atmospheric reanalyses of the global climate: the ERA5-HEAT dataset. County-level census data and GIS boundaries were obtained from the the NYU spatial data repository (48) and GitHub (62). Our repository provides processed census data, code for downloading and linking UTCI data, and code and outputs for reproducing the analyses (https://github.com/ClimateInequality/PrjCEC) (63).
