# Supplementary material for: Rising temperatures, shifting population: Changes in children’s ambient heat exposure in China
Source: Proc Natl Acad Sci U S A. 2026 Nov 2;123(45):e2615409123. doi: 10.1073/pnas.2615409123 (PMC13751660; doi:10.1073/pnas.2615409123)
Supplement: Supplementary file 1 [file pnas.2615409123.sapp.pdf]

Supplementary Materials for Rising Temperatures, Shifting Population: Changes in Children's Ambient Heat Exposure in China

A Materials and Methods

A.1 Methods

In this section, we summarize our *double-dual-distributional* (DDD) framework for measuring child population exposed to heat conditions. Within a given time span in a region, our DDD framework produces two statistics for heat exposure, based on two distribution types and two threshold types. The two distributions are the distribution of location-specific UTCI-based ambient-heat conditions and the distribution of location-specific population groups (e.g., children). The two thresholds are UTCI-based ambient heat thresholds capturing exposure intensity and time thresholds capturing exposure duration. The first statistic captures the average level of extreme-heat exposure among children, measured in units of *share of time for the average child* (STAC) exposed to extreme heat in a particular period. The second statistic captures the distribution of extreme-heat exposure among children, measured in units of *share of the child population exposed by intensity and duration thresholds* (SEIDT). While the overall framework allows for the incorporation of additional heat-exposure dimensions—such as the length of specific heat spells—that would require dividing children into more granular cells of exposure experiences. Therefore, we focused on heat-intensity and overall-time-duration as two first-order dimensions of heat-exposure experiences.

Existing studies that consider population heat exposures have computed changes in heat exposure in total person-time units for a particular region or country. The person-days of heat exposure in a place at time t can be computed, for example, by multiplying the days during which the maximum temperature exceeds a threshold level with the total population residing in a place at time t. Aggregate person-time statistics have two limitations. First, when comparing exposures over time, aggregate

person-time statistics capture changes in aggregate population size over time in addition to changes in average heat-exposure burdens. As countries have experienced diverse patterns of changing (both declining and increasing) child populations in recent decades [1], the resultant person-time estimates may not reflect average child heat-exposure experiences. Second, the person-time aggregate provides a single statistic of exposure for a region or country, overlooking the within-region or within-country heterogeneities in ambient exposure changes across populations residing in locations with differing climatic-change experiences. In addition to considering both climatic and population distributions, as done in person-time statistics, our DDD framework captures heterogeneities across time and space by measuring changes in the percentages of children experiencing different intensities of associated heat for different durations over time.

Our framework is the first to compute population-level distributional changes in heat exposure for children over time. By providing—for the first time and in a large economy—measurements of changes in the shares of children's heat exposure based on different intensity and duration thresholds, our framework and empirical results complement and extend existing research that has shown negative effects of heat exposure on children.

We implemented our framework in the setting of China using data from two periods centered around 1990 and 2020. In the empirical application, we treated each span of time as one year and approximated continuous ambient heat exposure using hourly UTCI. To reduce the influence of potential outliers, our main analyses used three-year UTCI averages (e.g., 1989–1991 for 1990 and 2019–2021 for 2020), while results based on single-year UTCI measures are reported as a robustness check. We approximated continuous-ambient-heat exposures based on hourly UTCI, and we approximated fine-grained measures of locations with counties (3rd-level administrative units) in China. Depending on the analysis, we consider all county-level administrative units in China, in a region of China, or in a province, and generate year-specific child-population-weighted heat distributions by sorting counties along increasing lev-

els of heat exposures and summing up the share of child population along this ascending heat gradient. We show in the Supplement Section A.2.2 that population data from more than 2,300 Chinese county-level administrative units and heat data from more than 15 thousand gridded UTCI data points that overlap with mainland China provide granular joint measurements of child population distribution and heat exposure. For the exposure-intensity thresholds, we considered a range of UTCI thresholds but focused our analysis on key thresholds for extreme heat commonly used in the literature. For exposure-duration thresholds, we considered different shares of time during the course of a year that a child is exposed to UTCI temperatures above the intensity-thresholds considered. Our method is also straightforward to implement in other settings where tabular population data at a relatively fine-grained level and location-specific climate data are available.

Location- and Period-Specific Heat Exposure We now formalize our heat exposure analysis framework across time and space. Specifically, let $c_l(t)$ be the heat experienced by an individual at a moment in time t at a location l. Between period t and $t + \tau$, the share of time that individuals at location l experience heat $c_l(t)$ over threshold c^* is, $s_l(c^*, t, \tau)$:

$$s_l(c^*, t, \tau) = \frac{1}{\tau} \int_t^{t+\tau} \mathbf{1}\{c_l(t) > c^*\} \, dt \,. \tag{1}$$

Depending on the analysis, our definition of time period includes all time during the day, all day time hours (6 am to 10 pm), or all hours within different seasons (e.g., April–September, October–March). Additionally, let $P_{t \leqslant t < t+\tau}(l|m)$ be the share of population for socio-demographic group m in a location l, among L locations in total between time t and $t + \tau$. Total population shares across locations sum up to 1: $\sum_{l=1}^{L} P_{t \leqslant t < t+\tau}(l|m) = 1$.

Average Share of Time of Heat Exposure We compute two key sets of statistics. First, we compute $\mathcal{S}_m(c^*, t, \tau)$, which measures, during a particular interval of time,

the average share of time individuals of socio-demographic group m are exposed to heat over threshold c^*:

$$\mathcal{S}_m\left(c^*, t, \tau\right) = \sum_{l=1}^{L} P_{t \leqslant t < t+\tau}\left(l|m\right) \cdot s_l\left(c^*, t, \tau\right). \tag{2}$$

$\mathcal{S}_m\left(c^*, t, \tau\right)$ is the *Share of Time for the Average Child* (STAC) statistic, which measures, for the average child, the share of time exposed to ambient extreme heat during a particular time-frame across heat thresholds.

Since $\mathcal{S}_m\left(c^*, t, \tau\right)$ is a statistics for share of time, it varies between 0 and 1. In particular, $\lim_{c^* \to \infty} \mathcal{S}_m\left(c^*, t, \tau\right) = 0$ and $\lim_{c^* \to -\infty} \mathcal{S}_m\left(c^*, t, \tau\right) = 1$. A key aggregate statistic for how heat exposure shifts between period t' and t is the following difference:

$$\Delta\mathcal{S}_{m,t',t}\left(c^*, \tau\right) = \mathcal{S}_m\left(c^*, t', \tau\right) - \mathcal{S}_m\left(c^*, t, \tau\right). \tag{3}$$

$\Delta\mathcal{S}_{m,t',t}\left(c^*, \tau\right)$ is the population-weighted average increase in the share of time exposed to the potential key heat threshold c^* between time t and t' for population group m. $\Delta\mathcal{S}_{m,t',t}\left(c^*, \tau\right)$ shifts due to both shifts in the population distribution as well as the distribution of heat between t and t', thus taking into account both population and meteorological changes across time and space.

Share of Children by Duration and Intensity of Heat Exposure Second, we compute the share of individuals at risk, based on a joint consideration of the relevant heat threshold that might be considered risky for human development, and the share of time exposed to such heat that would put individuals at risk of non-transitory impacts.

We consider these two joint dimensions of risks in computing population exposure statistics. Specifically, let $s^*\left(\tau\right)$ be a particular share-of-time threshold within span of time τ above a specific heat threshold. We define the m-, c^*-, and s^*-specific at-risk

measure $\mathcal{R}_m(c^*, s^*, t, \tau)$ between time t and $t + \tau$ as:

$$\mathcal{R}_m(c^*, s^*, t, \tau) = \sum_{l=1}^{L} P_{t \leqslant t < t+\tau}(l|m) \cdot \mathbf{1}\{s_l(c^*, t, \tau) > s^*(\tau)\}. \quad (4)$$

$\mathcal{R}_m(c^*, s^*, t, \tau)$ is the *Share Exposed by Intensity and Duration Thresholds* (SEIDT) statistic, which measures the share of children exposed to extreme heat by differing heat (intensity) and time (duration) thresholds. We use UTCI-based heat exposure levels to characterize heat exposure intensity. The Data section provides a more detailed discussion of the UTCI and the classification of heat exposure intensity.

By construction, $\mathcal{R}_m(c^*, s^* = 0, t, \tau) \leqslant 1$ and $\mathcal{R}_m(c^*, s^* = 1, t, \tau) = 0$. Additionally, the share of individuals experiencing greater than s^* share of time over c^* threshold converges to 0 as c^* increases: $\lim_{c^* \to \infty} \mathcal{R}_m(c^*, s^*, t, \tau) = 0$.

For the socio-demographic group indexed by m, given heat threshold c^* and share of time threshold s^*, the percentage increase over time in the share of individuals from this group at risk of excess heat exposure is:

$$\Delta\mathcal{R}_{m,t',t}(c^*, s^*, \tau) = \mathcal{R}_m(c^*, s^*, t', \tau) - \mathcal{R}_m(c^*, s^*, t, \tau). \quad (5)$$

One important aspect of our framework is that computing $\mathcal{R}_m(c^*, s^*, t, \tau)$ and $\Delta\mathcal{R}_{m,t',t}(c^*, s^*, \tau)$ do not require the use of harmonized geographic data overtime. This is often a constraint in the analysis of heat or other climate risk changes over time, due to shifting administrative boundaries, especially across large spans of time. In our framework, we consider all $l \in \{1, \ldots, L\}$ locations within a region, and generate time-specific population-heat cumulative distribution functions by sorting locations along the gradient of heat exposures and summing up the share of population for socio-demographic group m along ascending levels of heat exposures. While our distributions are discretized by location-level administrative units, when there are large number of locations with dispersed population, the population-heat distributions tend to be approximately smooth. Cross-time comparisons, especially at higher levels of regional aggregation, are based on these approximately smooth

distributions over time. Hence, moments and percentiles of these population-heat distributions are robust to shifts in sub-region location boundaries.

Framework and Empirical Results In our empirical application, t is 1990 and, t' is 2020, τ is one calendar year, and m is children between ages 0 and 14. Additionally, we approximate continuous time with hourly measurements. As an example, $\Delta\mathcal{R}_{\text{children},2020,1990}$ with $c^* = 28$ and $s^* = 0.1$ provides the change in the percentage points of children exposed to heat over 28 degrees for greater than 10 percent of their time during a year.

Results for $\Delta\mathcal{S}_{m=\text{ages 0 to 15},t'=\text{year 2020},t=\text{year 1990}}(c^*, \tau = 1 \text{ year})$ are summarized in Figure 1 and Tables B.1 and B.2. These capture changes in the average share of time children are exposed to heat over a range of heat thresholds—$23°C \leqslant c^* \leqslant 40°C$—considering all hours, daytime hours, or hours during hotter and colder seasons.

Results for $\Delta\mathcal{R}_{m=\text{ages 0 to 15},t'=\text{year 2020},t=\text{year 1990}}(c^*, s^*, \tau = 1 \text{ year})$ are summarized in Figure 2 and Tables B.3 and B.4. These capture changes in the share of children experiencing ambient heat exposure for over a range of c^* heat *intensity* thresholds—$23°C \leqslant c^* \leqslant 40°C$—and s^* heat exposure *duration* thresholds—$4\% \text{ of year} \leqslant s^* \leqslant 36\% \text{ of year}$.

Additionally, Figure 3 and Tables B.5 and B.6 summarize decompositional results that compute changes in the average share of time of child exposure, but combining 1990 population distribution with 2020 heat exposure distribution, and also 1990 heat exposure distribution with 2020 population distribution. Finally, Figure 4 and Tables B.7 and B.8 provide regional results on the changes in the average share of time of child exposure.

A.2 Data

A.2.1 Population and Heat Data Sources

ERA5 Data Details To measure heat, we used the fifth generation of the European Centre for Medium-Range Weather Forecasts (ECMWF) atmospheric reanalyses of the

global climate: the ERA5-HEAT dataset [2, 3]. Covering the period from 1940 to the present, ERA5-HEAT comprises hourly gridded maps of the Universal Thermal Climate Index (UTCI) at 0.25° × 0.25° spatial resolution. The dataset is publicly accessible through the Copernicus Climate Change Service's Climate Data Store (CDS). The UTCI is a widely used index to assess the human-perceived thermal stress based on atmospheric conditions, integrating atmospheric parameters like temperature, humidity, wind speed, and solar radiation. UTCI is expressed in degrees Celsius (°C), and it provides a measure of how cold or hot people might feel under prevailing environmental conditions [4, 5, 6, 7]. When UTCI is between 26 °C and 32 °C, it indicates moderate-heat stress on the human body, signifying warm conditions where individuals may start to feel uncomfortable, especially if engaging in physical activity. As the UTCI value increases, the level of thermal stress on the human body intensifies. UTCI values between 32 °C and 38 °C indicate strong-heat stress, whereas UTCI values between 38 °C and 46 °C indicate very-strong-heat stress [4]. We utilized these UTCI stress categories in this study. In the main text, we refer to these UTCI-based heat stress categories as "heat exposure." However, our exposure-based measures quantify children's potential exposure to heat and should not be interpreted as direct measures of physiological heat stress or heat-related health risk.

Census Population Data Details For population data, we utilized Chinese-census data for the years 1990 and 2020 [8, 9, 10, 11]. County-level child population data and county-level administrative boundary files were extracted and used to construct ages 0–14 populations by county. For regional analysis, we considered the child population and UTCI distributions within each one of the four recognized economic regions of China [12].

We use information from 2,369 geographical units at the county level nested in 31 provincial administrative units from the Tabulation on 1990 China Population Census by County. We start with the 1990 Chinese Census as it is the first to offer county-level population counts for individuals between ages 0 to 14. We only include mainland

China and do not include special administrative regions. Within each county, we calculate the sum of ages 0 to 14 child population regardless of gender. In 1990, the total number of children across all counties included for analysis was 312,995,886.

We use information from 2,853 geographical units at the county level nested in 31 provincial administrative units from the Tabulation on 2020 China Population Census by County. We only include mainland China and do not include special administrative regions. We again calculate the population counts for children between ages 0 to 14, regardless of gender, in our analysis. In 2020, the total number of children across all counties included for analysis was 249,260,992.

A.2.2 Child Population-weighted Heat Distribution

Depending on our analysis, we consider all county-level administrative units in China, in a region, or in a province, and generate year-specific child-population-weighted (for children ages 0–14) UTCI cumulative distribution by sorting counties along ascending levels of heat exposure and summing up the share of child population county-by-county along the ascending levels of heat exposure.

In this section, we show the population- and spatial-granularity of our county-based analysis and find that counties provide a granular unit for child population and heat measurement in the Chinese and UTCI data setting. We use population data from 2369 (in 1990) and 2853 (in 2020) county-level administrative units and heat data from 15,432 gridded UTCI data points that cover mainland China. We find that child population is widely dispersed across counties (see Table A.1), and this leads to child-population-weighted heat distributions that are approximately smooth and do not contain large discretized "steps." Additionally, we find that most Chinese counties, with the exception of large western counties with small child population levels, have limited geographical expanse and hence limited number of within-county UTCI measurements (see Table A.2). This means that county mean heat levels summarize well the UTCI-based ambient heat exposure measurements within counties.

It is important to note that the geography of county-level administrative boundaries

shifted significantly between 1990 and 2020. But our child-population based analysis does not compare each county-level unit over time, instead, we compare the year-specific national and regional fine-grained child-population-weighted heat distributions using cross-county information.

The Distribution of Children Across Counties In Table A.1, we order county-level administrative units by their child population counts, and construct county-level national and regional child-population distributions. Panel A shows national distributions while Panels B through E show results from regional distributions. Overall, the results show that county-level administrative units provide a granular unit for constructing child-population-weighted statistics in the Chinese setting.

Nationally, we find that child population is widely dispersed across counties. The median Chinese county-level administrative unit accounts for 0.057% (186 thousand children) of the national child population in 1990 and 0.051% (128 thousand children) in 2020, while the county at the 99th percentile of child-population distribution accounts for 0.382% (1.240 million children) in 1990 and 0.276% (690 thousand children) of the national child population in 2020.

Regionally, child population is also widely dispersed across counties, with some variations over time. We see that in 1990, the median county in Western China accounts for 0.042% (137 thousand children) of the national child population, about four-fifths of the median share in Northeastern China (0.051%, 165 thousand children) and two-thirds of the median share in Eastern (0.063%, 206 thousand children) and Central (0.066%, 216 thousand children) China. Due to an increase in the number of counties per region and an overall decrease in total child population, in 2020, the share and number of children in Chinese counties decreased compared to 1990, across regions. Specifically, in 2020, the median county in Western, Northeastern, Eastern, and Central China accounts for 0.040% (99 thousand children), 0.021% (52 thousand children), 0.060% (151 thousand children), and 0.057% (143 thousand children) of the national child population in China. The largest counties in terms of child population appear

in Eastern China and also only account for a small fraction of the national population: In 1990 and 2020, the county at the 99th percentile of Eastern China's child population distribution accounts for 0.448% (1.453 million) and 0.551% (1.376 million) of the national child population, respectively.

The Distribution of Grid Points Across Counties In Table A.2, we present the distribution of UTCI grid points across counties and the distribution of children across counties in 1990 and 2020. We first show the number of grid points in column one, followed by the number of counties across 1990 and 2020 matched to different numbers of overlapping grid points, with the fourth and fifth columns depicting the percentage of children within those counties. The remaining columns depict the row-cumulative percentage of children.

We find that most Chinese counties have limited geographic expanse, and this allows the matching of granular child population information to granular gridded heat measurements. More specifically, in 1990, 41% of counties (970 counties) intersect with two or fewer UTCI spatial grid points, and these counties account for 41% of the total child population. Additionally, 77% of counties (1,826 counties) intersect with five or fewer UTCI spatial grid points, and these counties account for 83% of the total child population. In 2020, 51% of counties (1,465 counties) intersect with two or fewer UTCI spatial grid points, and these counties account for 55% of the total child population. Additionally, 81% of counties (2,323 counties) intersect with five or fewer UTCI spatial grid points, and these counties account for 89% of the total child population. Overall, in both 1990 and 2020, more than 95% of children reside in counties that are associated with ten or fewer grid points. Using information for the city of Shanghai and Henan Province, Figure A.1 and Figure A.2 provide illustrative examples of the overlap between county-level administrative boundaries and UTCI grid points, respectively.

Some county-level administrative units, in particular from Western China, are geographically expansive, and pose a challenge to our county-based analysis. Specifically, 39 counties in 1990 and 41 counties in 1990 overlapped with between 51 and

330 gridded UTCI points (see Table A.2). Figure A.3 illustrates this with the example of Western China's Qinghai province, which has several geographically large county-level administrative areas toward the western side of the province. Heat levels can vary substantially across these gridded points and we do not have disaggregated child population data within these counties. However, the share of child population in these counties is only about 0.1% of the national population, and aggregation errors in these areas do not measurably impact national and regional child-population weighted distributional statistics.

Figure A.1: Chinese Counties and UTCI Grids, Shanghai

(a) 1990 districts/counties

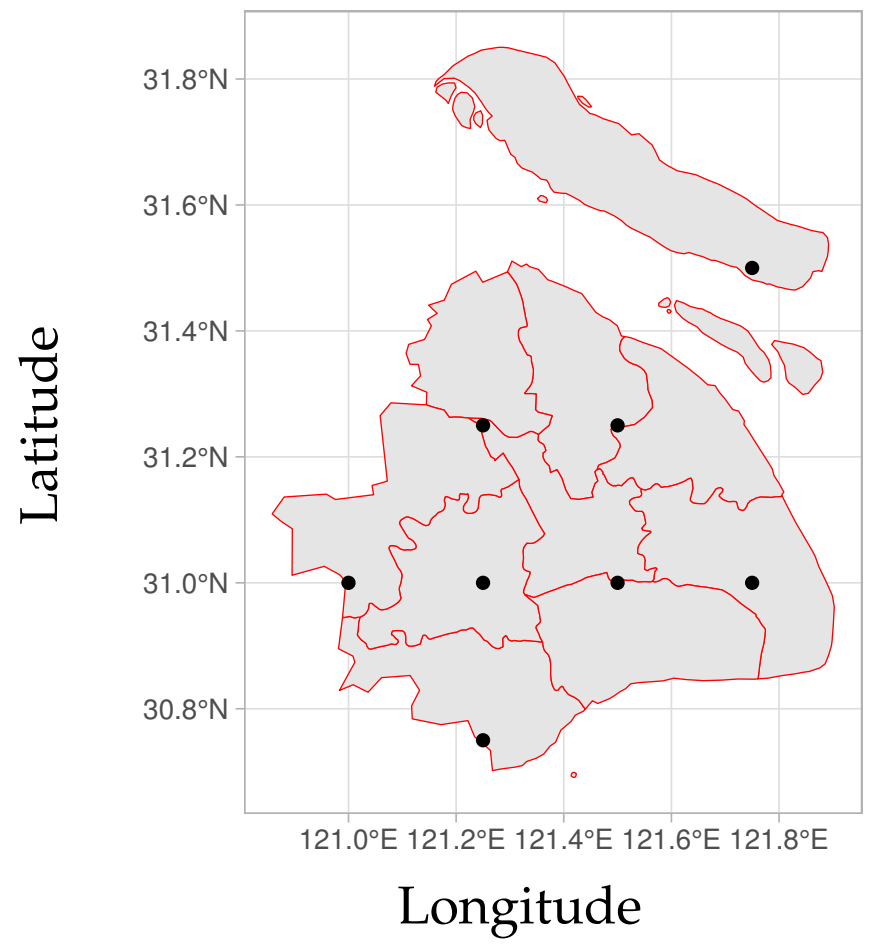

(b) 2020 districts/counties

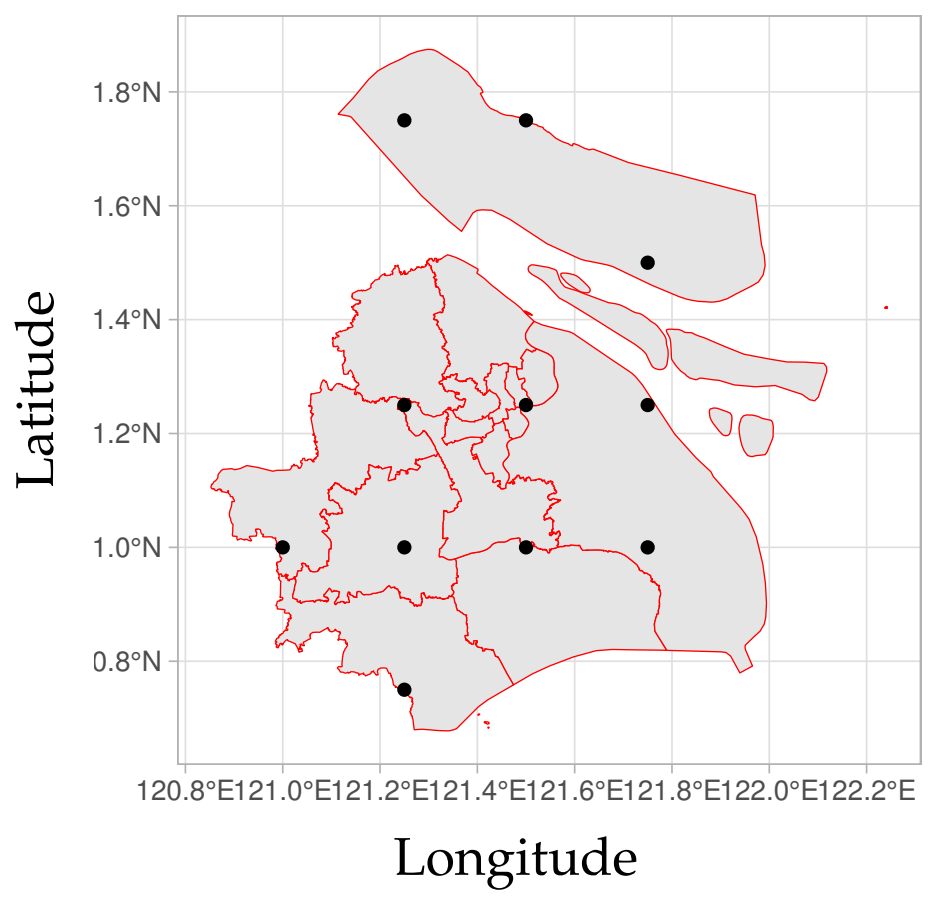

Notes: We superimpose UTCI grid points (0.25° × 0.25° longitude–latitude grid) over the 1990 and 2020 district/county-level administrative boundaries (red boundary lines) from Shanghai, China. We show grid points (shown as black dots) that fall within the boundary of at least one district/county-level administrative unit. We associate county-level child population data with the average hourly temperature of grid points that fall within the boundaries of the county-level administrative unit. For Shanghai counties that do not overlap with any grid points, we associate children in the county with the hourly UTCI of the spatial grid point closest to the county's centroid.

Figure A.2: Chinese Counties and UTCI Grids, Henan

(a) 1990 counties

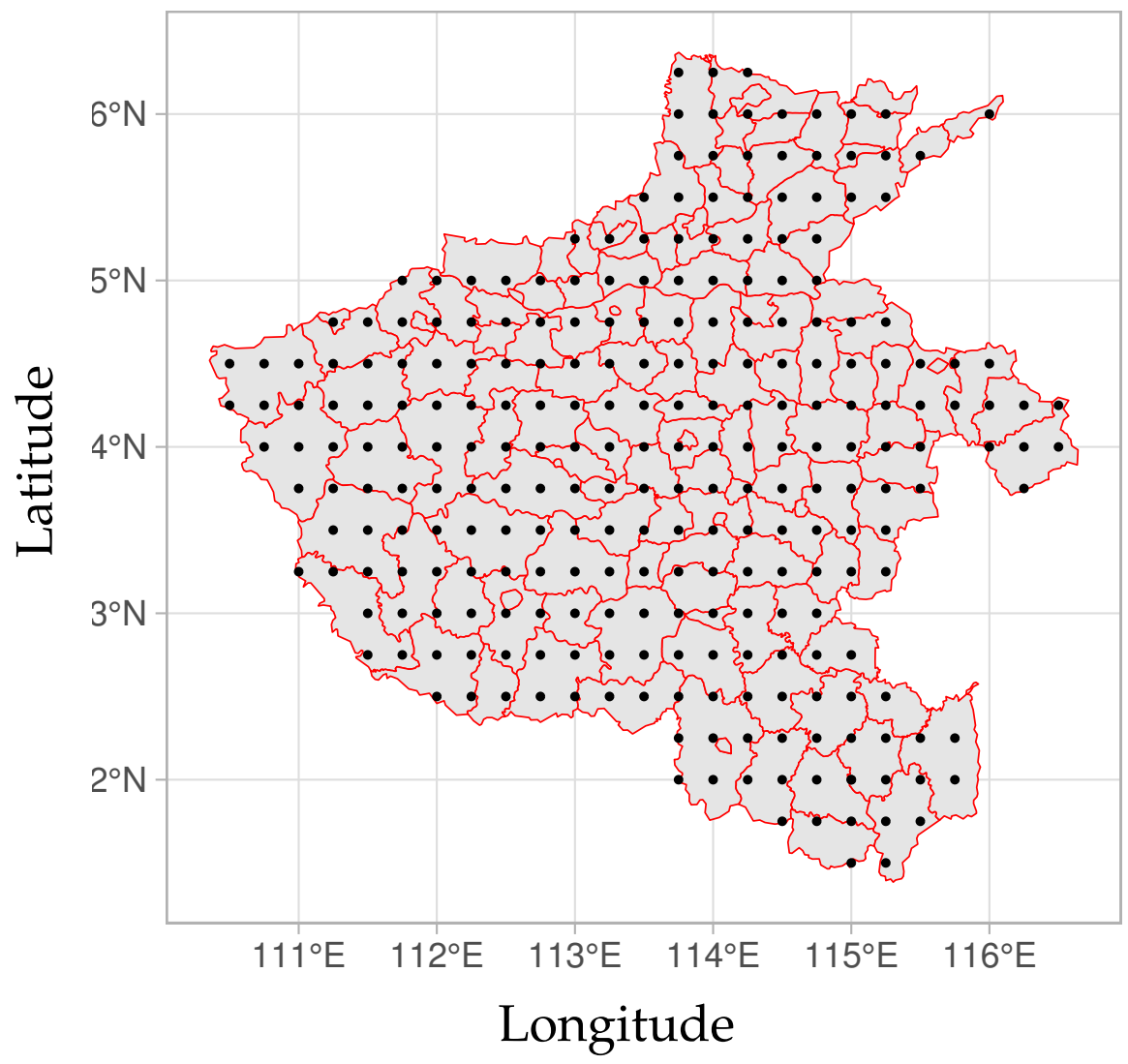

(b) 2020 counties

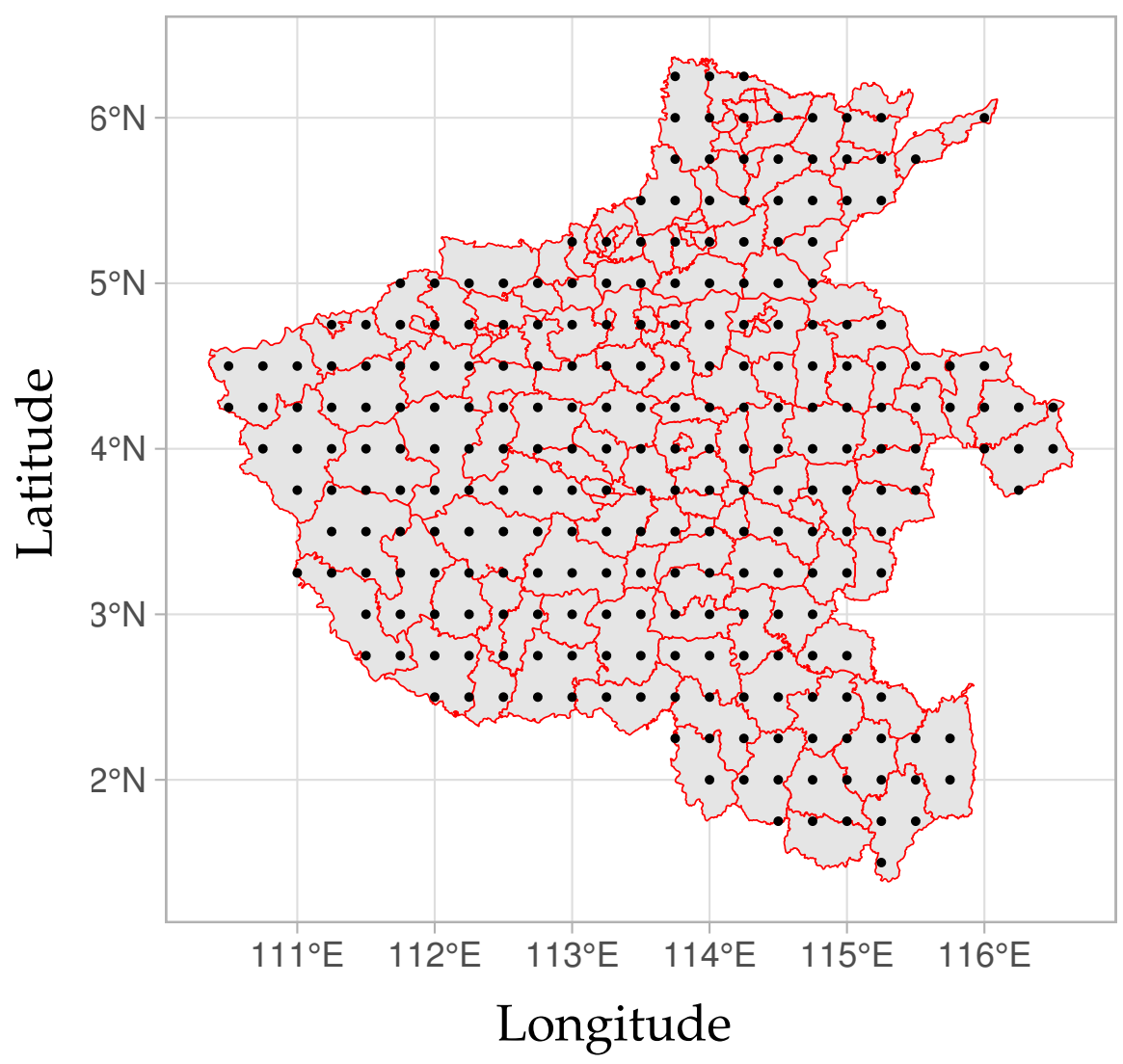

Notes: We superimpose UTCI grid points ($0.25° \times 0.25°$ longitude–latitude grid) over the 1990 and 2020 county-level administrative boundaries (red boundary lines) from Henan, China. We show grid points (shown as black dots) that fall within the boundary of at least one county-level administrative unit. We associate county-level child population data with the average hourly temperature of grid points that fall within the boundaries of the county-level administrative unit.

Figure A.3: Chinese Counties and UTCI Grids, Qinghai

(a) 1990 counties

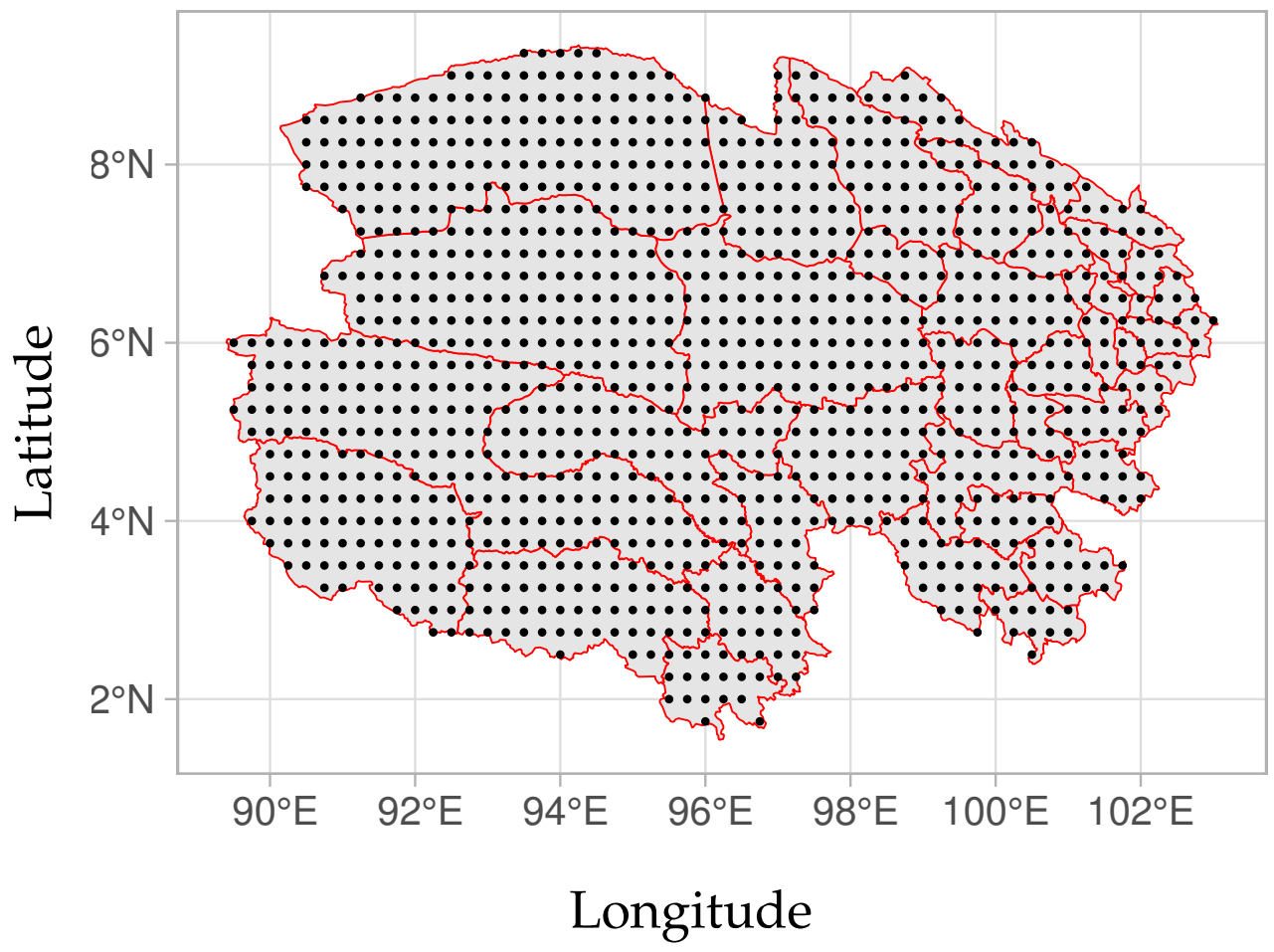

(b) 2020 counties

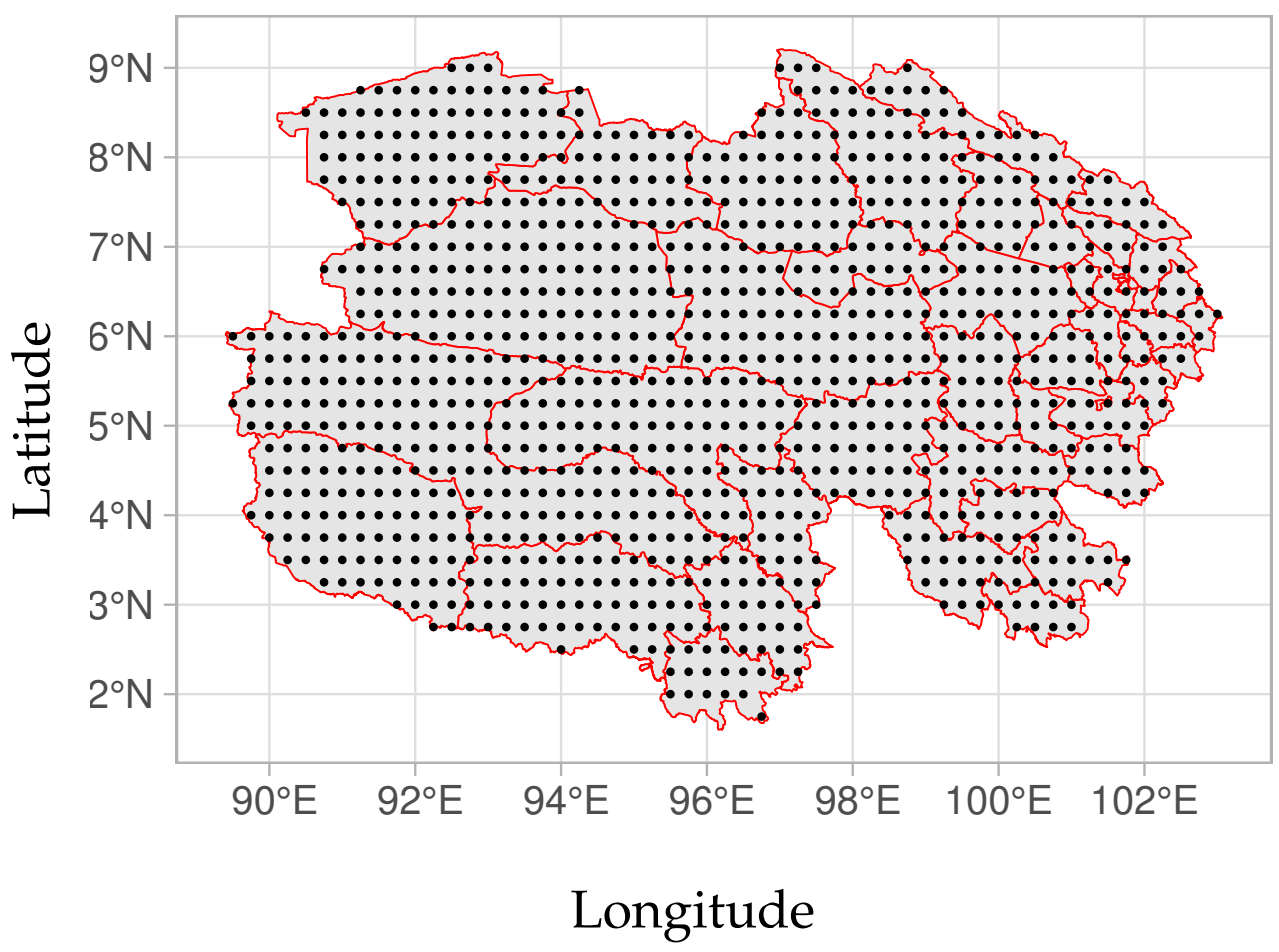

Notes: We superimpose UTCI grid points (0.25° × 0.25° longitude–latitude grid) over the 1990 and 2020 county-level administrative boundaries (red boundary lines) from Qinghai, China. We show grid points (shown as black dots) that fall within the boundary of at least one county-level administrative unit. We associate county-level child population data with the average hourly temperature of grid points that fall within the boundaries of the county-level administrative unit.

Table A.1: The Distribution of Chinese Children (ages 0-14) Across Counties in 1990 and 2020

		Percentiles						
Statistics	Year	1	10	25	50	75	90	99
**Panel A: National**								
Number	1990	27.5K	79.3K	117.0K	185.7K	291.0K	405.5K	1240.1K
Percent	1990	0.008%	0.024%	0.036%	0.057%	0.09%	0.125%	0.382%
Number	2020	15.5K	47.9K	77.4K	128.3K	201.7K	296.0K	689.6K
Percent	2020	0.006%	0.019%	0.031%	0.051%	0.081%	0.118%	0.276%
**Panel B: Central region**								
Number	1990	39.6K	89.9K	140.9K	215.7K	312.4K	404.9K	540.0K
Percent	1990	0.012%	0.028%	0.043%	0.066%	0.096%	0.125%	0.166%
Number	2020	20.3K	54.7K	86.7K	142.7K	209.3K	270.6K	374.3K
Percent	2020	0.008%	0.022%	0.035%	0.057%	0.084%	0.108%	0.15%
**Panel C: Eastern region**								
Number	1990	52.2K	94.3K	138.1K	206.0K	320.9K	486.7K	1453.2K
Percent	1990	0.016%	0.029%	0.043%	0.063%	0.099%	0.15%	0.448%
Number	2020	33.3K	67.8K	97.9K	150.8K	239.1K	378.8K	1376.4K
Percent	2020	0.013%	0.027%	0.039%	0.06%	0.096%	0.152%	0.551%
**Panel D: Northeastern region**								
Number	1990	25.2K	83.9K	116.8K	165.4K	249.8K	332.4K	984.1K
Percent	1990	0.008%	0.026%	0.036%	0.051%	0.077%	0.102%	0.303%
Number	2020	6.0K	21.5K	33.4K	51.5K	76.4K	116.9K	233.0K
Percent	2020	0.002%	0.009%	0.013%	0.021%	0.031%	0.047%	0.093%
**Panel E: Western region**								
Number	1990	14.6K	54.9K	90.1K	137.3K	238.4K	350.5K	563.6K
Percent	1990	0.004%	0.017%	0.028%	0.042%	0.073%	0.108%	0.174%
Number	2020	10.6K	37.1K	62.4K	99.4K	164.9K	235.4K	405.0K
Percent	2020	0.004%	0.015%	0.025%	0.04%	0.066%	0.094%	0.162%

Note: Drawing on the 1990 and 2020 Chinese census, we present key percentiles of county-level distributions of the child population (ages 0–14) in China in 1990 and 2020. In the rows where the statistics are labelled "Number", we show the number of children in units of thousands of children. In the rows where the statistics are labelled "Percent", we show the percent of children in a county as a share of the overall child population (ages 0–14) in the country. We present national and regional results. The statistics show, for example, that in 1990, the median county in China had 185.7 thousand children, which is equivalent to 0.057% of the overall child population (ages 0–14) in the country. Across all panels representing regional and national contexts, any one county provides a small amount of the child population data, for example, approximately 0.45% in the 99th percentile of the dense Eastern region. Counties, as a geographic unit, provide a reasonable range and comparable distribution of the child population across China.

Table A.2: The Distribution of the Number of Overlapping Grid Points (0.25° × 0.25° longitude–latitude grid) and the Distribution of Children (ages 0–14) among the 2369 Chinese Counties in 1990 and 2853 Chinese Counties in 2020

Number of grid points	Number of counties		Percentage of children		Cumulative percentage of children	
	1990	2020	1990	2020	1990	2020
**Panel A: Less than 6 grid points falling within a county**						
1	503	957	19.89%	34.51%	19.89%	34.51%
2	467	508	21.29%	20.18%	41.19%	54.68%
3	378	382	17.58%	15.51%	58.77%	70.19%
4	307	309	16.08%	12.94%	74.84%	83.13%
5	171	167	8.10%	6.23%	82.94%	89.36%
**Panel B: Between 6 and 50 grid points falling within a county**						
6 to 10	280	275	12.65%	7.42%	95.59%	96.77%
11 to 20	135	131	2.77%	1.77%	98.36%	98.54%
21 to 30	46	41	0.75%	0.68%	99.11%	99.22%
31 to 40	29	27	0.34%	0.27%	99.46%	99.50%
41 to 50	14	15	0.19%	0.13%	99.65%	99.63%
**Panel C: 51 or more grid points falling within a county**						
51 to 100	24	27	0.25%	0.27%	99.90%	99.89%
101 to 200	12	11	0.09%	0.09%	99.99%	99.99%
201 to 330	3	3	0.01%	0.01%	100.00%	100.00%

Note: We overlay boundaries for Chinese counties (administrative level 3) in 1990 and 2020 with 0.25° × 0.25° longitude–latitude spatial grids, which is the spatial resolution for the Universal Thermal Climate Index (UTCI) data that we use. We count the number of grid points that intersect (fall within the boundary) with each Chinese county. For county boundaries that do not intersect with points on the grid, we associate the county with the spatial grid point closest to the centroid location of the county, and count that county as having 1 grid point. We assign 127 counties from 1990 and 393 counties from 2020 a grid point due to their lack of intersection. In the first column, we present categorizations of counties by the number of 0.25° × 0.25° spatial grid points. In the second and third columns, we count the number of counties intersecting with different numbers of spatial grid points in 1990 and 2020. In the fourth and fifth columns, we show the percentage of children, as a share of the overall child population (ages 0–14), that reside in the counties categorized in the second and third columns. In the sixth and seventh columns, we show the corresponding cumulative percentage of children. The statistics show, for example, that in 1990, 467 counties intersect with two 0.25° × 0.25° spatial grid points, these counties account for 21.3% of the child population in 1990, and 41.2% of children reside in counties with equal or less than two intersecting UTCI spatial grid points. Panel A, depicting counties intersecting with one to five grid points accounts for approximately 82.9% and 89.4% of cumulative percentage of children in 1990 and 2020 respectively. Less than 20% of children live in counties intersecting with more than five grid points, indicating the county-level as a reasonable measure when assigning UTCI exposure for children.

A.2.3 Merging Climatic and Population Data

All project data processing, integration, and analysis code are shared at our project repository: https://github.com/ClimateInequality/PrjCEC. In this section, we summarize key aspects of how we integrate climatic and population data to enable the analysis of changes over time in heat burden facing children. Specifically, code for generating computing population-weighted exposure statistics are included in the R folder, and integrated population-climate data outputs for each analysis included in the paper are stored in the data-res folder.

In this section, we provide additional technical details on the ERA5-Heat and child population data sources and the data merging process.

ERA5-HEAT Data Input Specification To capture the entire mainland China area, we employ China's far-east (135°E), far-west (53°E), far-south (4°N), and far-north (54°N) points as spatial references in our API request to extract a rectangular area that contains gridded data covering latitude and longitude coordinates that encompass mainland China from the ERA5-HEAT data. We specify all months, dates, and hours in calendar years 1989–91 and 2019–21 in our API request. After downloading coordinate-specific hourly UTCI from all dates, we consolidate them into data files by year. For example, in the 2020 data file, corresponding to each coordinate in the gridded map (data rows), we include UTCI values for all hours between January 1, 2020 to December 31, 2020 (data columns). We perform our analysis twice, first analyzing just the 1990 and 2020 UTCI data, and again pooling the neighboring years of data from 1989–91 and 2019–21. Our results refer to our latter, more robust pooled-averages analyses.

Population Data Input Specification We obtain county-level demographic data from census tabulations. In each census file, there is one unique identification number for each county-level administrative unit. Each county includes demographic data by age group and gender for the corresponding census year. The county-level shapefile

for each census year provides geometries defining the boundaries of each county. The geometry information for each county—summarized by county-specific sets of polygon-bounding vertices in longitude and latitude units—is important for linking the population data with the gridded UTCI data.

The final population input consists of a data matrix. In this matrix, the first column identifies a distinct county-level administrative unit ID, while other columns store the proportion of the population between ages 0 to 14 in each county relative to the total population between ages 0 to 14 during one census year. While our focus is on children between ages 0 to 14, our approach has the flexibility to be extended to any demographic group as needed.

Specifying Key Files There are three key files necessary for linking population data input and UTCI input: (1) key file that links the coordinates to counties. (2) key file that links county to province and regions. (3) key file that links population input column variables to the original labels (e.g., age groups and gender), and grouping variables for aggregation purposes (i.e., age groups 0-14, 15-64, 65+).

Coordinates to counties. We use a spatial join from the "sf" package in R [13] to identify grid point coordinates from UTCI data that fall within each county boundary. When only one grid point falls within a county boundary, its attribute value is used to represent the county. When a county zone contains no grid points, the UTCI from the nearest grid point to the county centroid is assigned to the county (see Figure A.1). 127 counties in 1990 and 393 counties in 2020 do not contain grid points and require assignment. When multiple grid points fall within the county, the average of their attribute values (i.e., UTCI) is calculated to represent the county (see Figures A.2 and A.3). The final key file includes a list of coordinates, with each coordinate matched with the corresponding county-level administrative code in China. The county code provides linkages to the county-level population census, while the coordinates provide linkages to the gridded ERA5-HEAT data.

County to province/region. Each county code can be linked back to the province and

economic regions that the county belongs to. In addition to the province, we can easily aggregate the county to other higher-level units.

Population input columns to labels. This key file provides label names to the population input columns.

A.2.4 Changes in the Spatial Distribution of UTCI-Based Ambient Heat Exposure by Intensity and Duration

Figure A.4 to A.6 display the spatial distribution of the share of time exposed to three levels of UTCI-based ambient heat exposure across China in the periods 1989–91 (Panel A) and 2019-21 (Panel B): moderate or above (UTCI $\geqslant$ 26°C), strong or above (UTCI $\geqslant$ 32°C), and very strong or above (UTCI $\geqslant$ 38°C). Across all three thresholds, the maps show a clear pattern of intensifying and geographically expanding heat exposure between the two periods, with the most pronounced changes concentrated in Southeast, Central, and East China. Below, we summarize the spatial patterns and changes between these two periods.

Moderate or Above Ambient Heat Exposure (UTCI $\geqslant$ 26°C) During the 1989–91 period, areas exposed to moderate or above heat for $\geqslant$ 36% of the time (dark red) were confined to Hainan Island and a narrow strip of the far southern coast. By 2019-21, this highest category expanded substantially northward along the coast and inland, covering most of Guangdong, Guangxi, and Fujian and reaching well into South-Central China. The 27%–36% band (red-orange), which in 1989-91 formed a moderately sized, fairly contained cluster centered on Hunan and Jiangxi, expanded considerably by 2019-21 into a large, continuous region stretching across Hunan, Jiangxi, and Hubei into Fujian and Zhejiang, with a separate area of expansion also emerging around Chongqing. The 18%–27% band also pushed northward and eastward, extending across the North China Plain and into Northeast China as far as southern Liaoning, an area that showed little exposure at this level in 1989-91. In the northwest (Xinjiang), the Tarim Basin corridor of moderate-or-above exposure changed little in either

extent or intensity between the two periods.

Strong or Above Ambient Heat Exposure (UTCI $\geqslant$ 32°C) In 1989–91, the highest exposure-duration category ($\geqslant$ 12% of time; dark red) formed fragmented clusters in the Yangtze River Basin and along the southern coast, with a separate cluster in the Tarim Basin corridor of Xinjiang. By 2019-21, the mainland clusters had expanded and merged into a large, continuous region spanning Southeast and Central China, including a substantially enlarged hotspot across Hunan, Jiangxi, Hubei, and Fujian. The expansion was not limited to the traditionally hot south: the 9%–12% category, which in 1989-91 reached only into southern Anhui while most of Jiangsu remained at 6%–9%, expanded dramatically by 2019-21 to cover nearly all of Anhui and southern Jiangsu, pushing its northern edge to the Shandong, Henan, Hebei border — several hundred kilometers north of the Yangtze River. In Xinjiang, the corridor of elevated exposure also broadened and intensified, with the 9%–12% cluster growing larger and the surrounding 3%–9% areas extending further east than in 1989-91.

Very Strong or Above Ambient Heat Exposure (UTCI $\geqslant$ 38°C) Exposure to very strong or above heat for $\geqslant$ 4% of the time (dark red) was relatively rare and scattered in 1989-91, appearing mainly as small, isolated patches in East-Central China (in and around the Yangtze River Basin) and Hainan Island. By 2019-21, these patches had grown substantially larger and more numerous, coalescing into more continuous areas across the southern coast, Hainan Island, and inland into Hunan, Jiangxi, and Fujian. The Yangtze River Basin, already a relative hotspot in 1989-91, saw its high-exposure core broaden further, with the surrounding 1%–3% band extending farther inland and northward. Even the isolated Tarim Basin hotspot in Xinjiang — the only area of extreme-heat exposure outside eastern/southern China — intensified over the period, with its center shifting from the 2%–3% to the 3%–4% category and the surrounding corridor darkening as well.

Figure A.4: Spatial Distribution of Moderate or Above Heat Exposure (UTCI ⩾ 26°C) in China, 1989-91 and 2019-21

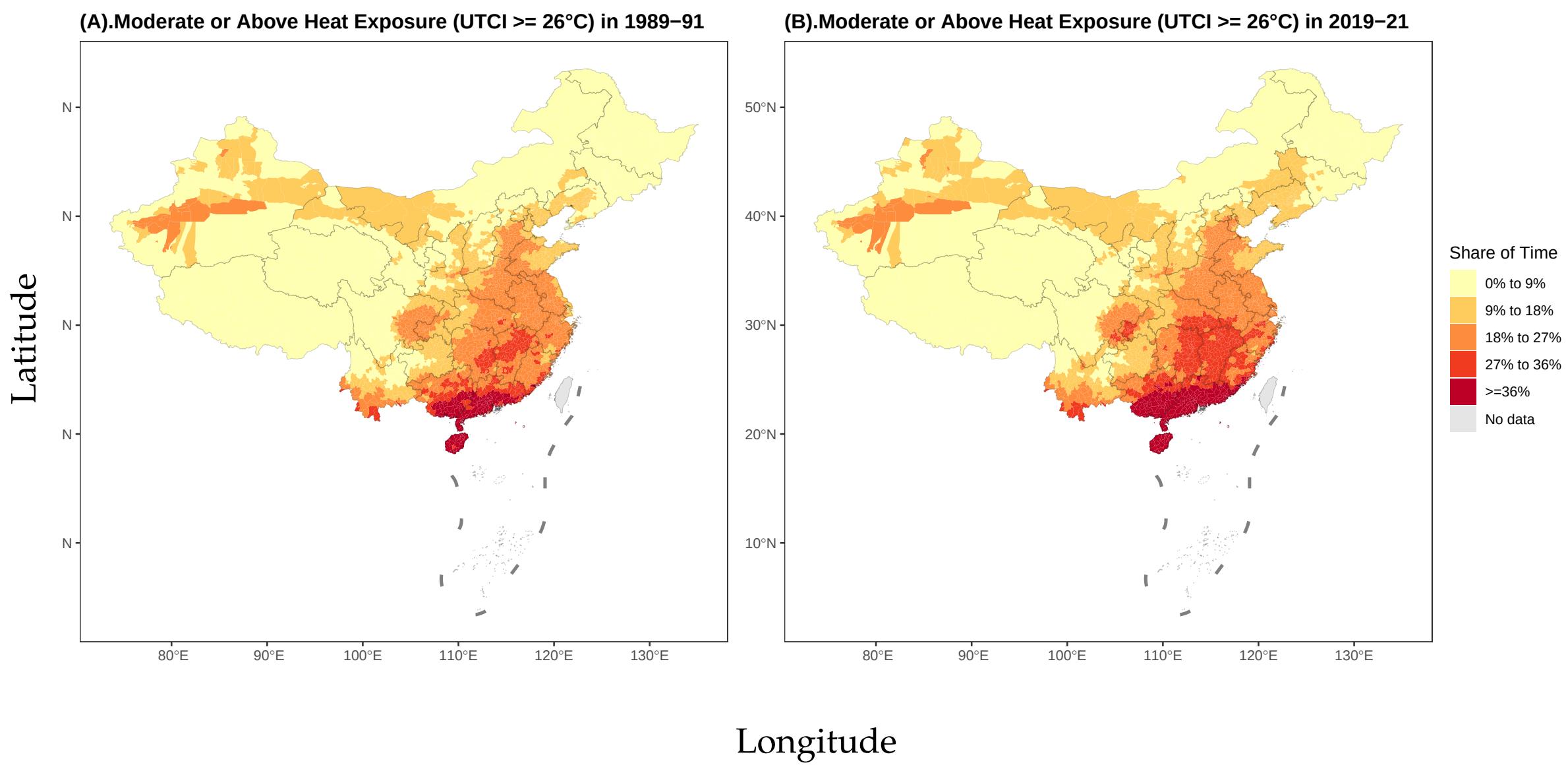

Notes: This figure displays the combined spatial distribution of the share of time exposed to Moderate or Above Heat (UTCI ⩾ 26°C) across China in the periods 1989–91 (Panel A) and 2019-21 (Panel B). Shading intensities reflect exposure shares categorized from 0% to ⩾ 36%.

Figure A.5: Spatial Distribution of Strong or Above Heat Exposure (UTCI ⩾ 32°C) in China, 1989-91 and 2019-21

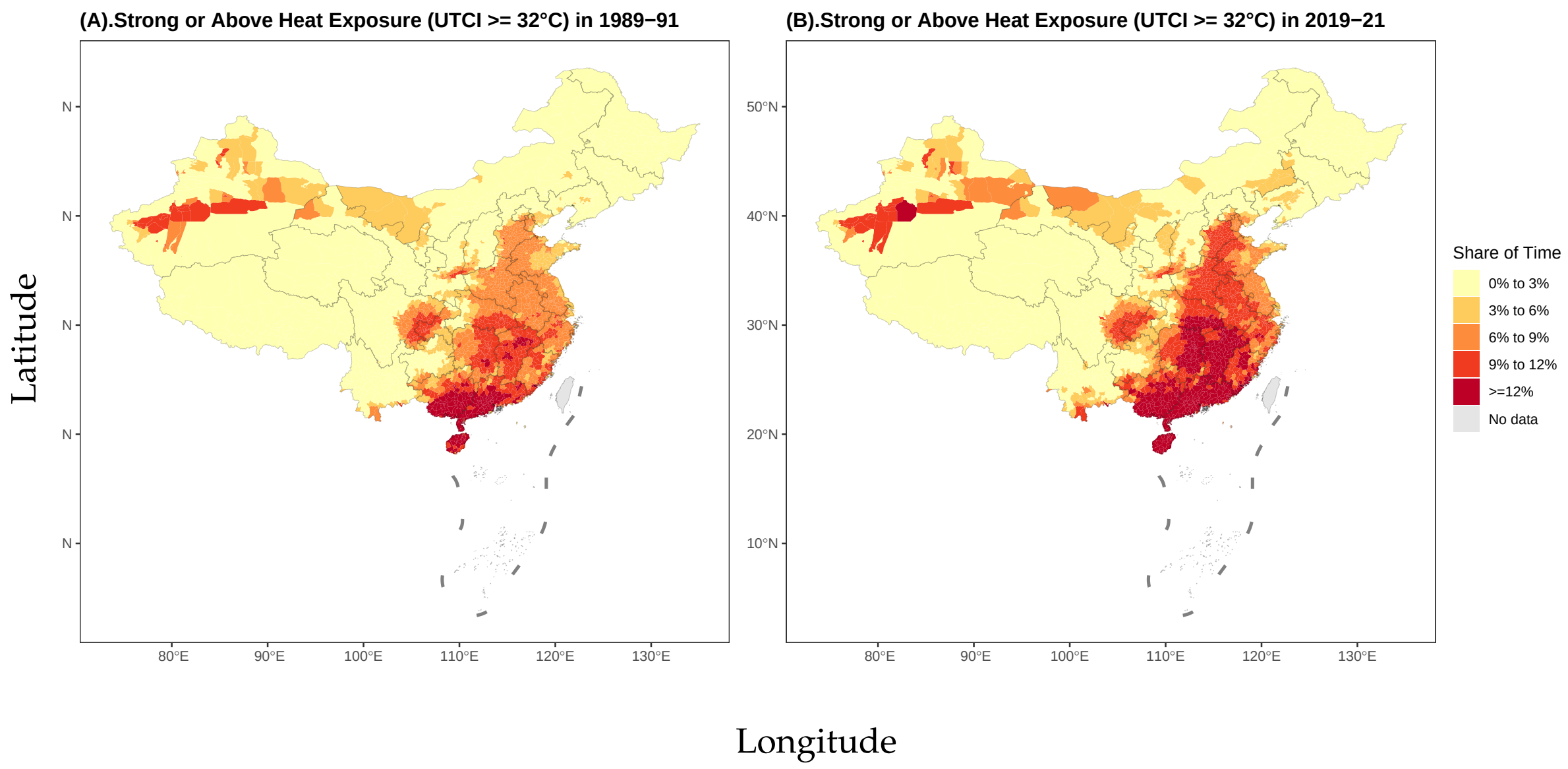

Notes: This figure displays the combined spatial distribution of the share of time exposed to Strong or Above Heat (UTCI ⩾ 32°C) across China in the periods 1989–91 (Panel A) and 2019-21 (Panel B). Shading intensities reflect exposure shares categorized from 0% to ⩾ 12%.

Figure A.6: Spatial Distribution of Very Strong or Above Heat Exposure (UTCI ⩾ 38°C) in China, 1989-91 and 2019-21

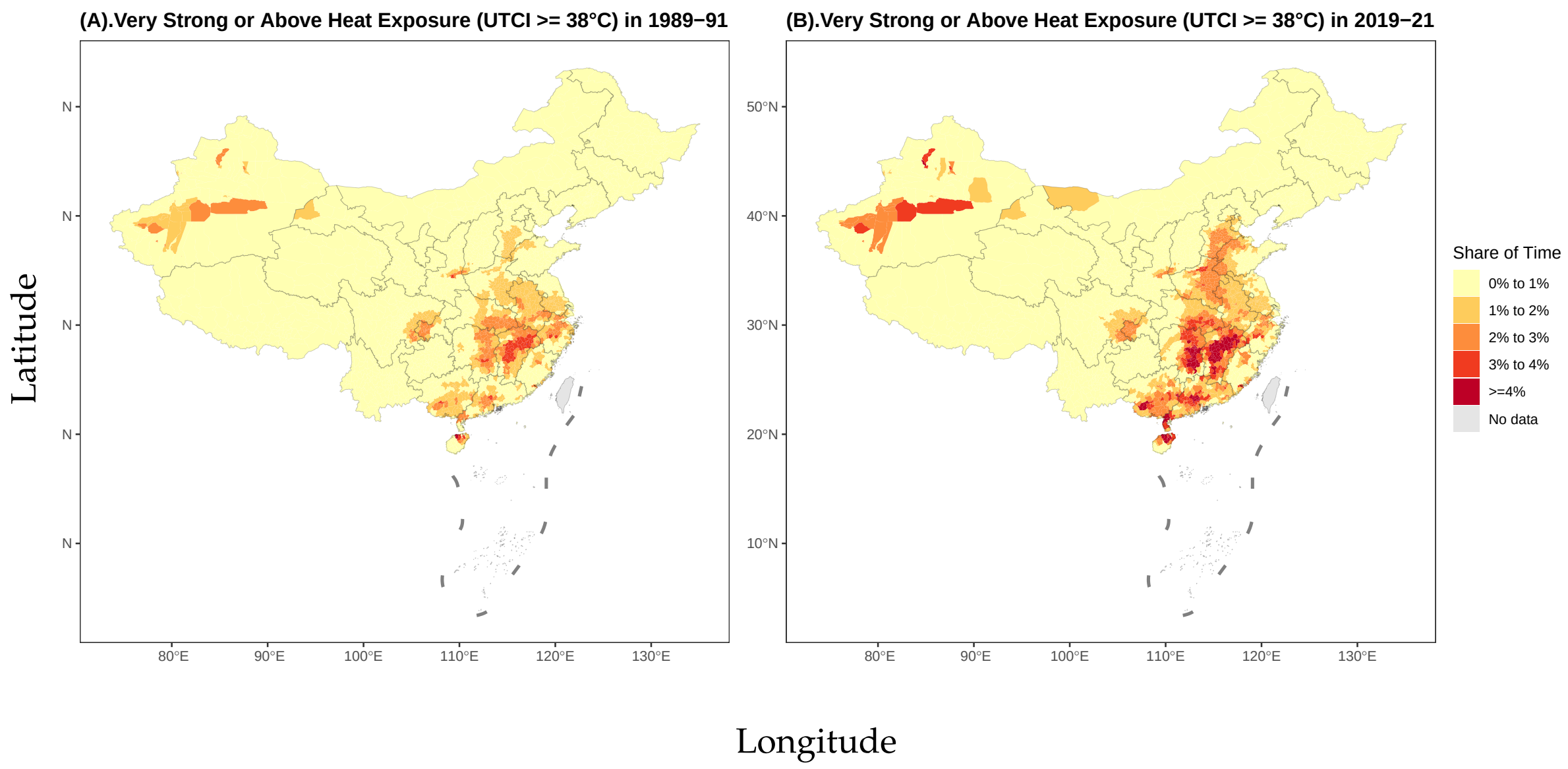

Notes: This figure displays the combined spatial distribution of the share of time exposed to Very Strong or Above Heat (UTCI ⩾ 38°C) across China in the periods 1989–91 (Panel A) and 2019-21 (Panel B). Shading intensities reflect exposure shares categorized from 0% to ⩾ 4%.

A.2.5 Changes in the Spatial Distribution of the Child Population

Spatial Distribution of the Child Population by Year Figure A.7 Panel A and B present the spatial distribution of China's child population shares across cities in 1990 and 2020, respectively. Both maps showcase a stark, enduring divide where the overwhelming majority of the child population share is concentrated eastwest of the Heihe–Tengchong Line. The western half of the country (including Tibet and parts of Qinghai/Xinjiang) remains heavily characterized by areas where the share of the child population is very small across both periods. In 1990, children were spread out evenly in massive, continuous belts of high concentration covering the North China Plain, central regions, and the Sichuan Basin. By 2020, the highest concentrations became much more fragmented and localized, suggesting that the child population has moved away from a broad regional spread and is now clustered around specific urban hubs and cities.

A major drop occurred in Northeast China. In 1990, this region had large, prominent areas with high shares of the child population. By 2020, these high-density areas vanished almost entirely, showing a significant decline in the region's share of the national child population. On the other hand, the far western tip of Xinjiang saw a noticeable increase. An area that had a relatively moderate share of children in 1990 grew into a prominent high-concentration zone by 2020. The southern and southeastern coastal areas (like Guangdong and Fujian) kept their very high concentrations of children over both decades, though these concentrations became more tightly localized around major economic centers by 2020.

Changes in the Spatial Distribution of the Child Population Figure A.8 examines changes in the spatial distribution of China's child population comparing 1990 and 2020 from two perspectives. Panel A shows percentage changes in each city's share of the national child population, while Panel B reports percentage changes in child population shares within each province. Note that, while the main analysis uses counties as the basic unit of analysis, we visualize the distribution of the child population here

at the city level because county boundaries have changed substantially between 1990 and 2020 due to processes such as mergers, dissolutions, and boundary adjustments.

Following the notation introduced in Appendix A.1, let $P_t(l \mid \text{child})$ denote the share of the national child population residing in location l at time t, such that

$$\sum_{l=1}^{L} P_t(l \mid \text{child}) = 1.$$

For the within-province analysis, let $P_t(l \mid \text{child}, p)$ denote the share of the child population residing in location l within province p, such that

$$\sum_{l \in p} P_t(l \mid \text{child}, p) = 1.$$

Specifically, Figure A.8 plots the percentage change in child population shares comparing 1990 and 2020, calculated as

$$\frac{P_{2020} - P_{1990}}{P_{1990}} \times 100,$$

where P_t denotes $P_t(l \mid \text{child})$ in Panel A and $P_t(l \mid \text{child}, p)$ in Panel B.

Figure A.8 Panel A shows that the most noticeable feature is the sharp decline in the share of the child population across the entire northeastern region of China. Cities in this region saw their share of the country's child population drop by more than half. A large blanket of cities falling into the moderate decline tiers (−50% to 0%) covers much of the central and eastern plains, showing that the child population share shrank across the country's historic heartlands. On the other hand, major economic hubs, including the Yangtze River Delta and Pearl River Delta, as well as regional capital cities such as Zhengzhou and Chengdu, saw high increases in their share of the child population.

The far western regions—especially Xinjiang and parts of Tibet—stand out by climbing into the highest growth tiers, showing that their share of the country's total child population grew significantly ($\geqslant$ 75%). The increase in this share also reflects the higher fertility rates among ethnic minority groups compared with the

Han population. However, it should be noted that the absolute number of children in western China remains substantially lower than in southeastern China.

When examining the within-province percentage change in child population shares shown in Figure A.8, Panel B, a pronounced spotted pattern emerges. Provincial capitals and primary regional cities stand out as areas of increasing child population share, even in provinces that experienced an overall decline in their national child population share, as shown in Panel A. For example, capital cities of three Northeastern provinces.

Taken togther, these patterns reflect broader trends of rapid urbanization and domestic migration in China, with populations shifting from the Northeastern and Central-Western regions to more developed coastal areas and from rural areas to major urban centers.

Figure A.7: Spatial Distribution of Child Population Share by City in China, 1990 and 2020

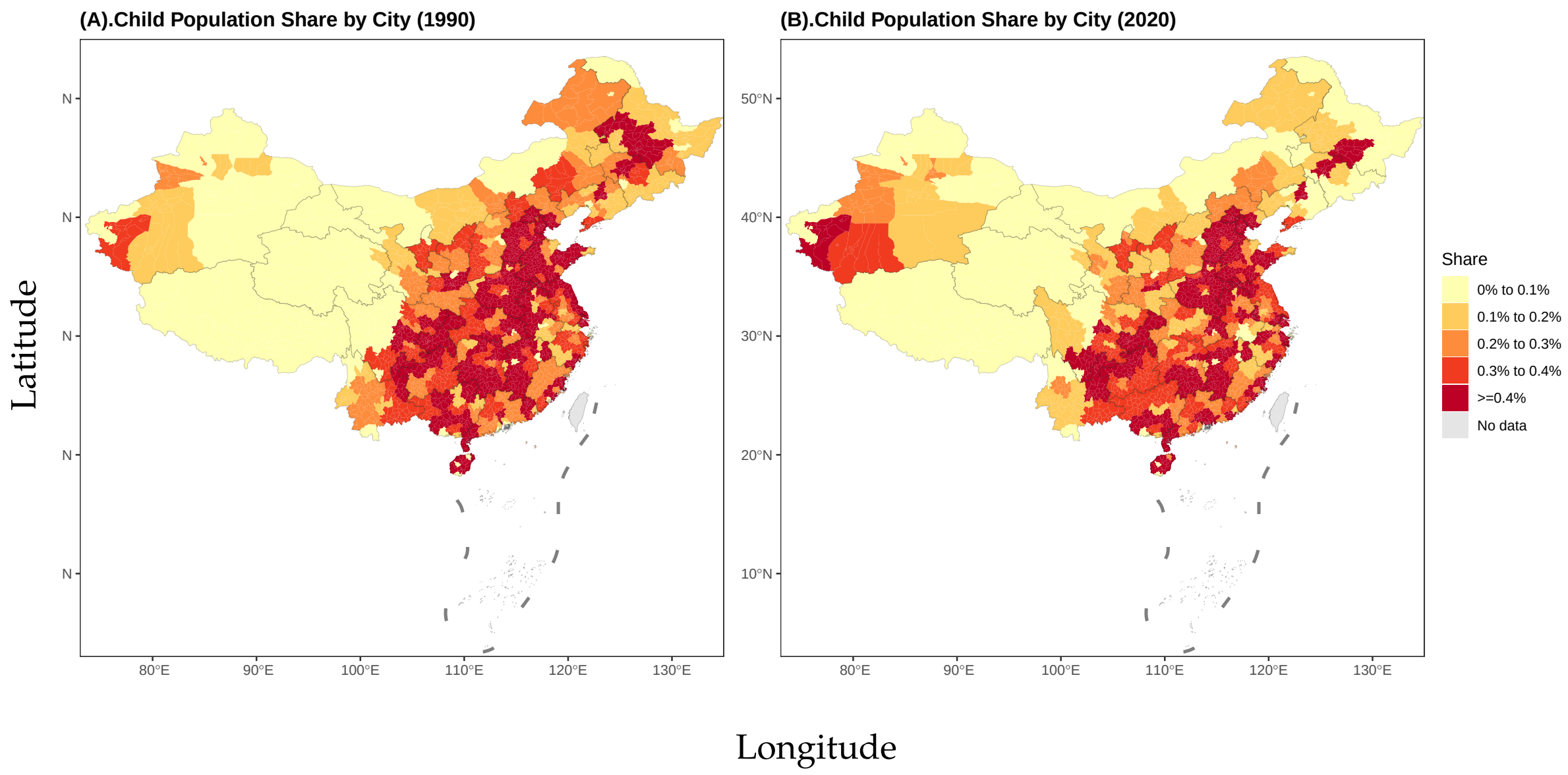

Notes: This figure displays the combined spatial distribution of the city-level child population share across China in 1990 (Panel A) and 2020 (Panel B). Shading intensities reflect shares categorized from 0% to $\geqslant 0.4\%$.

Figure A.8: Percentage Change in Child Population Shares Comparing 1990 and 2020: National Shares (Panel A) and Within-Province Shares (Panel B)

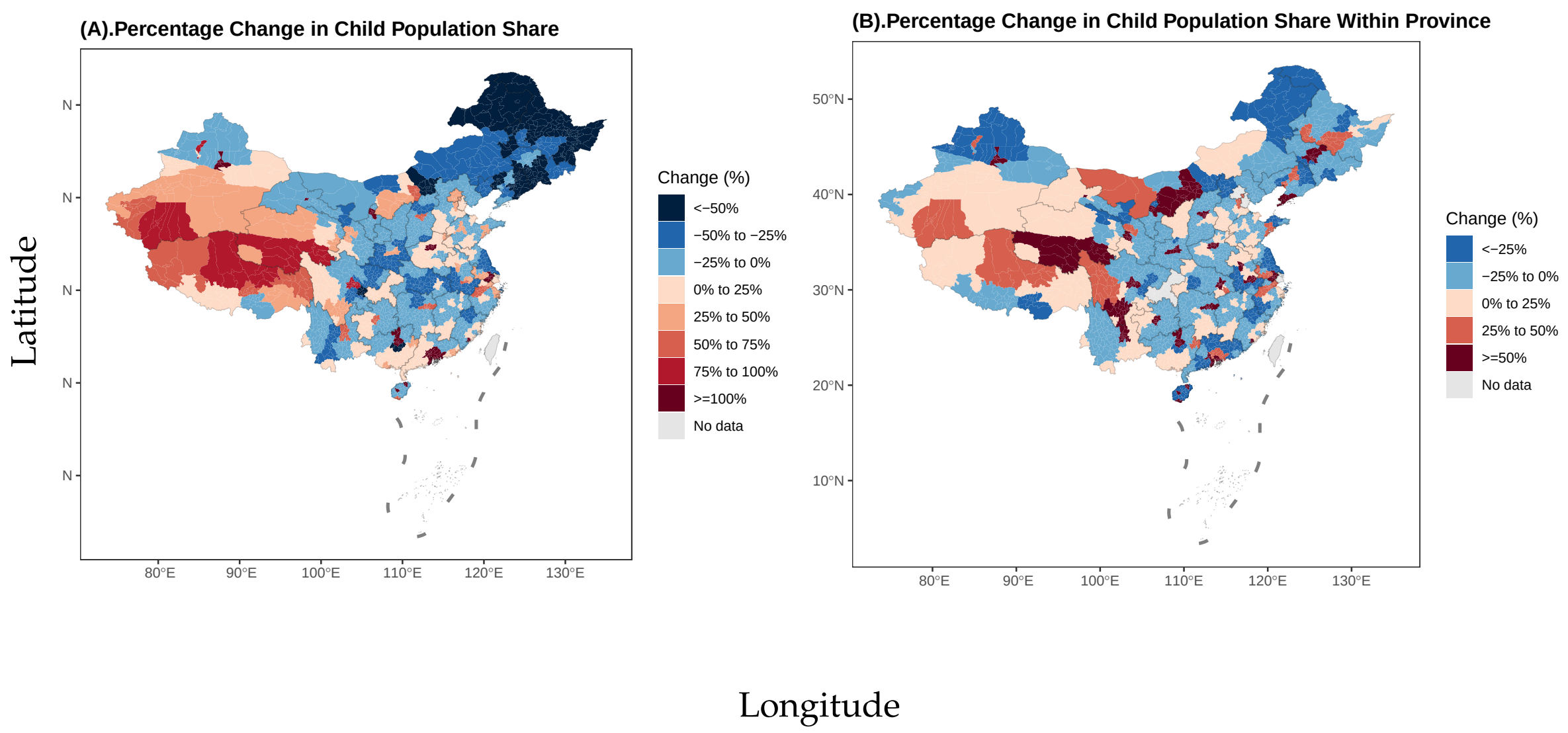

Notes: This figure displays the national percentage change in child population share (Panel A) alongside the within-province percentage change in child population share (Panel B) between the two periods, 1990 and 2020. Blue areas indicate relative decreases in child population share, while red regions signify relative increases.

B Additional Results on Heat Exposure for Children

Code and results for the additional results in this section as well as in the main text of the paper are accessible at: https://github.com/ClimateInequality/PrjCEC.

B.1 Shares of Time of Heat Exposure for the Average Child (STAC)

In Tables B.1 and B.2, we present additional details on changes in the average shares of time that Chinese children (ages 0–14) are at risk for ambient heat exposure, comparing the periods 1989–91 and 2019–21. Selected STAC results are visualized in Figure 1. We compute the annual average share of time that Chinese children are exposed to ambient heat at or above various UTCI thresholds z °C. We group thresholds by panels focusing at least borderline thermal stress (23 °C–25 °C), at least moderate heat (26 °C–31 °C), at least strong heat (32 °C–37 °C), and very strong heat (38 °C–40 °C). Again, our exposure-based measures quantify potential exposure to heat and should not be interpreted as direct measures of physiological heat stress or heat-related health risk.

Table B.1's first four columns contain our main results where we consider ambient heat exposure during all hours of 1989–91 and 2019–21. The remaining four columns in Table B.1 present results considering only daytime (between 6 am and 10 pm) hours. Table B.2 presents results where we compare average exposures in the warmer months of April, May, June, July, August, and September with exposures during the colder months of January, February, March, October, November, and December in 1989–91 and 2019–21.

Tables B.1 and B.2 show that children's share of time exposed to heat at or above various thresholds increased across all UTCI-based heat exposure categories. Specifically, Tables B.1 and B.2 show that there are between 19.4% and 33.2% increases in the average share of time that children experienced at least moderate and strong heat for all hours, daytime hours only, as well as all hours between April and September. Interestingly, Table B.2 shows that heat exposure was substantially higher during the colder months in 2019–21 compared with 1989–91, with increases ranging from 14% to

67.6% across heat exposure thresholds.

Table B.1: Changes in Average Share of Time Exposed to Heat

	All annual hours ⩾ UTCI thresholds				Day time (6 am-10 pm) hours ⩾ UTCI thresholds			
	Share of time		Changes		Share of time		Changes	
UTCI thresholds	1989-91	2019-21	Level	%	1989-91	2019-21	Level	%
**Panel A: Very strong heat**								
⩾ 40 °C	0.2%	0.4%	0.2pp	68.7%	0.4%	0.6%	0.3pp	68.7%
⩾ 39 °C	0.5%	0.8%	0.3pp	68.2%	0.7%	1.2%	0.5pp	68.2%
⩾ 38 °C	0.8%	1.4%	0.5pp	64.6%	1.3%	2.1%	0.8pp	64.6%
**Panel B: At least strong heat**								
⩾ 37 °C	1.4%	2.2%	0.8pp	61.1%	2.1%	3.3%	1.3pp	61.1%
⩾ 36 °C	2.1%	3.2%	1.2pp	55.2%	3.1%	4.9%	1.7pp	55.2%
⩾ 35 °C	3.0%	4.4%	1.5pp	49.1%	4.5%	6.7%	2.2pp	49.1%
⩾ 34 °C	4.1%	5.8%	1.7pp	42.9%	6.1%	8.7%	2.6pp	43.0%
⩾ 33 °C	5.3%	7.3%	2.0pp	37.3%	7.9%	10.9%	3.0pp	37.5%
⩾ 32 °C	6.7%	8.9%	2.2pp	32.9%	9.9%	13.2%	3.3pp	33.2%
**Panel C: At least moderate heat**								
⩾ 31 °C	8.2%	10.6%	2.4pp	29.6%	12.1%	15.7%	3.6pp	29.8%
⩾ 30 °C	9.8%	12.6%	2.7pp	27.8%	14.5%	18.4%	3.9pp	27.3%
⩾ 29 °C	11.7%	14.9%	3.2pp	27.0%	17.0%	21.2%	4.3pp	25.3%
⩾ 28 °C	13.9%	17.6%	3.7pp	26.3%	19.6%	24.2%	4.6pp	23.6%
⩾ 27 °C	16.4%	20.5%	4.1pp	25.0%	22.4%	27.3%	4.9pp	21.6%
⩾ 26 °C	19.2%	23.5%	4.4pp	22.7%	25.4%	30.4%	4.9pp	19.4%
**Panel D: At least borderline thermal stress**								
⩾ 25 °C	22.0%	26.5%	4.5pp	20.2%	28.4%	33.4%	5.0pp	17.4%
⩾ 24 °C	24.9%	29.4%	4.4pp	17.9%	31.5%	36.4%	4.9pp	15.6%
⩾ 23 °C	27.7%	32.2%	4.5pp	16.0%	34.4%	39.3%	4.9pp	14.2%

Note: Columns 1, 2, 5, and 6 show the annual average share of time at or above various UTCI-based heat exposure thresholds (UTCI at ⩾ z °C) for children in China (ages 0–14). Columns 3, 4, 7, and 8 show 1989–91 to 2019–21 changes in percentage points (level) or percentage (%) of the average shares of time at or above UTCI-based heat exposure thresholds. We consider both all hourly as well as only daytime hourly (between 6 am and 10 am) UTCI data.

Table B.2: Changes in Average Share of Time Exposed to Heat During Warmer and Colder Months

	April–September hours ⩾ UTCI thresholds				October–March hours ⩾ UTCI thresholds			
	Share of time		Changes		Share of time		Changes	
UTCI thresholds	1989-91	2019-21	Level	%	1989-91	2019-21	Level	%
**Panel A: Very strong heat**								
⩾ 40 °C	0.5%	0.8%	0.3pp	67.6%	0.00006%	0.006%	0.006pp	10,569.1%
⩾ 39 °C	1.0%	1.6%	0.6pp	66.6%	0.0004%	0.02%	0.0pp	4,489.2%
⩾ 38 °C	1.7%	2.7%	1.1pp	62.5%	0.002%	0.04%	0.0pp	1,757.6%
**Panel B: At least strong heat**								
⩾ 37 °C	2.7%	4.3%	1.6pp	58.5%	0.007%	0.09%	0.1pp	1,200.2%
⩾ 36 °C	4.1%	6.3%	2.2pp	52.4%	0.02%	0.2%	0.1pp	737.7%
⩾ 35 °C	5.9%	8.6%	2.7pp	46.3%	0.04%	0.2%	0.2pp	450.6%
⩾ 34 °C	8.0%	11.2%	3.2pp	40.4%	0.09%	0.3%	0.3pp	291.0%
⩾ 33 °C	10.4%	14.0%	3.6pp	34.9%	0.2%	0.5%	0.3pp	195.7%
⩾ 32 °C	13.0%	17.0%	4.0pp	30.7%	0.3%	0.7%	0.4pp	137.2%
**Panel C: At least moderate heat**								
⩾ 31 °C	15.9%	20.2%	4.4pp	27.7%	0.5%	0.9%	0.5pp	101.3%
⩾ 30 °C	18.9%	23.9%	4.9pp	26.0%	0.7%	1.3%	0.6pp	79.7%
⩾ 29 °C	22.4%	28.1%	5.7pp	25.3%	1.0%	1.7%	0.7pp	64.5%
⩾ 28 °C	26.3%	32.8%	6.5pp	24.8%	1.5%	2.3%	0.8pp	54.2%
⩾ 27 °C	30.7%	37.9%	7.3pp	23.6%	2.1%	3.1%	1.0pp	45.8%
⩾ 26 °C	35.4%	43.0%	7.6pp	21.3%	2.9%	4.0%	1.2pp	40.4%
**Panel D: At least borderline thermal stress**								
⩾ 25 °C	40.2%	47.7%	7.5pp	18.7%	3.8%	5.2%	1.4pp	37.1%
⩾ 24 °C	44.9%	52.1%	7.2pp	16.1%	4.8%	6.5%	1.7pp	34.9%
⩾ 23 °C	49.3%	56.2%	6.9pp	14.0%	6.0%	8.0%	2.0pp	33.4%

Note: Columns 1, 2, 5, and 6 show the annual average share of time at or above various UTCI-based heat exposure thresholds (UTCI at ⩾ z °C) for children in China (ages 0–14). Columns 3, 4, 7, and 8 show 1989–91 to 2019–21 changes in percentage points (level) or percentage (%) of the average shares of time at or above UTCI-based heat exposure thresholds. We compare UTCI in 1989–91 and 2019–21 during April, May, June, July, August and September and during January, February, March, October, November and December. We consider all 24 hours.

B.2 Share of Children Exposed by Heat Intensity and Duration Thresholds at Risk of Heat Exposure (SEIDT)

In Tables B.3 and B.4, we present additional details from the SEIDT statistics analysis of the share of children at risk of ambient heat exposure, considering the dual thresholds of intensity (UTCI thresholds $z\,^{\circ}$C) and duration (share of time-in-year thresholds y%). Selected results are visualized in Figure 2. In each scenario, the share of children is computed by aggregating the child population from locations (counties) that experienced a particular combination of intensity and duration of exposures. In Table B.3, Panels A and B present shares of children at risk of heat exposure in 1989–91 and 2019–21. In Table B.3, Panels A and B present percentage points and percentage changes between 1989–91 and 2019–21.

We find that the shares of children with prolonged ambient heat exposure associated with moderate-or-above UTCI levels were substantially higher in 2019–21 than in 1989-91. In particular, the share of children exposed to at least 12 weeks of $\geqslant 26\,^{\circ}$C ambient heat increased by more than half, from 23.7% to 36.9%. The share exposed to at least 12 weeks of $\geqslant 28\,^{\circ}$C ambient heat more than doubled, from 7.4% to 19.9%, while the share exposed to at least 12 weeks of $\geqslant 30\,^{\circ}$C ambient heat increased from 0.2% to 6.0%.

We also find a growing share of children experiencing ambient heat exposure corresponding with to least strong UTCI heat exposure levels. In particular, the share of children exposed to ambient heat at $\geqslant 34\,^{\circ}$C for at least 6 weeks increased by more than 8 times from 0.6% to 5.0%. The share exposed to ambient heat at $\geqslant 36\,^{\circ}$C for at least 4 weeks increased by more than 38 times from 0.1% to 3.8%, while the share exposed to ambient heat at $\geqslant 38\,^{\circ}$C for at least 2 weeks increased by 41 times from 0.1% to 4.1%.

Finally, not only did more children experience high intensities and long duration of heat exposure in 2019–21 compared to 1989–21, in 2019-21, children also experienced new exposure combinations at higher intensities and with longer duration beyond the 1989-91 frontier. Specifically, in 1989-91, no children experienced at least 8 weeks of

$\geqslant 34\,^{\circ}C$ UTCI, or at least 14 weeks of $\geqslant 30\,^{\circ}C$, but about 0.4% and 1.0% of children experienced these in 2019-21, respectively.

Table B.3: Share of Children at Risk of Heat Exposure, 1989–91 to 2019–21

	Share of time in year thresholds and corresponding number of weeks								
	⩾ 4%	⩾ 8%	⩾ 12%	⩾ 16%	⩾ 20%	⩾ 24%	⩾ 28%	⩾ 32%	⩾ 36%
UTCI thresholds	2 weeks	4 weeks	6 weeks	8 weeks	10 wks	12 wks	14 wks	16 wks	18 wks
**Panel A: 1989–91**									
x% (cell) of children with at least y% (column) of time in year 1990 at ⩾ z °C (row) heat threshold									
**Very strong heat**									
⩾ 38 °C	0.1%								
**At least strong heat**									
⩾ 36 °C	15.0%	0.1%							
⩾ 34 °C	56.6%	7.6%	0.6%						
⩾ 32 °C	71.0%	42.5%	8.1%	1.4%	0.2%				
**At least moderate heat**									
⩾ 30 °C	80.8%	67.7%	29.4%	10.7%	4.2%	0.2%			
⩾ 28 °C	91.6%	76.8%	66.3%	33.8%	15.6%	7.4%	4.0%	1.3%	0.1%
⩾ 26 °C	97.1%	86.1%	75.6%	66.7%	45.7%	23.7%	13.0%	9.0%	6.8%
**At least borderline thermal stress**									
⩾ 24 °C	98.8%	95.7%	84.1%	75.8%	69.2%	58.0%	33.0%	21.2%	13.1%
**Panel B: 2019–21**									
x% (cell) of children with at least y% (column) of time in year 2020 at ⩾ z °C (row) heat threshold									
**Very strong heat**									
⩾ 38 °C	4.1%								
**At least strong heat**									
⩾ 36 °C	40.8%	3.8%	0.1%						
⩾ 34 °C	68.9%	25.3%	5.0%	0.4%					
⩾ 32 °C	78.2%	62.8%	23.7%	8.8%	0.8%				
**At least moderate heat**									
⩾ 30 °C	87.0%	76.8%	58.4%	25.0%	13.5%	6.0%	1.0%		
⩾ 28 °C	94.9%	84.1%	76.1%	58.0%	28.7%	19.9%	13.1%	9.1%	6.0%
⩾ 26 °C	97.9%	92.8%	82.7%	75.9%	62.8%	36.9%	27.0%	19.6%	14.2%
**At least borderline thermal stress**									
⩾ 24 °C	98.8%	97.3%	90.8%	82.5%	77.3%	67.4%	48.6%	34.0%	25.1%

Note: Cells show the shares of Chinese children (ages 0–14) experiencing at least y% of their time in a year to ⩾ z °C UTCI. Shares of children are computed based on aggregating population shares from locations (counties) experiencing the various combinations of heat exposure duration (share of time) and intensity (UTCI thresholds). For shares of time in a year, the correspondence between the share of time and the number of weeks is based on the fact that the average of N weeks of time and $\frac{N}{4}$ months of time is approximately $(N \cdot 2)$% of total share of time in a year. To enhance contrast, values are rounded and cells with values less than 0.05% or 0.05pp are left empty. We consider all 24 hours and 12 months.

Table B.4: Change in Share of Children at Risk of Heat Exposure, 1989–91 to 2019–21

	Minimal share of time in year thresholds and corresponding number of weeks								
	⩾ 4%	⩾ 8%	⩾ 12%	⩾ 16%	⩾ 20%	⩾ 24%	⩾ 28%	⩾ 32%	⩾ 36%
UTCI thresholds	2 weeks	4 weeks	6 weeks	8 weeks	10 wks	12 wks	14 wks	16 wks	18 wks
**Panel A: 2019–21% - 1989–91%**									
Increases in percentage points (cell) of children with at least y% (column) of time at ⩾ z °C (row) heat threshold									
**Very strong heat**									
⩾ 38 °C	4.1pp								
**At least strong heat**									
⩾ 36 °C	25.8pp	3.8pp	0.1pp						
⩾ 34 °C	12.3pp	17.7pp	4.3pp	0.4pp					
⩾ 32 °C	7.2pp	20.2pp	15.7pp	7.4pp	0.6pp				
**At least moderate heat**									
⩾ 30 °C	6.3pp	9.0pp	29.0pp	14.3pp	9.3pp	5.8pp	1.0pp		
⩾ 28 °C	3.3pp	7.4pp	9.8pp	24.2pp	13.1pp	12.5pp	9.0pp	7.8pp	5.9pp
⩾ 26 °C	0.8pp	6.7pp	7.1pp	9.3pp	17.1pp	13.2pp	14.0pp	10.6pp	7.3pp
**At least borderline thermal stress**									
⩾ 24 °C	0.0pp	1.7pp	6.6pp	6.7pp	8.1pp	9.4pp	15.7pp	12.9pp	12.1pp
**Panel B:** $\frac{\textbf{2019-21\%}-\textbf{1989-91\%}}{\textbf{1989-91\%}} \cdot 100$									
Percentage increases (cell) of children with at least y% (column) of time at ⩾ z °C (row) heat threshold									
**Very strong heat**									
⩾ 38 °C	8.0k%								
**At least strong heat**									
⩾ 36 °C	172%	4.3k%							
⩾ 34 °C	21.7%	234%	685%						
⩾ 32 °C	10.2%	47.6%	195%	524%	342%				
**At least moderate heat**									
⩾ 30 °C	7.8%	13.4%	98.6%	134%	222%	2.4k%			
⩾ 28 °C	3.6%	9.6%	14.7%	71.6%	84.3%	170%	223%	620%	4.9k%
⩾ 26 °C	0.8%	7.8%	9.3%	13.9%	37.5%	55.8%	108%	118%	107%
**At least borderline thermal stress**									
⩾ 24 °C	0.0%	1.8%	7.9%	8.8%	11.7%	16.2%	47.5%	60.9%	92.5%

Note: Cells show changes comparing 1989–91 and 2019–21 periods in percentage points (Panel A) and percentage (Panel B) of the shares of Chinese children (ages 0–14) experiencing at least y% of their time in a year to ⩾ z °C UTCI. Shares of children are computed based on aggregating population shares from locations (counties) experiencing the various combinations of heat exposure duration (share of time) and intensity (UTCI thresholds). For shares of time in a year, the correspondence between the share of time and the number of weeks is based on the fact that the average of N weeks of time and $\frac{N}{4}$ months of time is approximately $(N \cdot 2)$% of total share of time in a year. To enhance contrast, values are rounded and cells with values less than 0.05% or 0.05pp are left empty. We consider all 24 hours and 12 months.

B.3 Decomposing Shifts in Population and Heat Distributions

In Tables B.5 and B.6, we provide details on the relative contributions of shifts in the child population distribution and the UTCI distribution to overall changes in the average shares of time of heat exposure for children. Selected results are visualized in Figure 3. Columns 1–3 of the tables follow from Table B.1. In columns 4–6 of the tables, we use the 1990 population distribution jointly with the 2019-21 UTCI distribution. In columns 7–9, we consider exposures if the 2020 population distribution faced the 1990 UTCI distribution. Residual unexplained changes are attributed to population and UTCI shift interactions. Our STAC-based decomposition analysis is statistical in nature: We shift one distribution while holding the other constant and do not model mechanisms of change.

In Table B.5, nationally, we show that shifts in the child population distribution between 1990 and 2020 account for 18% to 30% of the increases in the average shares of time of that children are exposed to at least moderate or at least strong heat. In contrast, within the Eastern and Northeastern regions, child population distribution shifts account for 9% to 30% of the aggregate regional increases in average child heat exposure. The national results are due to both within- and across-region shifts, whereas the regional results are attributed to only within-region shifts.

In the last column of Tables B.5 we note the decreased importance of population effects in explaining overall changes at higher UTCI thresholds. For example, nationally, the population-shift contributions to overall changes decreased from 34% for at least borderline thermal stress (UTCI $\geqslant 24\,^{\circ}\mathrm{C}$) to 18% for the upper-bound of at least strong heat (UTCI $\geqslant 36\,^{\circ}\mathrm{C}$); for the Eastern region, the corresponding numbers decreased from 36% to 20%. These mean that increases at higher heat exposure thresholds come more from increasing UTCI rather than from populations moving to locations that were already hotter in 1990.

For completeness, in Table B.6, we also present decompositions of regional changes from the Central and Western regions. Child population shifts also help to explain the relatively limited aggregate heat exposures changes in these regions.

Table B.5: Decompose Changes in Average Share of Time Exposed to Heat

	Actual 2020 vs 1990			2020 UTCI with 1990 population			1990 UTCI with 2020 population		
	Share of time		Changes	Share-time	Decompose changes		Share-time	Decompose changes	
UTCI thresholds	1990	2020	Δ	Prediction	Vs. 1990	% of Δ	Prediction	Vs. 1990	% of Δ
**Panel A: National**									
**At least strong heat**									
⩾ 36 °C	2.1%	3.2%	1.15pp	3.0%	0.89pp	77%	2.3%	0.20pp	18%
⩾ 34 °C	4.1%	5.8%	1.74pp	5.3%	1.28pp	74%	4.4%	0.38pp	22%
⩾ 32 °C	6.7%	8.9%	2.19pp	8.2%	1.54pp	70%	7.2%	0.55pp	25%
**At least moderate heat**									
⩾ 30 °C	9.8%	12.6%	2.74pp	11.7%	1.86pp	68%	10.6%	0.71pp	26%
⩾ 28 °C	13.9%	17.6%	3.67pp	16.3%	2.36pp	64%	14.9%	0.97pp	27%
⩾ 26 °C	19.2%	23.5%	4.35pp	21.9%	2.71pp	62%	20.5%	1.30pp	30%
**At least borderline thermal stress**									
⩾ 24 °C	24.9%	29.4%	4.45pp	27.6%	2.67pp	60%	26.4%	1.53pp	34%
**Panel B: Eastern region**									
**At least strong heat**									
⩾ 36 °C	2.4%	3.8%	1.34pp	3.5%	1.08pp	80%	2.7%	0.27pp	20%
⩾ 34 °C	5.0%	7.0%	2.00pp	6.5%	1.53pp	76%	5.4%	0.45pp	23%
⩾ 32 °C	8.2%	10.7%	2.50pp	10.0%	1.82pp	73%	8.8%	0.62pp	25%
**At least moderate heat**									
⩾ 30 °C	11.9%	15.2%	3.32pp	14.2%	2.28pp	69%	12.7%	0.81pp	24%
⩾ 28 °C	16.7%	21.5%	4.84pp	19.8%	3.07pp	64%	17.9%	1.22pp	25%
⩾ 26 °C	23.1%	28.6%	5.53pp	26.4%	3.36pp	61%	24.7%	1.65pp	30%
**At least borderline thermal stress**									
⩾ 24 °C	29.8%	34.9%	5.16pp	32.7%	2.94pp	57%	31.6%	1.85pp	36%
**Panel C: Northeastern region**									
**At least strong heat**									
⩾ 36 °C	0.1%	0.3%	0.14pp	0.2%	0.12pp	85%	0.1%	0.01pp	9%
⩾ 34 °C	0.5%	0.9%	0.47pp	0.9%	0.43pp	91%	0.5%	0.05pp	10%
⩾ 32 °C	1.4%	2.2%	0.80pp	2.1%	0.72pp	90%	1.5%	0.10pp	12%
**At least moderate heat**									
⩾ 30 °C	3.0%	4.1%	1.03pp	3.9%	0.91pp	89%	3.2%	0.16pp	15%
⩾ 28 °C	5.1%	6.4%	1.31pp	6.2%	1.13pp	86%	5.3%	0.21pp	16%
⩾ 26 °C	7.6%	9.2%	1.63pp	8.9%	1.35pp	83%	7.8%	0.25pp	16%
**At least borderline thermal stress**									
⩾ 24 °C	10.4%	12.3%	1.99pp	12.0%	1.61pp	81%	10.7%	0.33pp	16%

Note: Columns (cols) 1–3 include actual annual average share of time that children in China (ages 0–14) are exposed to UTCI at $\geqslant z$ °C (same as cols 1–3 in Table B.1). In cols 4–6, the 1990 population distribution face the 2019-21 UTCI distribution. In cols 7–9, 2020 population face 1989-91 UTCI. Cols 4 and 7 show annual average share of time that children are exposed to heat given decomposition scenarios. Cols 5 and 8 show differences between predictions and 1989-91 actual average shares. Cols 6 and 9 show the share of column 3 actual changes that the predictions from cols 5 and 8 account for. We consider all 24 hours and 12 months.

Table B.6: Decompose Changes in Average Share of Time Exposed to Heat

	Actual 2019-21 vs 1989-91			2019-21 UTCI with 1990 population			1989-91 UTCI with 2020 population		
	Share of time		Changes	Share-time	Decompose changes		Share-time	Decompose changes	
UTCI thresholds	1989-91	2019-21	Δ	Prediction	Vs. 1989-91	% of Δ	Prediction	Vs. 1989-91	% of Δ
**Panel A: Central region**									
**At least strong heat**									
⩾ 36 °C	2.9%	4.4%	1.47pp	4.3%	1.37pp	93%	3.0%	0.04pp	3%
⩾ 34 °C	5.2%	7.3%	2.06pp	7.1%	1.94pp	94%	5.3%	0.07pp	3%
⩾ 32 °C	8.1%	10.4%	2.37pp	10.3%	2.22pp	94%	8.2%	0.09pp	4%
**At least moderate heat**									
⩾ 30 °C	11.6%	14.2%	2.56pp	14.1%	2.41pp	94%	11.7%	0.11pp	4%
⩾ 28 °C	16.1%	18.9%	2.82pp	18.8%	2.67pp	95%	16.2%	0.10pp	4%
⩾ 26 °C	21.5%	24.6%	3.04pp	24.4%	2.91pp	96%	21.6%	0.10pp	3%
**At least borderline thermal stress**									
⩾ 24 °C	27.3%	30.5%	3.24pp	30.4%	3.10pp	96%	27.4%	0.11pp	3%
**Panel B: Western region**									
**At least strong heat**									
⩾ 36 °C	1.4%	1.9%	0.47pp	1.8%	0.39pp	82%	1.4%	0.04pp	9%
⩾ 34 °C	2.9%	3.6%	0.72pp	3.4%	0.58pp	81%	3.0%	0.10pp	13%
⩾ 32 °C	4.9%	5.9%	0.94pp	5.7%	0.77pp	81%	5.1%	0.14pp	15%
**At least moderate heat**									
⩾ 30 °C	7.6%	8.9%	1.27pp	8.7%	1.07pp	84%	7.7%	0.17pp	13%
⩾ 28 °C	11.0%	12.9%	1.83pp	12.6%	1.57pp	86%	11.3%	0.21pp	11%
⩾ 26 °C	15.6%	18.1%	2.45pp	17.8%	2.16pp	88%	15.9%	0.25pp	10%
**At least borderline thermal stress**									
⩾ 24 °C	21.0%	23.4%	2.48pp	23.2%	2.24pp	90%	21.2%	0.24pp	10%

Note: Columns (cols) 1–3 include actual annual average share of time that children in the Central and Western regions of China (ages 0–14) are exposed to UTCI at $\geqslant z\ ^\circ$C (same as cols 1–3 in Table B.1). In cols 4–6, the 1990 population distribution face the 2019-21 UTCI distribution. In cols 7–9, 2020 population face 1989-91 UTCI. Cols 4 and 7 show annual average share of time that children are exposed to heat given decomposition scenarios. Cols 5 and 8 show differences between predictions and 1989-91 actual average shares. Cols 6 and 9 show the share of column 3 actual changes that the predictions from cols 5 and 8 account for. We consider all 24 hours and 12 months.

B.4 Additional Regional Analysis

In Tables B.7 and B.8, we present details on region- and province-specific STAC child heat exposure analysis. Selected regional results are visualized in Figure 4. Subnational analyses show which areas have experienced greater changes in heat exposures and also shed light on whether aggregate changes are due to population shifts across provinces within regions or across regions.[B.1] Table B.7 presents results for at least strong and very strong heat and Table B.8 focuses on at least moderate heat.

Between 1989-91 and 2019-21, while Central (C) and Western (W) heat exposures stagnated, children in the heated Eastern (E) and colder Northeastern (NE) regions experienced respective increases up to 78% and 109% in average heat exposure time. In 2019-21, the average E, NE, C, and W region child experienced 10.7%, 2.2%, 10.4%, and 5.9% of her time under at least strong (UTCI $\geqslant 32\,^{\circ}$C) heat.

In 2019-21, we find that children in Hainan (E), Guangdong (E), Guangxi (W), Jiangxi (C), and Hunan (C) had the highest heat exposures with 19.6%, 16.2%, 14.6%, 13.6%, and 12.1% of their average shares of time exposed to at least strong heat (UTCI $\geqslant 32\,^{\circ}$C), which represented respective increases of 22%, 24%, 27%, 28%, and 30% compared to 1989-91.

Lastly, we note the importance of considering both changes and levels. While Hebei (E), Zhejiang (E), Heilongjiang (NE), and Jilin (NE) provinces experienced small percentage point increases in the average share of heat exposure time, the percentage increases are between 2 to 95 times larger due to lower starting levels. Additionally, Jilin (NE) and Liaoning (NE) arrived at similar levels of average child heat exposure in 2019-21 with increases of 89% and 26% in average share of heat exposure time, respectively. Locations with similar levels or changes of exposure might require different societal and physical adjustments depending on prior levels and the magnitudes of recent changes.

[B.1] Even when there are no changes in UTCI and within-region population distributions, average national child exposure could increase due to shifts in child population to hotter regions.

Table B.7: Regional Average Share of Time at Risk of Exposure to At-Least Strong or Very Strong Heat for Children (Ages 0-14), 1989-91 to 2019-21

	At least strong heat stress								Very strong heat stress			
	⩾ UTCI 32° C				⩾ UTCI 35° C				⩾ UTCI 38° C			
	Share of time		Changes		Share of time		Changes		Share of time		Changes	
Location	1989-91	2019-21	Level	%	1989-91	2019-21	Level	%	1989-91	2019-21	Level	%
**Panel A: Regions**												
Eastern	8.2%	10.7%	2.5pp	30%	3.6%	5.3%	1.7pp	47%	0.9%	1.5%	0.7pp	78%
Northeastern	1.4%	2.2%	0.8pp	56%	0.2%	0.5%	0.3pp	114%	0.0%	0.0%	0.0pp	109%
Central	8.1%	10.4%	2.4pp	29%	4.0%	5.8%	1.8pp	45%	1.4%	2.1%	0.7pp	54%
Western	4.9%	5.9%	0.9pp	19%	2.0%	2.7%	0.6pp	30%	0.5%	0.7%	0.2pp	38%
**Panel B: Eastern region**												
Beijing	3.0%	6.4%	3.3pp	108%	0.7%	2.6%	1.9pp	254%	0.1%	0.6%	0.5pp	619%
Fujian	8.9%	12.0%	3.2pp	36%	3.5%	5.4%	1.9pp	54%	0.6%	1.1%	0.5pp	73%
Guangdong	13.1%	16.2%	3.1pp	24%	5.9%	8.2%	2.3pp	40%	1.3%	2.3%	1.0pp	75%
Hainan	16.1%	19.6%	3.6pp	22%	6.3%	9.6%	3.3pp	52%	1.0%	2.9%	1.8pp	179%
Hebei	6.6%	8.3%	1.7pp	26%	2.9%	4.4%	1.5pp	51%	0.7%	1.5%	0.8pp	111%
Jiangsu	6.9%	8.3%	1.4pp	20%	3.2%	3.8%	0.6pp	18%	1.0%	1.2%	0.1pp	13%
Shandong	6.5%	7.9%	1.4pp	21%	2.5%	3.9%	1.3pp	53%	0.4%	1.2%	0.8pp	177%
Shanghai	5.1%	6.0%	1.0pp	19%	1.9%	2.4%	0.5pp	27%	0.5%	0.6%	0.0pp	9%
Tianjin	5.8%	7.7%	1.9pp	32%	2.2%	3.8%	1.6pp	72%	0.3%	1.0%	0.7pp	225%
Zhejiang	8.0%	9.2%	1.2pp	15%	4.2%	4.5%	0.3pp	7%	1.5%	1.4%	-0.1pp	-7%
**Panel C: Northeastern region**												
Heilongjiang	1.0%	1.8%	0.8pp	76%	0.2%	0.4%	0.2pp	106%	0.0%	0.0%	0.0pp	-24%
Jilin	1.1%	2.1%	1.0pp	89%	0.2%	0.4%	0.3pp	180%	0.0%	0.0%	0.0pp	13%
Liaoning	2.1%	2.6%	0.5pp	26%	0.4%	0.7%	0.3pp	83%	0.0%	0.1%	0.1pp	248%
**Panel D: Central region**												
Anhui	8.2%	10.1%	1.9pp	24%	4.2%	5.3%	1.1pp	26%	1.5%	1.8%	0.2pp	15%
Henan	7.6%	10.0%	2.4pp	32%	3.5%	5.5%	2.0pp	56%	1.0%	2.0%	1.0pp	100%
Hubei	8.3%	10.6%	2.3pp	28%	4.2%	5.9%	1.7pp	41%	1.5%	2.1%	0.5pp	35%
Hunan	9.3%	12.1%	2.8pp	30%	4.4%	6.8%	2.4pp	55%	1.5%	2.6%	1.1pp	71%
Jiangxi	10.8%	13.6%	2.9pp	27%	5.9%	8.1%	2.2pp	37%	2.4%	3.4%	0.9pp	39%
Shanxi	2.5%	3.2%	0.8pp	32%	0.7%	1.0%	0.3pp	39%	0.2%	0.2%	0.1pp	33%
**Panel E: Western region**												
Gansu	0.8%	1.1%	0.3pp	36%	0.1%	0.2%	0.1pp	58%	0.0%	0.0%	0.0pp	67%
Guangxi	12.0%	14.6%	2.6pp	21%	5.1%	7.5%	2.4pp	46%	1.2%	2.0%	0.8pp	68%
Guizhou	2.2%	2.7%	0.5pp	21%	0.4%	0.5%	0.1pp	21%	0.0%	0.0%	0.0pp	17%
Neimenggu	1.3%	1.9%	0.6pp	50%	0.3%	0.5%	0.2pp	79%	0.0%	0.1%	0.1pp	148%

Continued on next page

Table B.7: Regional Average Share of Time at Risk of Exposure to At-Least Strong or Very Strong Heat for Children (Ages 0-14), 1989-91 to 2019-21

	At least strong heat stress								Very strong heat stress			
	⩾ UTCI 32° C				⩾ UTCI 35° C				⩾ UTCI 38° C			
	Share of time		Changes		Share of time		Changes		Share of time		Changes	
Location	1989-91	2019-21	Level	%	1989-91	2019-21	Level	%	1989-91	2019-21	Level	%
Ningxia	2.0%	3.1%	1.0pp	50%	0.6%	1.0%	0.4pp	60%	0.1%	0.2%	0.0pp	45%
Qinghai	0.0%	0.0%	0.0pp	0.0%	0.0%	0.0%	0.0pp	0.0%	0.0%	0.0%	0.0pp	0.0%
Shaanxi	4.0%	4.7%	0.7pp	16%	1.6%	1.8%	0.2pp	13%	0.5%	0.4%	-0.1pp	-14%
Sichuan	7.1%	7.4%	0.3pp	4.2%	3.3%	3.6%	0.3pp	9.1%	0.9%	1.1%	0.2pp	22.2%
Xinjiang	3.7%	5.7%	2.0pp	54%	1.6%	2.8%	1.2pp	73%	0.5%	1.0%	0.5pp	111%
Xizang	0.0%	0.0%	0.0pp	0.0%	0.0%	0.0%	0.0pp	0.0%	0.0%	0.0%	0.0pp	0.0%
Yunnan	0.9%	1.4%	0.6pp	64%	0.0%	0.1%	0.1pp	180%	0.0%	0.0%	0.0pp	1.7k%

Note: We present similar statistics as in Table B.1, but now compute exposures separately for the four economic regions and provincial-level administrative units in China. Columns (cols) 1–3 and 4–6 focus on at least strong UTCI heat exposure at ⩾ 32 °C and ⩾ 35 °C, respectively. Cols 7–9 focus on very strong UTCI heat exposure at ⩾ 38 °C. Cols 1 and 2, 5 and 6, and 9 and 10 show the annual average share of time at or above various UTCI-based heat exposure thresholds (UTCI at ⩾ z °C) for children in China (ages 0–14). Cols 3 and 4, 7 and 8, and 11 and 12 show 1989–91 to 2019–21 changes in percentage points (level) or percentage (%) of the average shares of time at or above UTCI-based heat exposure thresholds. Cells are empty for percentage changes when the denominator is equal to zero. We consider all 24 hours and 12 months.

Table B.8: Regional Average Share of Time at Risk of Exposure to At-Least Moderate Heat for Children (ages 0-14), 1989-91 to 2019-21

	At least borderline thermal stress				At least moderate heat stress							
	⩾ UTCI 23° C				⩾ UTCI 26° C				⩾ UTCI 29° C			
	Share of time		Changes		Share of time		Changes		Share of time		Changes	
Location	1989-91	2019-21	Level	%	1989-91	2019-21	Level	%	1989-91	2019-21	Level	%
**Panel A: Regions**												
Eastern	32.9%	37.9%	5.0pp	15%	23.1%	28.6%	5.5pp	24%	14.1%	18.1%	4.0pp	29%
Northeastern	11.9%	14.1%	2.2pp	18%	7.6%	9.2%	1.6pp	22%	4.0%	5.2%	1.2pp	29%
Central	30.2%	33.5%	3.3pp	11%	21.5%	24.6%	3.0pp	14%	13.8%	16.4%	2.7pp	19%
Western	23.7%	26.2%	2.4pp	10%	15.6%	18.1%	2.5pp	16%	9.2%	10.7%	1.5pp	17%
**Panel B: Eastern region**												
Beijing	18.6%	23.4%	4.8pp	26%	12.2%	16.4%	4.3pp	35%	7.1%	11.0%	4.0pp	56%
Fujian	39.8%	46.4%	6.6pp	17%	26.0%	33.1%	7.1pp	27%	15.3%	19.8%	4.5pp	29%
Guangdong	51.9%	57.2%	5.4pp	10%	37.8%	46.3%	8.5pp	22%	22.0%	28.5%	6.5pp	30%
Hainan	62.5%	66.1%	3.6pp	6%	46.2%	52.8%	6.6pp	14%	27.4%	33.0%	5.7pp	21%
Hebei	23.9%	25.6%	1.7pp	7%	16.8%	18.5%	1.7pp	10%	10.9%	12.9%	1.9pp	18%
Jiangsu	27.8%	30.5%	2.7pp	10%	19.5%	21.7%	2.2pp	11%	12.1%	14.2%	2.1pp	17%
Shandong	24.9%	26.1%	1.1pp	5%	17.4%	18.6%	1.3pp	7%	11.3%	12.6%	1.4pp	12%

Continued on next page

Table B.8: Regional Average Share of Time at Risk of Exposure to At-Least Moderate Heat for Children (ages 0-14), 1989-91 to 2019-21

	At least borderline thermal stress				At least moderate heat stress							
	⩾ UTCI 23° C				⩾ UTCI 26° C				⩾ UTCI 29° C			
	Share of time		Changes		Share of time		Changes		Share of time		Changes	
Location	1989-91	2019-21	Level	%	1989-91	2019-21	Level	%	1989-91	2019-21	Level	%
Shanghai	26.2%	30.2%	4.0pp	15%	16.5%	20.8%	4.2pp	26%	9.9%	11.8%	1.8pp	18%
Tianjin	23.0%	25.2%	2.2pp	10%	16.0%	18.2%	2.2pp	14%	10.1%	12.5%	2.4pp	24%
Zhejiang	33.4%	36.5%	3.1pp	9%	22.6%	26.2%	3.7pp	16%	13.4%	15.6%	2.2pp	16%
**Panel C: Northeastern region**												
Heilongjiang	10.1%	11.8%	1.8pp	17%	6.3%	7.7%	1.4pp	22%	3.1%	4.3%	1.2pp	38%
Jilin	11.0%	13.0%	2.0pp	19%	6.9%	8.6%	1.7pp	24%	3.6%	5.0%	1.4pp	39%
Liaoning	14.4%	16.2%	1.8pp	13%	9.3%	10.6%	1.3pp	14%	5.3%	5.9%	0.7pp	13%
**Panel D: Central region**												
Anhui	30.2%	33.0%	2.8pp	9%	22.0%	23.8%	1.8pp	8%	13.9%	16.1%	2.1pp	15%
Henan	27.5%	30.1%	2.6pp	10%	19.6%	21.7%	2.1pp	11%	12.7%	15.0%	2.3pp	18%
Hubei	31.0%	34.6%	3.6pp	12%	22.4%	25.6%	3.2pp	14%	14.4%	17.1%	2.7pp	19%
Hunan	34.9%	38.8%	3.9pp	11%	24.3%	28.8%	4.5pp	18%	15.6%	19.0%	3.3pp	21%
Jiangxi	38.0%	42.9%	4.9pp	13%	27.3%	32.7%	5.4pp	20%	17.6%	21.7%	4.0pp	23%
Shanxi	15.8%	17.1%	1.3pp	8%	10.4%	11.7%	1.3pp	13%	5.8%	7.1%	1.2pp	21%
**Panel E: Western region**												
Gansu	10.7%	11.3%	0.6pp	6%	6.3%	6.9%	0.6pp	10%	2.8%	3.4%	0.6pp	21%
Guangxi	46.9%	51.1%	4.2pp	9%	32.6%	38.9%	6.3pp	19%	19.8%	23.5%	3.6pp	18%
Guizhou	18.3%	20.5%	2.2pp	12%	11.2%	12.7%	1.4pp	13%	6.1%	7.0%	0.9pp	15%
Neimenggu	10.1%	12.0%	1.9pp	19%	6.4%	8.2%	1.8pp	28%	3.3%	4.6%	1.3pp	39%
Ningxia	12.4%	14.3%	1.9pp	15%	8.3%	10.1%	1.8pp	22%	4.6%	6.2%	1.5pp	33%
Qinghai	4.6%	4.2%	-0.4pp	-9%	1.4%	1.3%	-0.1pp	-8%	0.1%	0.2%	0.0pp	17%
Shaanxi	18.8%	19.9%	1.1pp	6%	12.8%	13.6%	0.8pp	6%	8.0%	8.8%	0.8pp	10%
Sichuan	27.8%	30.0%	2.2pp	7.9%	18.5%	20.2%	1.7pp	9.2%	11.7%	12.5%	0.8pp	6.8%
Xinjiang	15.4%	18.3%	2.9pp	19%	10.6%	13.5%	2.9pp	27%	6.7%	9.2%	2.5pp	37%
Xizang	1.3%	1.4%	0.1pp	5%	0.1%	0.1%	0.0pp	4%	0.0%	0.0%	0.0pp	158%
Yunnan	19.1%	21.9%	2.8pp	15%	11.0%	13.3%	2.3pp	21%	4.6%	6.0%	1.4pp	31%

Note: We present similar statistics as in Table B.1, but now compute exposures separately for the four economic regions and provincial-level administrative units in China. Columns (cols) 4–6 and 7–9 focus on at least moderate UTCI heat exposure at ⩾ 26 °C and ⩾ 29 °C, respectively. Cols 1–3 provide UTCI heat exposure at ⩾ 23 °C—UTCI 23 °C is a level that is just below the UTCI 25 °C threshold for moderate heat. Cols 1 and 2, 5 and 6, and 9 and 10 show the annual average share of time at or above various UTCI-based heat exposure thresholds (UTCI at ⩾ z °C) for children in China (ages 0–14). Cols 3 and 4, 7 and 8, and 11 and 12 show 1989–91 to 2019–21 changes in percentage points (level) or percentage (%) of the average shares of time at or above UTCI-based heat exposure thresholds. Cells are empty for percentage changes when the denominator is equal to zero. We consider all 24 hours and 12 months.

B.5 Main and Additional Results Replication

Code and results for the additional results in this section as well as in the main text of the paper are accessible at: https://github.com/ClimateInequality/PrjCEC.

Code for generating the statistics shown in tables and figures are stored in the R-script folder, and code and output for visualizing and tabularization are stored in the res folder.

1. Section B.1 and main text Figure 1 results and code:
   - Generate statistics: R-script/run_1a_mean_child_all24, R-script/run_1b_mean_child_6t22, and R-script/run_1c_mean_child_seasons
   - Tabulate and visualize: R-script/tabfig_1_mean_child
   - Tables and figures: res/res_mean_child
2. Section B.2 and main text Figure 2 results and code:
   - Generate statistics: R-script/run_2a_atrisk_child
   - Tabulate and visualize: R-script/tabfig_2_at_risk
   - Tables and figures: res/res_atrisk
3. Section B.3 and main text Figure 3 results and code:
   - Generate statistics: R-script/run_3a_decompose and R-script/run_3b_decompose_regional
   - Tabulate and visualize: R-script/tabfig_3_decompose
   - Tables and figures: res/res_decompose
4. Section B.4 and main text Figure 4 results and code:
   - Generate statistics: R-script/run_4a_mean_child_all24_by_region and R-script/run_4b_mean_child_all24_by_province
   - Tabulate and visualize: R-script/tabfig_4_region_prov
   - Tables and figures: res/res_region_prov

C Robustness checks

C.1 Hours exposed to heat and days in heat spell

We analyze the relationship between the share of hours in which the UTCI exceeds a heat threshold and the share of days in a heat spell—defined as a run of consecutive days during which daily maximum UTCI exceeds the same heat threshold. Using hourly UTCI data for China's counties across two three-year windows (1989–1991 and 2019–2021), we find that the two measures are highly correlated.

We consider three heat thresholds $c^* \in \{26, 32, 38\}$ °C UTCI. Additionally, we consider heat spells during which daily maximum UTCI $\geqslant c^*$ for at least $d \in \{3, 5\}$ consecutive days. As in our main analysis, grid-point statistics are averaged to the county level and then merged with census data (1990 and 2020 rounds) to obtain the child population weights for each county.

Table C.1: Child-Population-Weighted County-level Correlations between Share of Days in Heat Spell Share of Hours Exceeding Heat Threshold.

	Heat spell consecutive days thresholds	
Temperature Threshold	3 consecutive days	5 consecutive days
China county-level correlations **1989 to 1991**		
Daily max or hourly UTCI ⩾ 26°C	0.949	0.942
Daily max or hourly UTCI ⩾ 32°C	0.981	0.977
Daily max or hourly UTCI ⩾ 38°C	0.978	0.954
China county-level correlations **2019 to 2021**		
Daily max or hourly UTCI ⩾ 26°C	0.946	0.940
Daily max or hourly UTCI ⩾ 32°C	0.984	0.982
Daily max or hourly UTCI ⩾ 38°C	0.982	0.959

Table C.1 reports population-weighted correlations between the heat-hours share and the heat-spell-day share for each combination of c^*, d, and time period. Correlations are uniformly high across all cells. For $c^* = 26$ °C UTCI, correlations range between 0.940 and 0.949 for both sets of years. The intermediate threshold $c^* = 32$ °C UTCI produces the highest correlations overall, peaking at 0.984 (2019–2021, $d \geqslant 3$) and remaining above 0.977 throughout. At the extreme threshold $c^* = 38$ °C UTCI, correlations range from 0.954 to 0.982. We also show in Figure C.1 child-population-weighted scatter plots for results at the higher UTCI thresholds.

The results show that our measure of heat, based on the share of hours during which the UTCI exceeds a given heat thresholds, also captures heat spells well in our empirical setting. Our measure provides a more complete accounting of children's total exposure to heat, considering all hours of ambient heat exposure. Our measure also has the added benefit of being simpler to compute and interpret, and does not vary with an additional and potentially arbitrary consecutive-days threshold parameter d for heat spells.

Figure C.1: Share of Days in Heat Spell and Share of Hours in Heat Exposure.

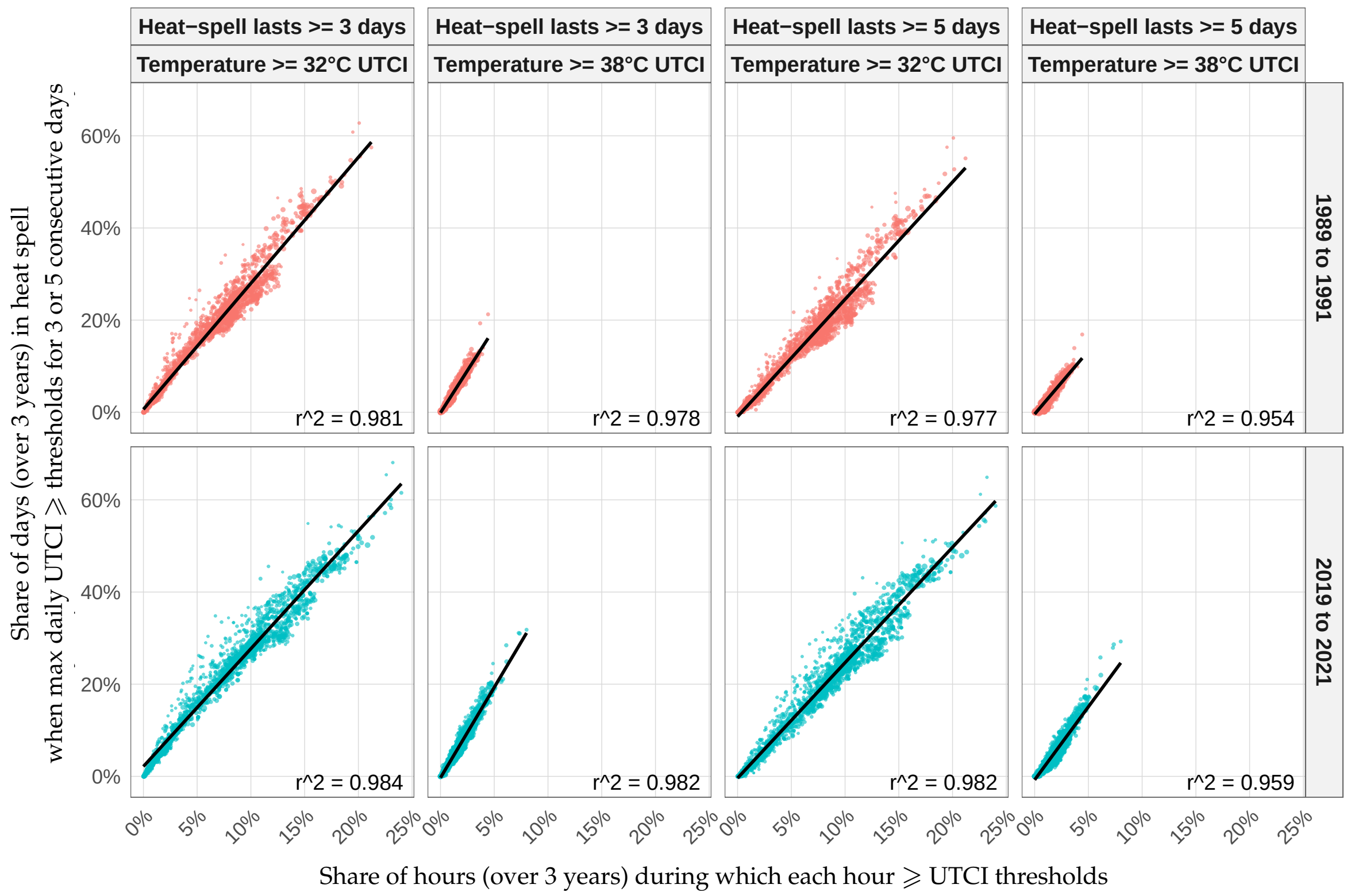

Notes: This figure presents scatter plots examining the relationship between the share of hours exceeding UTCI thresholds and the share of days classified as heat spells. Each point represents a Chinese county, with point size scaled by the child population weight. The row subplots represent the two time periods (1989–1991 and 2019–2021) and columns represent four combinations of heat thresholds (32 °C and 38 °C UTCI, as either the hourly or daily max thresholds) and heat-spell duration thresholds ($\geqslant$ 3 consecutive days and $\geqslant$ 5 consecutive days). Within each subplot, a population-weighted linear regression line (black) is shown and the r^2 is displayed in the lower-right corner of each panel.

C.2 Matching population data to one year of heat exposure data

We replicate our main analyses that use the three-year UTCI windows, between 1989-91 and 2019-21, using data from the single years of 1990 and 2020. Because our population data is from 1990 and 2020, using a single year's UTCI data might provide a better match with the population as captured in the 1990 and 2020 censuses. However, because UTCI fluctuate across years, using a single year of UTCI data may not be ideal to capture the overall patterns of changes across time. In our main text, we use the triple-year UTCI analysis results in order to better capture average patterns of heat exposure changes between the two periods. In this robustness check section, we use single-year UTCI results for comparison. We find increasing heat exposure for children whether we use the triple-year or single-year UTCI information, however the magnitudes of changes are larger in the triple-year comparison.

Figure C.2 demonstrates the change in percentage points (Panel (a)) and percentage (Panel (b)) for the change in the share of time for the average child at or above different UTCI thresholds between the single-years of 1990 and 2020. The three-year equivalent is Figure 1. We point out two major differences in the figures.

First, the single year result under-reports the percentage point change in the share of time that the average child is at or above various UTCI-based heat thresholds across the three decades, comparison to the three-year results, especially on the lower end of UTCI thresholds in warmer seasons. Specifically, between April and September, ⩾26°C UTCI, the change between 1990 to 2020 is approximately 5 percentage points, while the change between 1989-91 to 2019-21 is approximately 8 percentage points. The direction of the percentage point change trend line between the figures remains similar despite the difference in magnitude.

Second, Figure C.2 Panel (b), which shows percentage changes, is dissimilar in magnitude at higher thresholds of heat. The 1990-2020 data would indicate that the largest changes occur at the Moderate Heat and Strong Heat UTCI thresholds, and hold a maximum percentage change of about 18%. Figure 1, Panel (b) of the three-year window analyses indicates that the largest percentage change occurs at the Very Strong

Heat UTCI threshold, with a maximum percentage change of approximately 70%. These differences further demonstrate the single-year results under-counts the shares of time that children are exposed to heat across the 30-year time period compared to the three year results.

Figure C.3 demonstrates the single-year results for the share of children exposed to heat by intensity and duration between 1990 (Panel (a)) and 2020 (Panel (b)). Panel (c) displays the percentage point differences. Figure C.3 three-year analogue is Figure 2. Comparing the results of Figure C.3 Panel (c) to its three-year analogue, the 1990-2020 single-year results are generally lower. The magnitude of these differences range widely. In 1990 (Figure C.3 Panel (a)) and 2020 (Figure C.3 Panel (b)), respectively, 0.1% and 0.6% of children experienced at least 20% of their hours, or over at least 10 weeks, in at-least-Strong-Heat. In the three-year analyses, these values are 0.2% and 0.8%, a net difference of 0.1 percentage point depending on the data used. A more stark example is at the UTCI threshold of $\geqslant$36°C. In 1990 and 2020, the percent of children experiencing at least 4% of their time, or at least 2 weeks, is 27.2% and 32.4%. In the three-year results, these values are 15.0% and 40.8%, a net difference of more than 20 percentage points. Comparing Panels (a) and (b) in both figures, 1990 appears to have been a slightly hotter single year for its time, while 2020 was a slightly cooler single year.

Figure C.4 displays our decomposed change in the share of time for the average child at risk of heat exposure using the single-year UTCI data for 1990 and 2020. Figure C.4 is lower in magnitude compared to its three-year counterpart in Figure 3, respectively, starting at about 2.7 percentage points change versus about 4.5 percentage points at $\geqslant$26°C. We also find that the shift in the spatial population distribution of children over time and the climate shift in the heat exposure across location over time have both contributed to the increase in heat exposure for children. However, the contribution of this population factor to the overall changes is smaller in the triple-year result. In the single-year result at UTCI thresholds of at-least-Strong-Heat ($\geqslant$32°C) and at-least-Moderate-Heat ($\geqslant$26°C), we find that heat exposure across space contributes

to 42% and 40% of the actual change respectively. In the triple-year result, heat exposure contributes a percent increase of 70% and 62% of these same actual changes, respectively. Analysis of the single-year data thus results a climate effect of less than half of the overall effect between 1990 and 2020, while the three-year data results in a climate effect of approximately two-thirds the overall effect.

Figure C.5 displays the regional share of time for the average child at risk of heat exposure using the single-year UTCI data. Panel (c) in particular depicts the percentage point difference in regional heat exposure for children between 1990 and 2020. Central China appears to have almost no difference in heat exposure between the single-years of 1990 and 2020. However, in comparison to our three-year results in Figure 4, Central China actually does experience approximately 3 percentage points of at $\geqslant 26°$C. Similarly, the single-year results under-estimate percentage-point changes in Eastern and Western China relative to the three-year results. These regional decomposition comparisons indicate that different regions experience more heat variation across different time windows compared to other regions in China.

Figure C.2: Change in Share of Time for the Average Child at or above UTCI Thresholds for Children 1990-2020

(a) Changes between 1990 and 2020 in percentage points

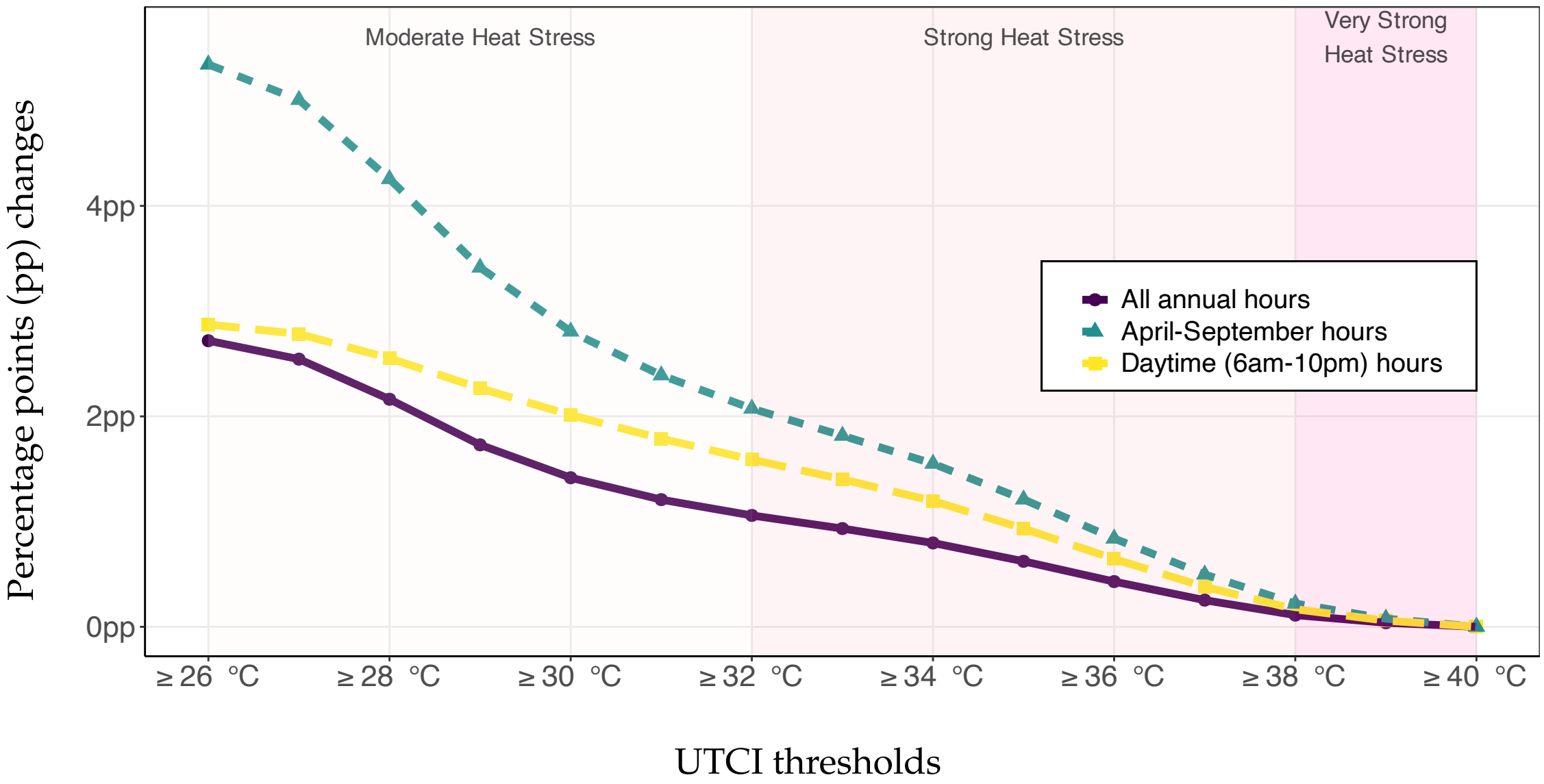

(b) Changes between 1990 and 2020 in percentage

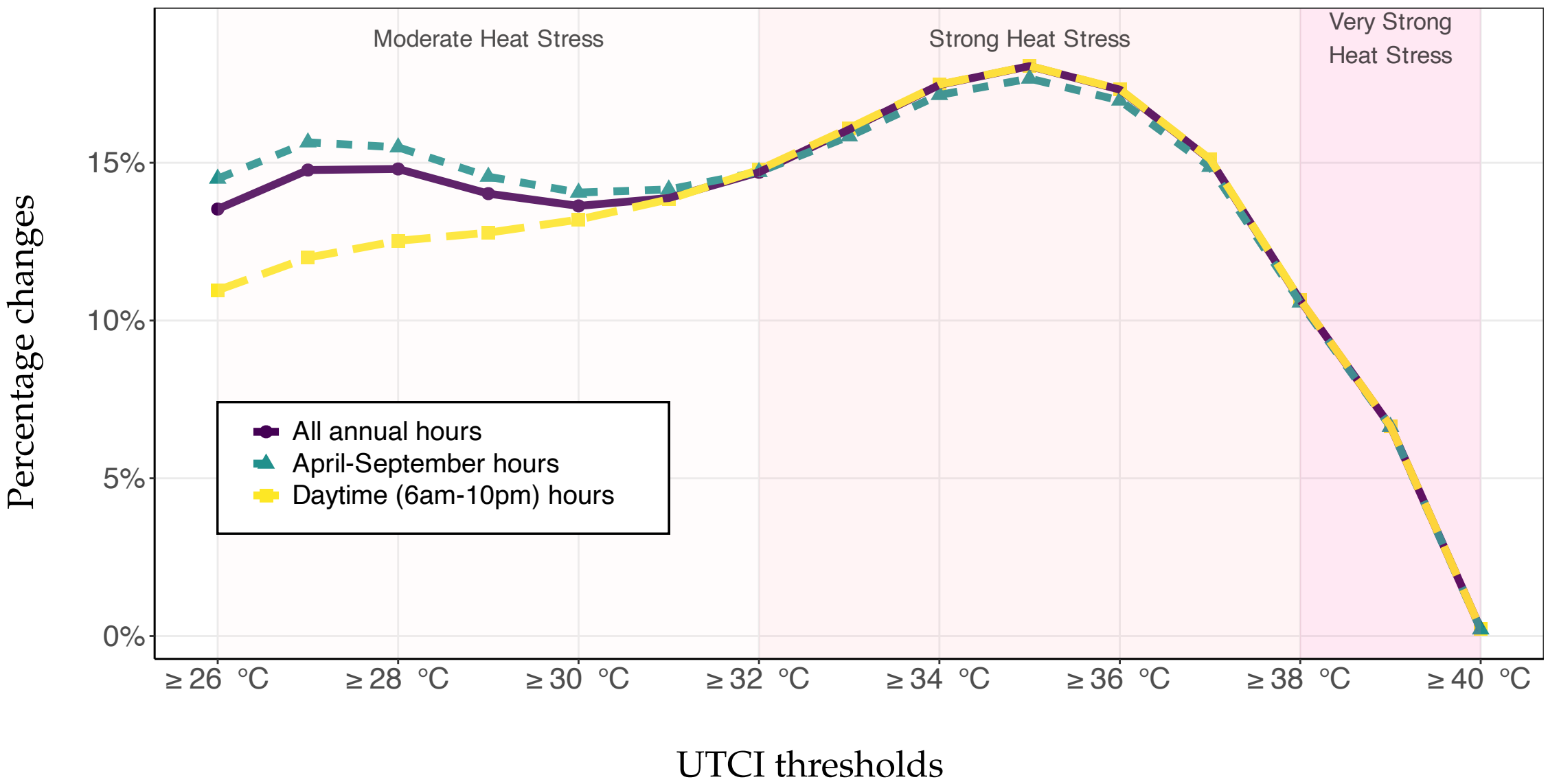

Notes: Panel (a) displays the percentage points (pp) change in the Share of Time for the Average Child at or above UTCI thresholds between 1990 and 2020, while Panel (b) displays the percentage changes in this share. The purple solid line represents results for all annual hours, the green short-dash line for the summer season (April to September), and the yellow long-dash line for daytime hours (6 am to 10 pm). Background colors indicate UTCI heat exposure categories. UTCI values above 26 to 32 degrees Celsius indicate moderate heat (light pink), from 32 to 38 degrees Celsius indicate strong heat (pink), and from 38 to 46 degrees Celsius indicate very strong heat (dark pink).

Figure C.3: Share of Children Exposed to Heat by Intensity and Duration

(a) 1990

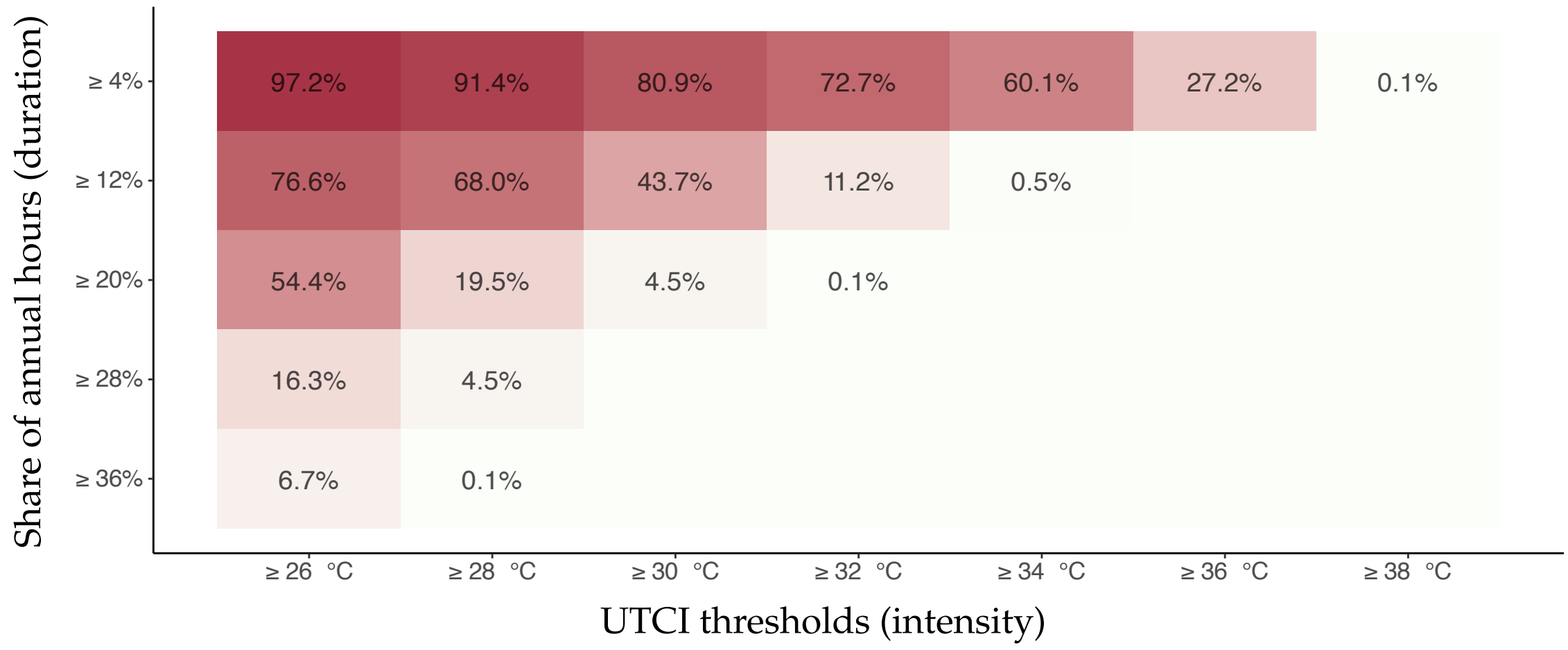

(b) 2020

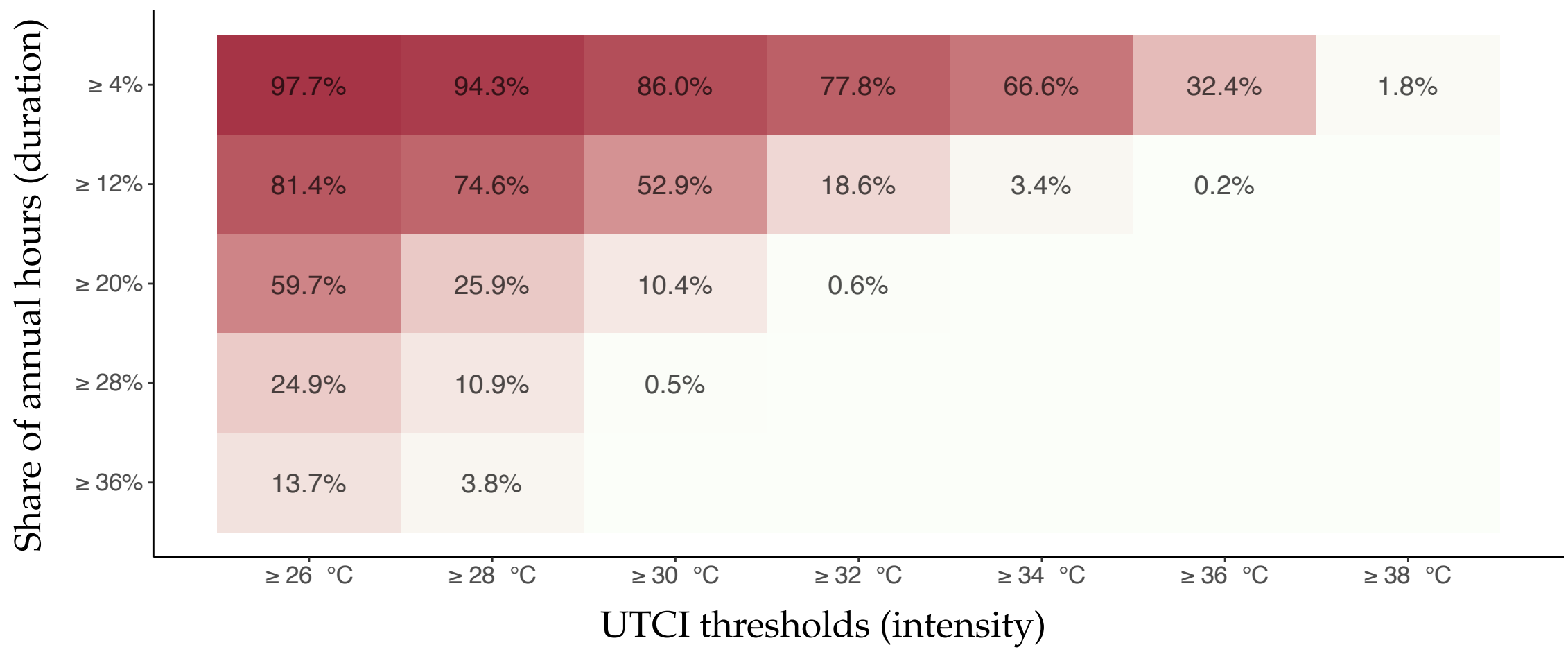

(c) Changes between 1990 and 2020 in percentage points

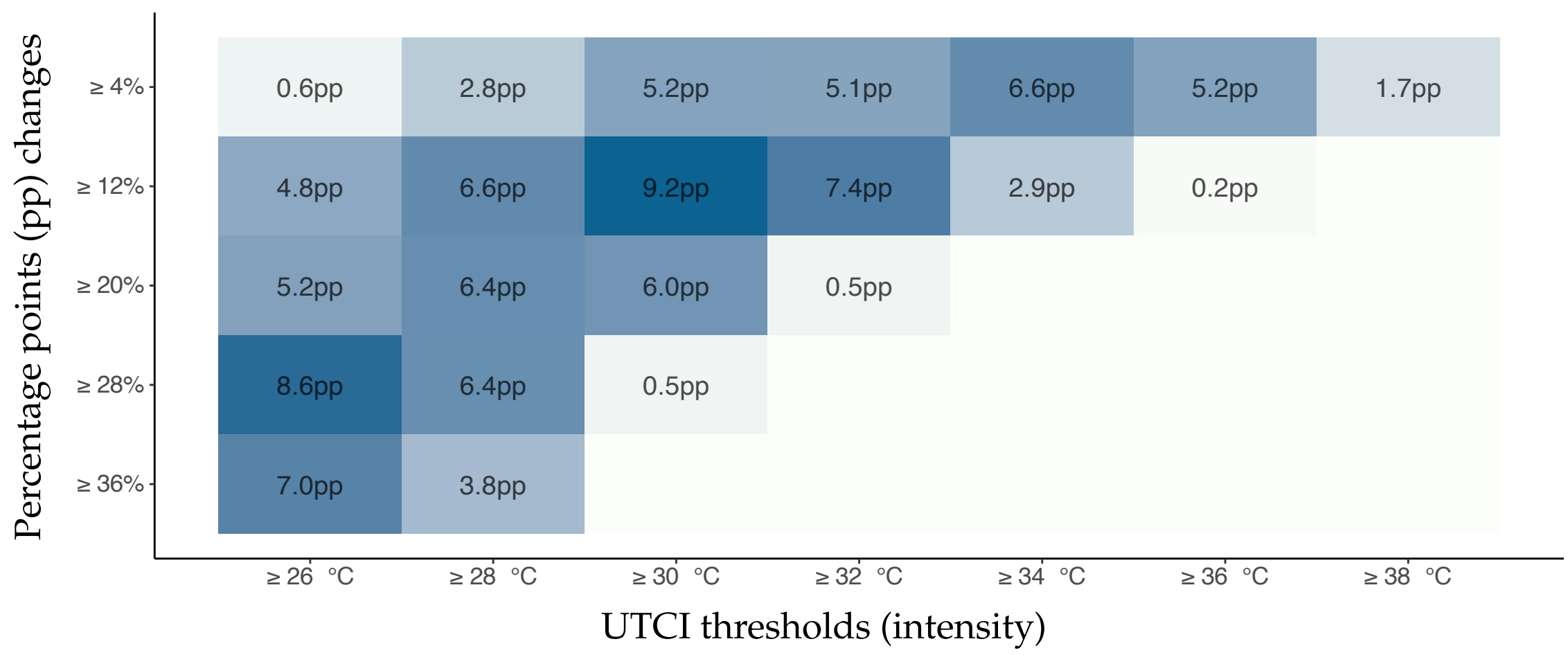

Notes: Panel (a) presents the share of children at risk of heat exposure by duration and intensity in 1990. The duration is measured as the share of annual hours, whereas the intensity is measured as being at or above the UTCI threshold. Panel (b) presents the share of children at risk of heat exposure by duration and intensity in 2020. Panel (c) presents the percentage points (pp) changes of children at risk of heat exposure by duration and intensity between 1990 and 2020.

Figure C.4: Decomposed Change in Share of Time for the Average Child at Risk of Heat Exposure

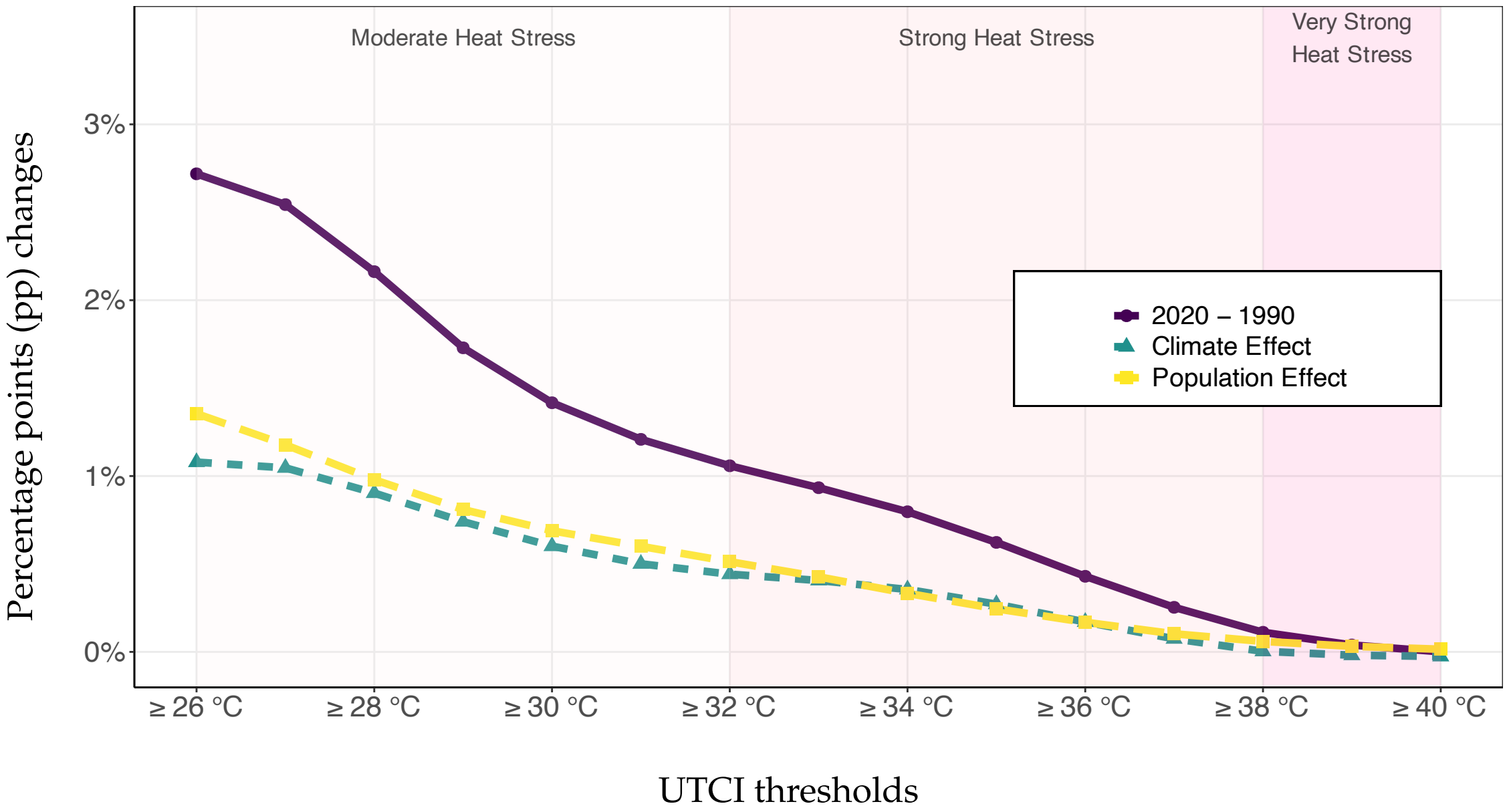

Notes: The purple solid line represents the percentage point difference in the share of time for the average child at risk of heat exposure for children aged 0 to 14 between 1990 and 2020 (with 1990 as the baseline year). In the first counterfactual decomposition, we fix the children's population distribution in 1990 with the observed Universal Thermal Climate Index (UTCI) in 2020. The green short-dash line, or the climate effect, represents the percentage difference between the first counterfactual decomposition results and the baseline (using 1990 children's population distribution with 1990 UTCI). In the second counterfactual decomposition, we fix the UTCI at the 1990 level and use the children's population distribution in 2020. The yellow long-dash line, or the population effect, represents the percentage difference between the second counterfactual decomposition results and the baseline.

Figure C.5: Regional Share of Time for the Average Child at Risk of Heat Exposure

(a) 1990

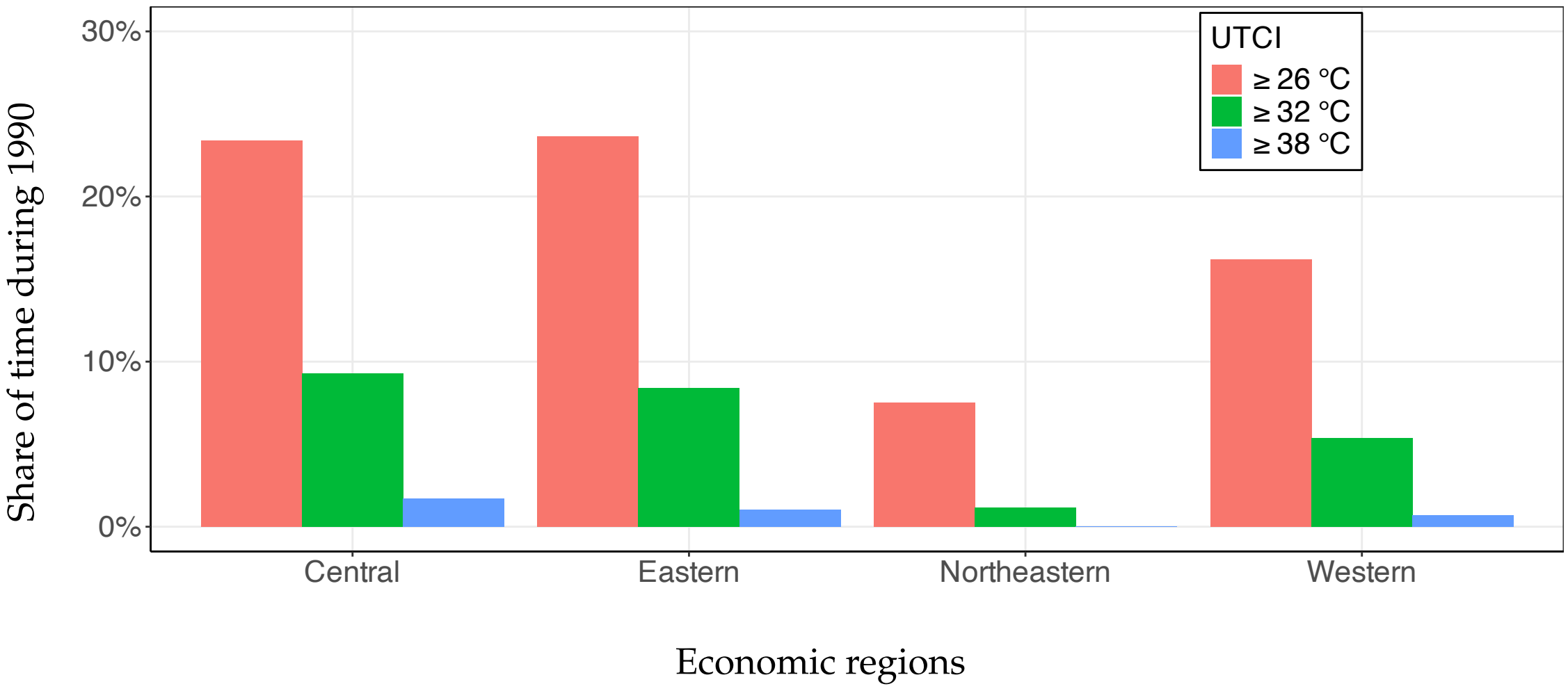

(b) 2020

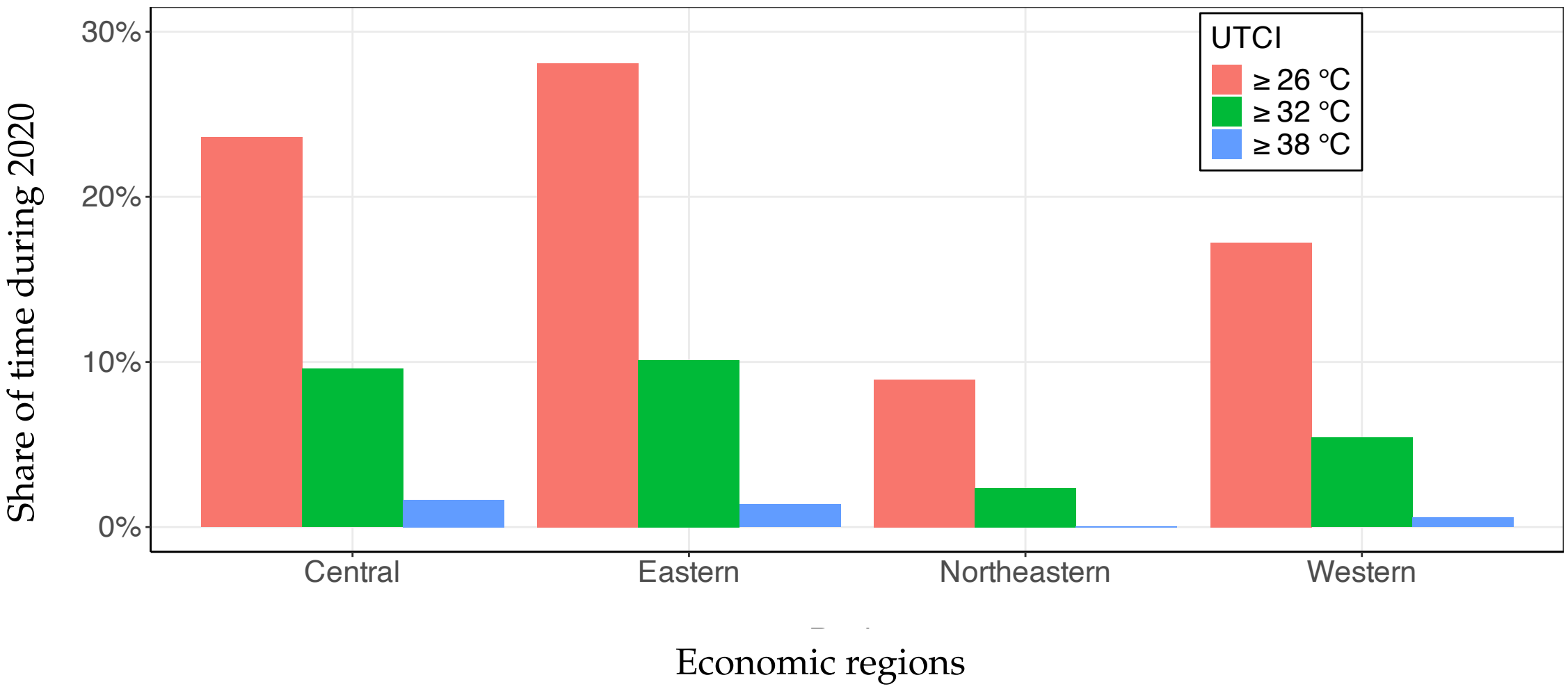

(c) Changes between 1990 and 2020 in percentage points

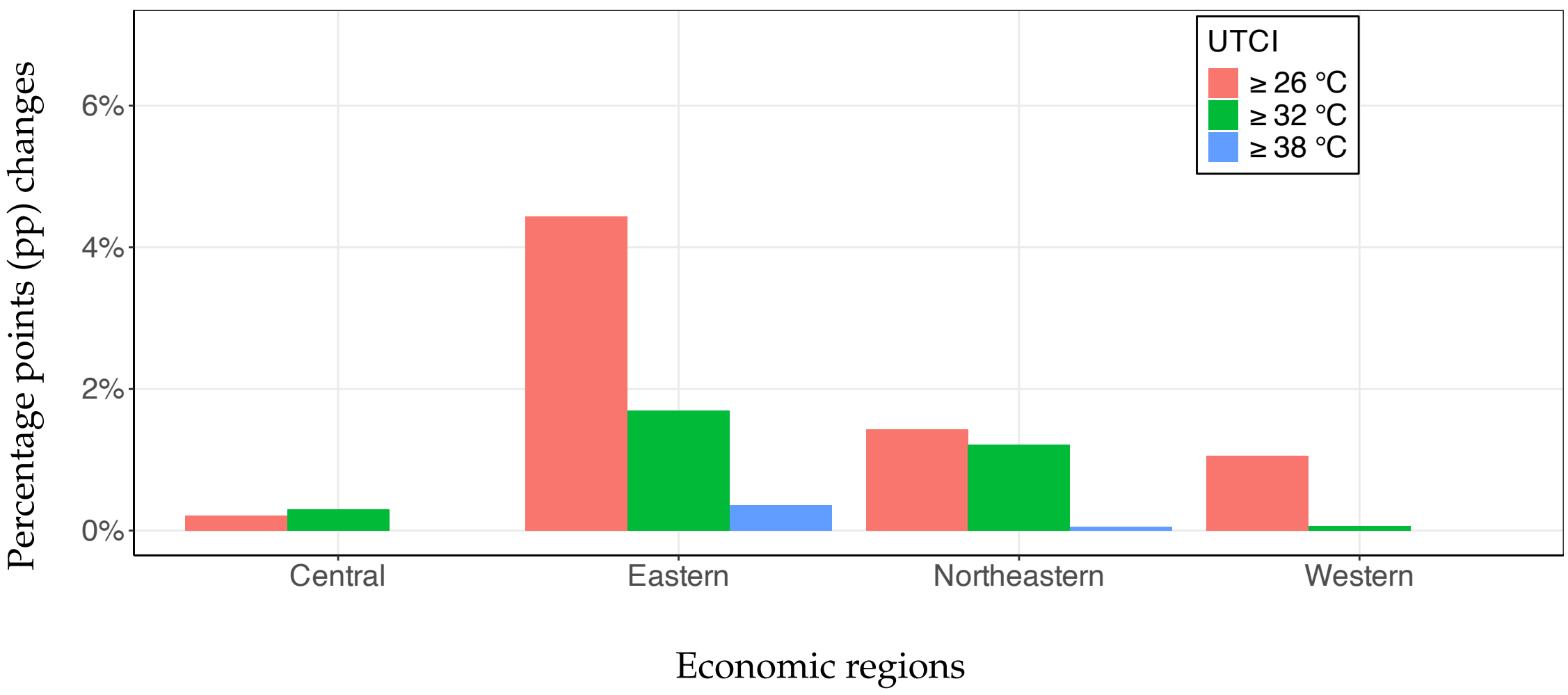

Notes: The y-axis depicts the percentage point difference from 2020 and 1990 heat exposure for children ages 0-14. We display these differences across the four economic regions of China and across different thresholds of heat exposure (moderate, strong, and very strong).

References for Supplementary Information

[1] Emily Hannum, Jeonghyeok Kim, and Fan Wang. *From Population Growth to Demographic Scarcity: Emerging Challenges to Global Primary Education Provision in the Twenty-First Century*. Working Paper. May 2024.

[2] Claudia Di Napoli. *Thermal Comfort Indices Derived from ERA5 Reanalysis*. 2020. DOI: 10.24381/CDS.553B7518.

[3] Claudia Di Napoli et al. "ERA5-HEAT: A Global Gridded Historical Dataset of Human Thermal Comfort Indices from Climate Reanalysis". In: *Geoscience Data Journal* 8.1 (2021), pp. 2–10. ISSN: 2049-6060. DOI: 10.1002/gdj3.102.

[4] Peter Bröde et al. "Deriving the Operational Procedure for the Universal Thermal Climate Index (UTCI)". In: *International Journal of Biometeorology* 56.3 (May 1, 2012), pp. 481–494. ISSN: 1432-1254. DOI: 10.1007/s00484-011-0454-1.

[5] Gerd Jendritzky, Richard de Dear, and George Havenith. "UTCI—Why Another Thermal Index?" In: *International Journal of Biometeorology* 56.3 (May 1, 2012), pp. 421–428. ISSN: 1432-1254. DOI: 10.1007/s00484-011-0513-7.

[6] Krzysztof Blazejczyk et al. "An Introduction to the Universal Thermal Climate Index (UTCI)". In: (Jan. 2013). DOI: 10.7163/GPol.2013.1'].

[7] Gerd Jendritzky and Peter Höppe. "The UTCI and the ISB". In: *International Journal of Biometeorology* 61.1 (Sept. 1, 2017), pp. 23–27. ISSN: 1432-1254. DOI: 10.1007/s00484-017-1390-5.

[8] Beijing Hua tong ren shi chang xin xi you xian ze ren gong si. *1990 County Boundaries of China with Population Census Data, Part 1 & 2. [Shapefile].* 2005.

[9] Beijing Hua tong ren shi chang xin xi you xian ze ren gong si. *China Historical 2000 County Population Census Data*. 2005.

[10] China Data Lab. *China County Map with 2000-2010 Population Census Data*. In collab. with China Data Lab. 2020. DOI: 10.7910/DVN/VKGEBX.

[11] All China Market Research Ltd. *2020 China County Population Census Data with GIS Maps*. 2022.

[12] National Bureau of Statistics of China. *[Method of Dividing the East, West, Central and Northeast Regions]*. Beijing: State Council of China, June 13, 2011.

[13] Edzer Pebesma. "Simple Features for R: Standardized Support for Spatial Vector Data". In: *The R Journal* 10.1 (2018), pp. 439–446. DOI: 10.32614/RJ-2018-009.
